# Phylogeny Disambiguates the Evolution of Heat-Shock *cis*-Regulatory Elements in *Drosophila*

**DOI:** 10.1371/journal.pone.0010669

**Published:** 2010-05-17

**Authors:** Sibo Tian, Robert A. Haney, Martin E. Feder

**Affiliations:** Department of Organismal Biology and Anatomy, University of Chicago, Chicago, Illinois, United States of America; National University of Ireland Galway, Ireland

## Abstract

Heat-shock genes have a well-studied control mechanism for their expression that is mediated through *cis*-regulatory motifs known as heat-shock elements (HSEs). The evolution of important features of this control mechanism has not been investigated in detail, however. Here we exploit the genome sequencing of multiple *Drosophila* species, combined with a wealth of available information on the structure and function of HSEs in *D. melanogaster*, to undertake this investigation. We find that in single-copy heat shock genes, entire HSEs have evolved or disappeared 14 times, and the phylogenetic approach bounds the timing and direction of these evolutionary events in relation to speciation. In contrast, in the multi-copy gene *Hsp70*, the number of HSEs is nearly constant across species. HSEs evolve in size, position, and sequence within heat-shock promoters. In turn, functional significance of certain features is implicated by preservation despite this evolutionary change; these features include tail-to-tail arrangements of HSEs, gapped HSEs, and the presence or absence of entire HSEs. The variation among *Drosophila* species indicates that the *cis*-regulatory encoding of responsiveness to heat and other stresses is diverse. The broad dimensions of variation uncovered are particularly important as they suggest a substantial challenge for functional studies.

## Introduction

Although *cis*-acting sequences are clearly vital to the regulation of gene expression and its evolution [1], the decoding of the organizational basis for this regulation and evolution is still a work in progress despite considerable effort, numerous research studies, and substantial genomic data. The difficulty of this task resides in the nature of the code itself: it is irregular, contains numerous synonyms and exceptions, and no rule appears to go unviolated [2]. We suggest, as have others [3] that a way forward may be in the combination of two aspects: a *cis*-regulatory sequence whose mechanism of controlling gene expression is understood in detail, and a robust phylogeny of successively more distantly related species. The former feature links explicit consequences for gene expression to variation in the *cis*-regulatory sequence. A phylogenetic approach has two advantages beyond “phylogenetic footprinting”; i.e., the identification of putative *cis*-regulatory sequence through conservation [4]. First, it poses specific hypotheses about where in evolution changes have arisen; these hypotheses may in turn anchor other hypotheses about specific genetic mechanisms of evolutionary change and the adaptive consequences of the change. Second, it highlights which features of the *cis*-regulatory sequence are free to evolve and which are stabilized by selection, providing corroboration for functional studies but also possibly suggesting other, unrecognized features of the functional mechanism for further testing [5]–[7]. Here we exemplify these points in the *cis*-regulatory sequences governing the heat-shock response, heat-shock elements (HSEs).

**Figure 1:**
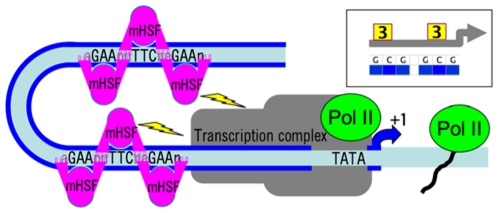
Binding of Heat-Shock Factor (HSF) trimers to Heat Shock Elements (HSEs) mediate the transcription of heat-shock genes. This cartoon, greatly simplified from [33], Fig. 11 of [13], and Fig. 4 of [31], depicts two HSEs, each comprising three consecutive 5 bp sequences of n**G**AAn and nTT**C**n in alternation and each binding an HSF trimer. The HSF monomers (mHSF) interact with one another via a trimerization domain and the “wing” of the winged helix-loop-helix motif. In HSF, Helix 3, whose sequence is conserved in eukaryotes, is responsible for the specificity of interaction with the HSEs. Once bound, HSF trimers interact with the transcriptional complex, including RNA polymerase II (Pol II) to result in gene expression. See Introduction for additional explanation. Inset: symbolic representation of the promoter organization, as will appear in subsequent figures. Aspects omitted from the cartoon include HSEs longer than three 5 bp units, which support cooperative binding of HSF trimers, variation in sequence within HSEs, variation in position and spacing of HSEs, GAGA factor and its binding sites, other cis-regulatory elements, nucleosomes and their acetylation sites, and numerous proteins involved in transcriptional regulation.

HSEs are among the best-characterized and simplest of *cis*-regulatory elements (CREs) in metazoans (Figure 1); this and the nature of the heat-shock response itself provide an exceptionally firm foundation for analysis. In brief, heat, other proteotoxic stresses, and diverse other signals change gene expression dramatically. In *Drosophila*, the transcription of most active genes is halted [8] and a restricted set of heat-shock genes, many encoding molecular chaperones and including HSEs in their proximal promoter regions, are coordinately upregulated during or after heat-stress [9]. Unlike in many other promoters, experimentally-verified HSEs of *Drosophila* are rarely beyond ∼500 bp of the transcription start site, and this relatively short proximal promoter suffices for full-strength transcription [10].

HSEs are binding sites for HSFs (heat-shock factors, Figure 1), transcription factors whose trimerization mediates the heat-shock response in eukaryotes [11], [12] in combination with numerous co-regulators [13]. In many higher eukaryotes HSFs are multiple, with family members varying in stimuli for their activation, affinity for HSEs, and downstream targets [14], [15]. Fortuitously for the present study, HSF is singular in *Drosophila* [16] and highly conserved [17]. [This contrasts with the co-evolution of CRE binding sites and TFs both in other HSFs and for other TFs (eg. [18])]

The DNA binding domain of HSF contains an antiparallel β sheet and a cluster of three α helices, one of which (helix 3) functions in DNA recognition in the context of a helix-turn-helix structure [19]. This domain is itself highly conserved [17], implying that variation in HSEs is primarily if not exclusively responsible for variation in HSF-HSE interaction in *Drosophila*.

Well-defined features of the HSE sequence seem critical for HSF-HSE binding. In flies as in other taxa, the canonical HSE comprises inverted repeats of the pentanucleotide sequence 5′-NGAAN-3′, where N is any nucleotide [20], and typically is within 400 bp upstream of the transcriptional start site. HSEs often contain at least 3 continuous 5 bp subunits alternating between NGAAN or NTTCN or vice versa, with each 5 bp HSE subunit capable of binding one monomer of the HSF trimer [20]–[22]. The alternating multiple subunits promote cooperative binding of HSF trimers in the major groove of the DNA helix [23], which correlates with more stable protein-protein and DNA-protein interactions and stronger heat shock responses [24], [25]. In general, the number of consecutive 5 bp units relates to the magnitude of the heat-shock response. However, in yeast, HSEs of equal length (4 subunits) and homologous to the consensus have appreciably different binding properties depending on which subunit variant (NTTCN or NGAAN) occupies the most distal position [26]. This difference is due to the ability of the variant beginning with NTTCN to bind an additional HSF trimer [26], and is not evident in experimental studies of *D. melanogaster* [21].

The three central nucleotides, GAA or TTC, are highly conserved in *D. melanogaster* [10], implying they are functionally constrained. Indeed, the 2nd position of an NGAAN motif is critical for binding, with the 3rd and 4th positions having a lesser effect and the 5th position no effect [23]. Mutations in the first position also affect HSF binding [23], however, although the compositional bias is less than for positions 3 or 4. These data suggest the consensus motif should again [see, for example, [22], [27]–[30] as former episodes of redefinition] be redefined as AGAAN [23]. As both HSF activation and the heat-shock response are multistep processes, variation in these processes cannot unilaterally be attributed to variation in HSEs, however.

Finally, HSEs are often multiple. Although HSF-HSE complexes that form near the transcription start site (TSS) can engage the transcriptional apparatus through physical proximity, even more distal HSEs can similarly engage through the bending of the promoter DNA [31]. The approximation of distal HSEs to the TSS is a function of both their position within the promoter and the interaction of GAGA factor and its binding sites, positioned nucleosomes and their acetylation state, and other co-regulators [13], [31], [32].

As with other aspects of the *cis*-regulatory code, HSEs deviate from the canon. In vitro, HSEs with only 2 subunits can form stable interactions with HSF [21], [33]. Furthermore, the 5 bp inverted repeats need not be consecutive. Gapped HSEs, for example, contain internal 5 bp blocks with little or no homology to the canonical motif flanked by canonical sequences in the proper orientation. These HSEs have binding affinity comparable to the minimal functional binding sequence as long as they exceed 4 subunits [20]. In yeast, gapped versions constitute an entire class of HSEs [34]. HSF's affinity for HSEs varies with their sequence and position [13], [35]. HSF also appears to bind promoters that lack either typical or gapped HSEs, and in genes not previously associated with the heat-shock response [34], [36]–[38]. In some cases, recognizable motifs for other transcription factors are present, suggesting that HSF binding to these promoters is mediated differently than when HSEs are present [38].

Here we use this knowledge to elucidate the evolution of *cis*-regulatory sequence in *Drosophila* heat-shock proximal promoters derived from 12 species whose genomes have been sequenced [39], and. We also include data from the island endemic species *D. santomea*, which has diverged substantially from its sister species, *D. yakuba,* in thermal tolerance [40], a phenotype potentially explicable by regulatory divergence in heat-shock promoters. As suggested above, the combination of detailed mechanistic understanding of HSE structure-function and a phylogenetic context reveals hitherto unrecognized features of heat-shock promoters that apparently are under functional constraint. Moreover, where heat-shock promoters have evolved, this same combination defines the nature and timing of the underlying evolutionary events.

## Results

### Phylogeny implicates function

**Figure 2:**
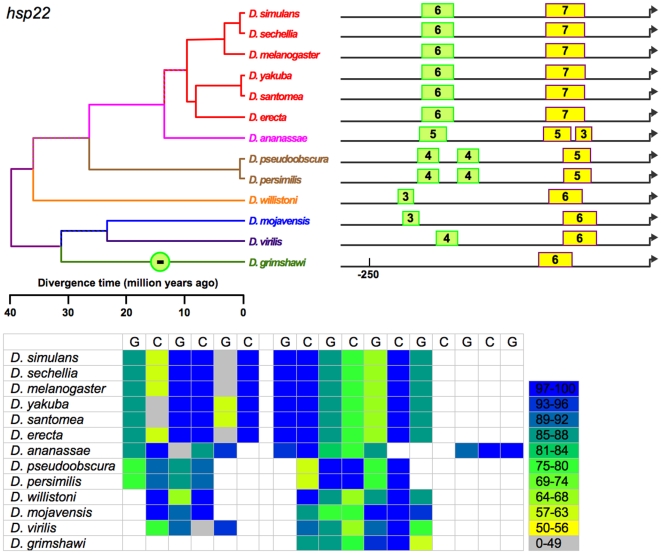
Organization of identified HSEs for *Hsp22* in 13 *Drosophila* species. Numbers indicate length of HSE in 5 bp units. Rectangles represent HSEs, whose length (in 5 bp subunits) is indicated. HSEs of the same color indicate probable homologs. Each circle, arbitrarily indicates a deduced evolutionary gain (+) or loss (−) of an HSE, and is placed arbitrarily at the center of the segment in which it occurs. Below a heat map expresses the similarity of each 5 bp subunit to the canonical sequence: G represents nGAAn-type motifs, while T represents nTTCn-type motifs. Columns correspond to the order of HSEs (from left to right) in the promoter maps. Key to heat maps: the number represents the information content of each actual subunit as a percent of the value calculated for the consensus sequence. Genome alignment indicates all proximal HSEs of 11 species occur in a conserved block near the TSS, with the exception of *D. grimshawi*. These HSEs are colored yellow. Point substitutions and small indels give rise to length variation in HSEs and a small gap in *D. ananassae*. In *D. grimshawi*, two copies of *Hsp22* appear to be present. The copy whose coding sequence most closely aligns with those of the other 11 species lacks a conserved TSS or promoter and may not be expressed. Both a conserved TSS and promoter are present in the second copy, and included in the figure. A distal HSE is present in all species but *D. grimshawi*. These vary in size and distance from the TSS. However, all appear to be orthologous in whole genome alignments, as well as in local alignments with multiple algorithms, with the exception of those in *D. willistoni* and *D. mojavensis*. If correct, this implies parallel losses and re-evolutions of HSEs, which we reject by parsimony. The figure presents a simpler scenario in which only a single loss occurs. In *D. ananassae*, parsimony suggests that the two proximal HSEs arose from insertion into or mutation of a single ancestral HSE.

**Figure 3 pone-0010669-g003:**
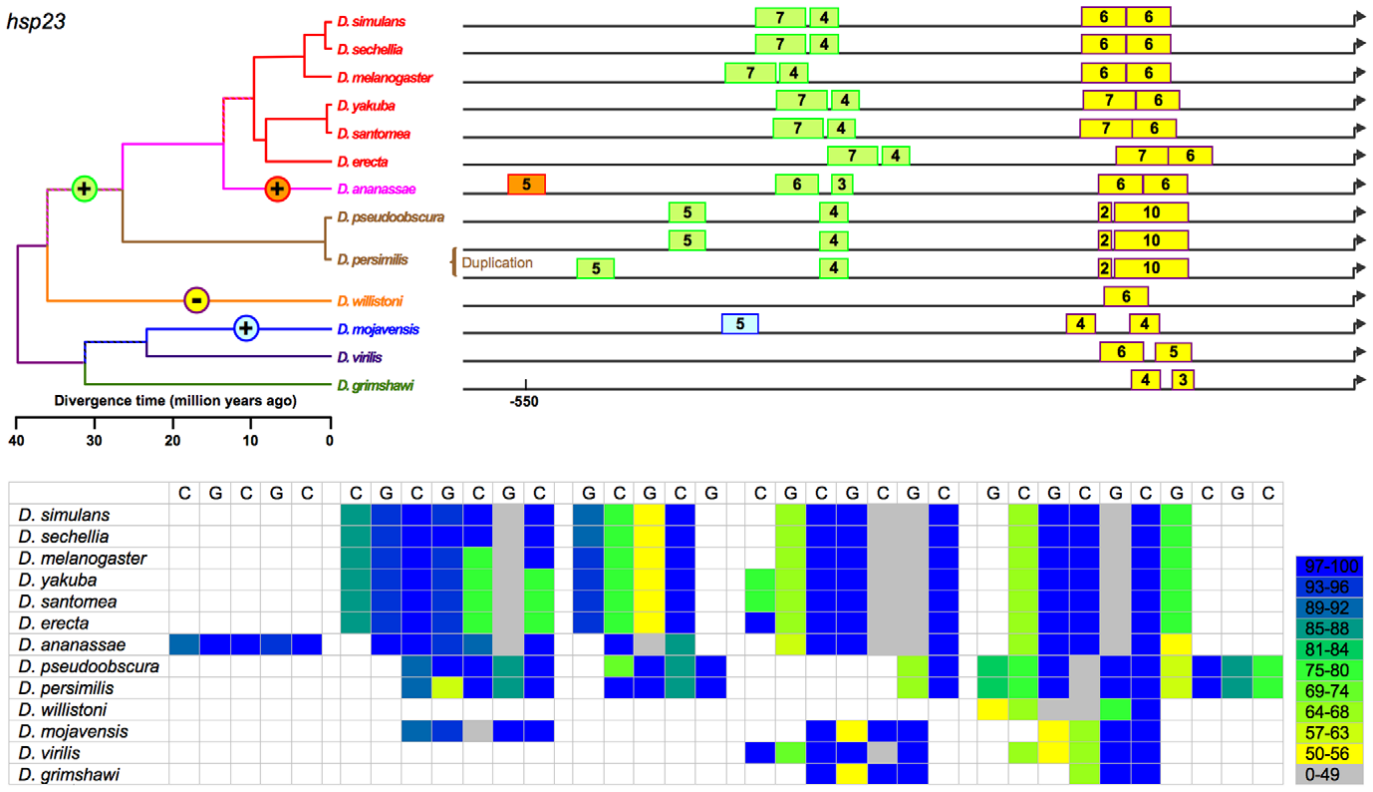
Sizes and positions of identified HSEs for *Hsp23* in 13 *Drosophila* species. Numbers indicate length of HSE in 5 bp units. Genome alignment indicates all proximal HSEs of 12 species occur in a conserved block near the TSS. These HSEs are colored yellow. Parsimony suggests that two proximal HSEs are ancestral, and their variation in relative size and spacing arose from point substitutions and small indels. The absence of one member of the cluster in only one of the 12 species (*D. willistoni)* is therefore considered a loss. A single gain of two distal HSEs in a common ancestor of the *melanogaster-obscura* groups, a gain of a third distal HSE n *D. ananassae* alone, and a gain of a single distal HSE in *D. mojavensis* are more parsimonious than alternative scenarios.

**Figure 4 pone-0010669-g004:**
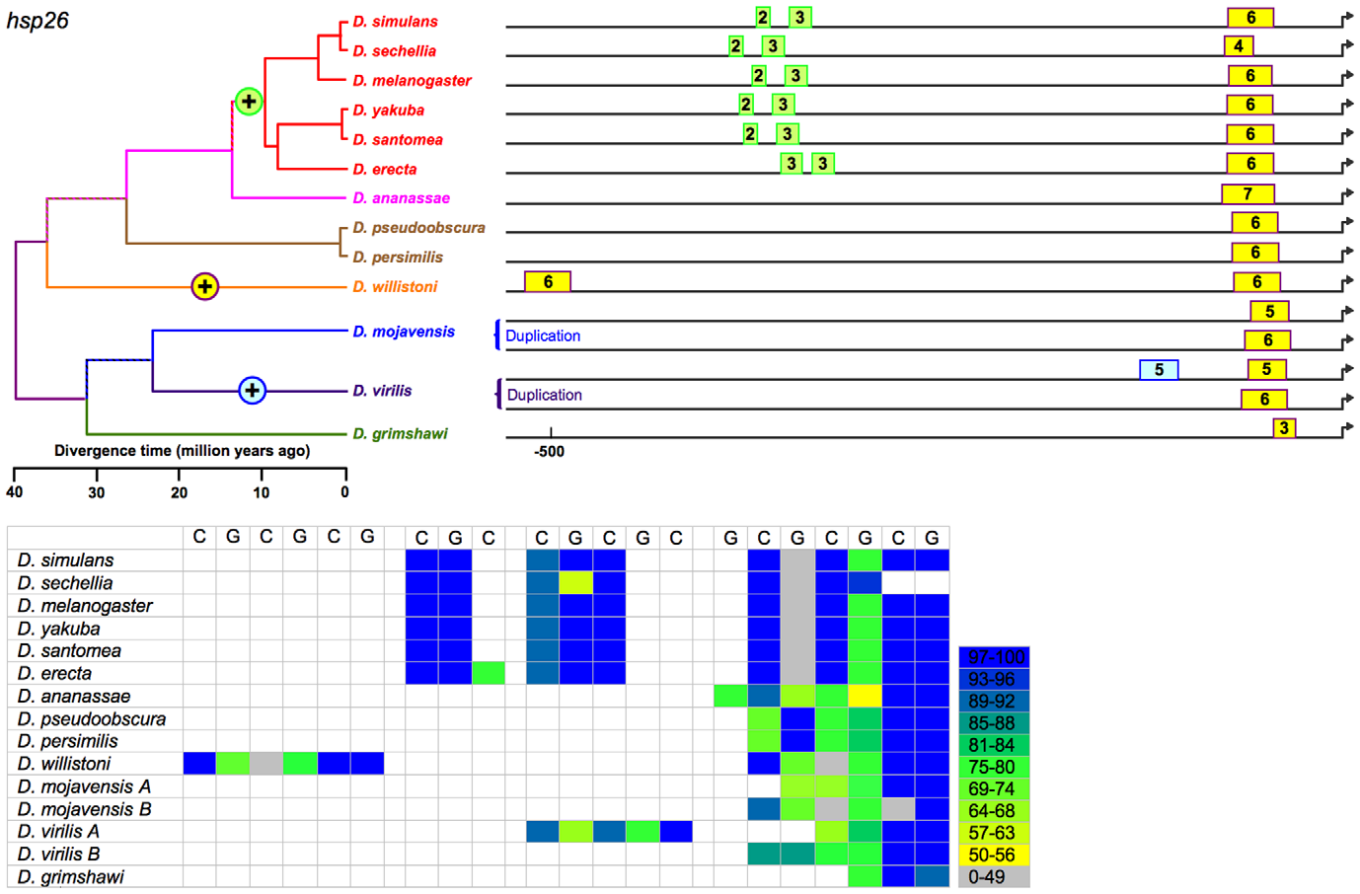
Sizes and positions of identified HSEs for *Hsp26* in 13 *Drosophila* species. Numbers indicate length of HSE in 5 bp units. Genome alignment indicates all proximal HSEs of 12 species occur in a conserved block near the TSS. These HSEs are colored yellow. Length variation in proximal HSEs is due to both point substitutions and small indels. By parsimony, an upstream distal HSE cluster arose in a common ancestor of the *melanogaster* subgroup, and a single distal HSE arose independently in *D. willistoni* and *D. virilis*. The distal element in *D. willistoni* appears due to a local duplication of sequence proximal to the TSS. Duplication of the *Hsp26* coding sequence appears to have occurred in *D. mojavensis* with both copies having a similar proximal promoter architecture.

The 13 species of *Drosophila* exhibit at least 419 computationally identifiable HSEs in the 8 heat-shock genes under study (Table S1, Table S2, Figures 2–9). This estimate includes 223 in usually single-copy genes (*Hsp22*, *Hsp23*, *Hsp26*, *Hsp27*, *DnaJ-1*, *Hsp68*, and *Hsp83*), with the balance in *Hsp70*, which is always multi-copy. These HSEs vary in occurrence, number, position, and conformity with the canonical sequence. For example, HSEs range from two 5 bp subunits, [in *Hsp23*, *Hsp26*, *DnaJ-1*, and *Hsp70* (Figures 3, 4, 6, 8)] to eleven subunits [in *Hsp68* (Figure 7)]. Importantly, phylogenetic conservation in the 13 species suggests that certain features are functionally significant and are therefore subject to selection. These features include:

**Figure 5 pone-0010669-g005:**
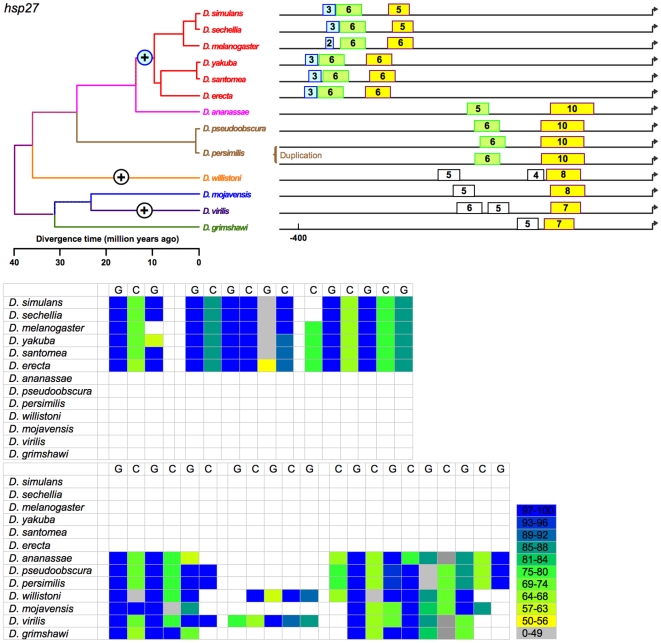
The distribution of HSEs is most variable in *H*s*p27*. In the *melanogaster* subgroup, it is the only heat-shock gene with no HSE within 200 bp of the TSS. The multispecies genome alignment provides support for a rearrangement in *D. ananassae* and one or more deletions in the *obscura* group that remove intervening sequence and explain differences in distance to the TSS. We conservatively assume a single origin of proximal HSE sequence in *Drosophila Hsp27* promoters, with subsequent modifications of length and distance to the TSS. A second, more distal HSE is found in all species, and appears to be orthologous in the *melanogaster* and *obscura* groups. A gain of a third distal HSE has occurred in the melanogaster subgroup. *D. willistoni* and *D. virilis* also have a third HSE, suggesting lineage specific gains. However, although the regions compared are directly adjacent to an orthologous coding region, ambiguity exists in the relationships of individual HSE in *D. willistoni* and the species of the subgenus *Drosophila*, and these HSEs are colored white.

**Figure 6 pone-0010669-g006:**
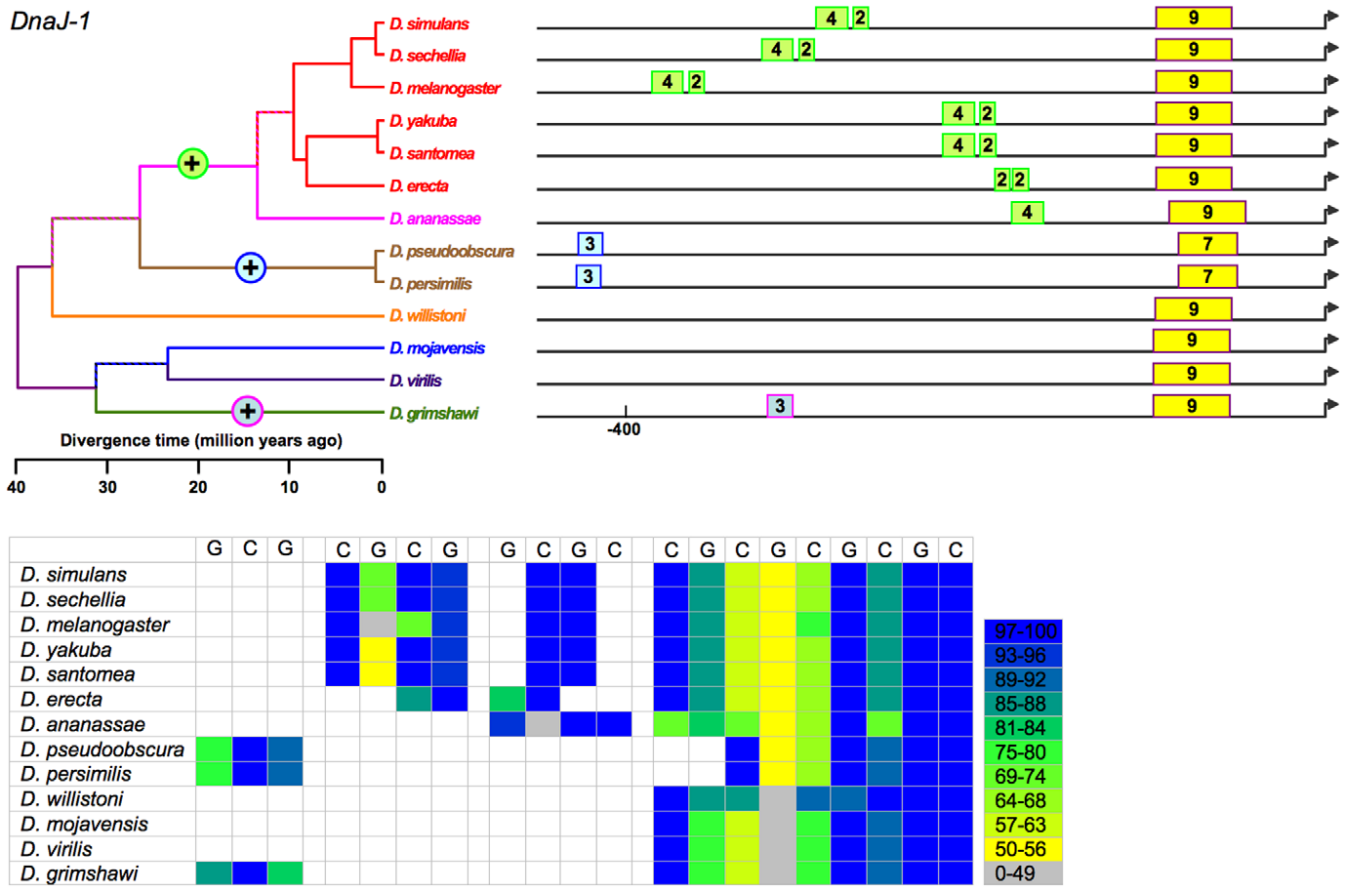
Sizes and positions of identified HSEs for *DNAJ-1* in 13 *Drosophila* species. Numbers indicate length of HSE in 5 bp units. Genome alignment indicates all proximal HSEs of 12 species occur in a conserved block near the TSS, with the exception of *D. willistoni*. However, local alignments upstream of the DNAJ-1 ortholog in this species indicate conservation of this HSE. Conserved proximal HSEs are colored yellow. Minor length variation in proximal HSEs are presumably due to small indels and point substitutions. Although divergence in the *D. ananassae* promoter region ambiguates the orthology of its distal HSE, its location, size, and parsimony suggest a single origin of a distal HSEs in a common ancestor of the *melanogaster* group. Distal HSEs arose independently in the *obscura* group and in *D. grimshawi*.

**Figure 7 pone-0010669-g007:**
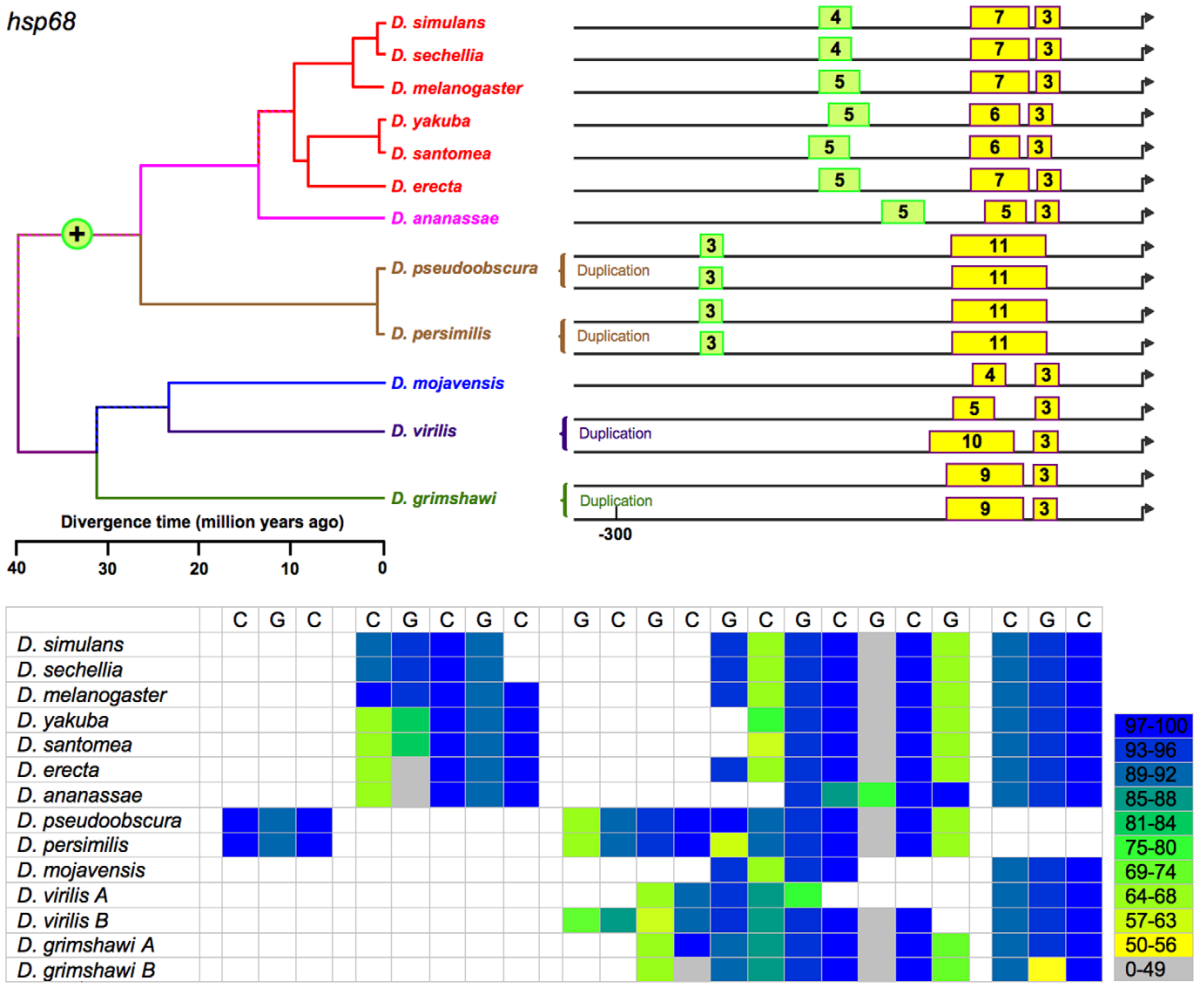
Sizes and positions of identified HSEs for *Hsp68* in 13 *Drosophila* species. Numbers indicate length of HSE in 5 bp units. Multispecies genome alignment juxtaposes *Hsp68* and *Hsp70* copies in some species. Hence, all coding regions called as *Hsp68* in this study were confirmed through amino-acid states at diagnostic sites following Kellet and McKechnie (2005), and local alignments were used to assess conservation in some species. No recognizable copy of *Hsp68* was found in *D. willistoni*. All proximal HSEs occur in a conserved block near the TSS. Upstream regions are divergent, and no distal HSEs are recognized outside the *melanogaster* and *obscura* groups. High levels of sequence divergence in the regions including and flanking the distal HSEs ambiguate the orthology of the HSEs between the *melanogaster* and *obscura* groups. Parsimony implicates a single gain of a distal HSE rather than two independent gains. Finally, the distal HSE could have arisen in the common ancestor of the *melanogaster* and *obscura* groups, or it could have arisen in the common ancestor of all species and been lost in the common ancestor of *D. mojavensis*, *D. virilis*, and *D. grimshawi*. Parsimony supports the former scenario.

**Figure 8 pone-0010669-g008:**
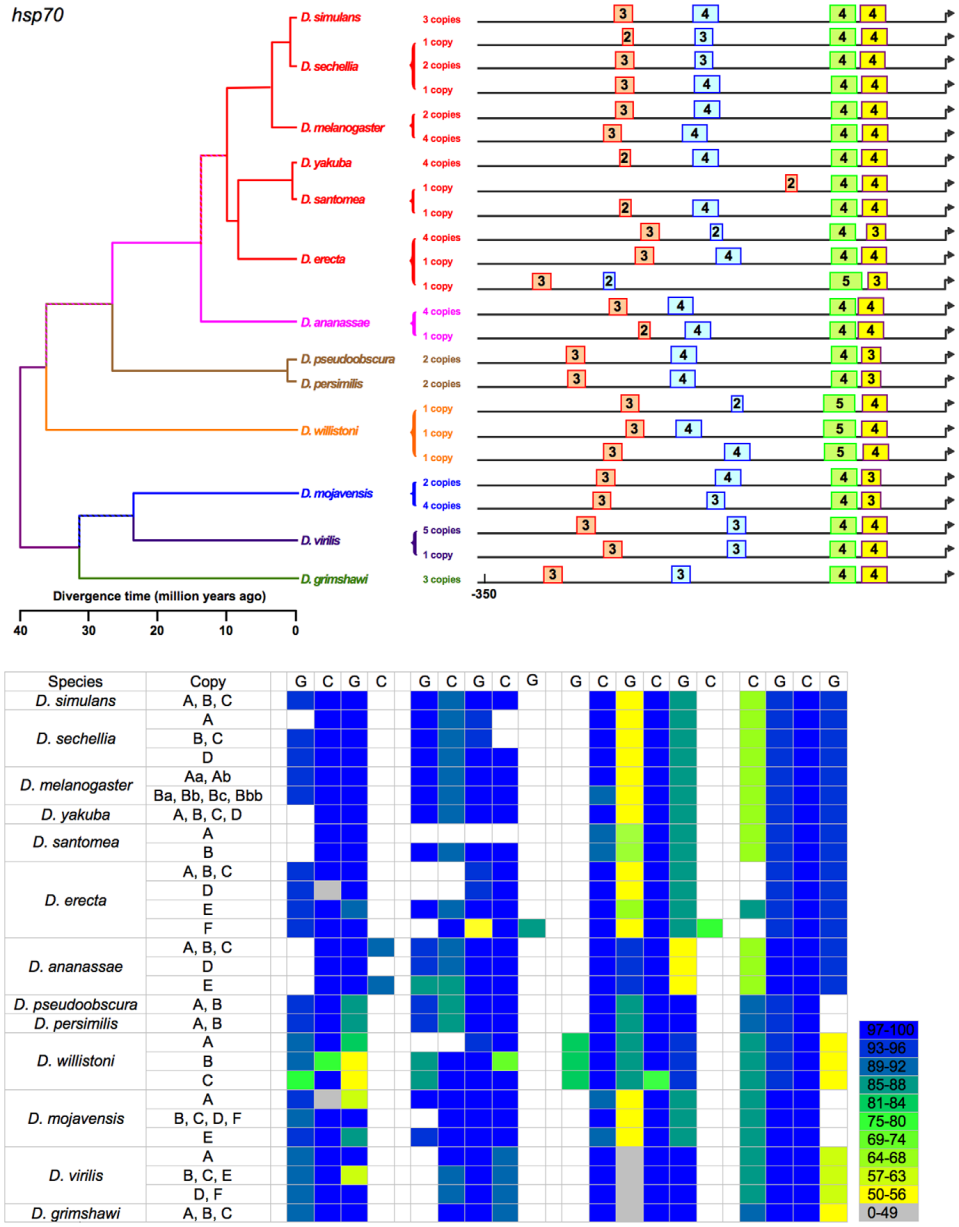
Sizes and positions of identified HSEs for all identified copies of *Hsp70* in 13 *Drosophila* species. The number of HSEs is nearly constant across copies in all species. Proximal HSEs are nearly invariant in position, and differ minimally in length. There is a larger amount of variation in the position of distal HSEs, but only a single gain/loss of HSEs occurs, in one copy of *Hsp70* from *D. santomea*.

**Figure 9:**
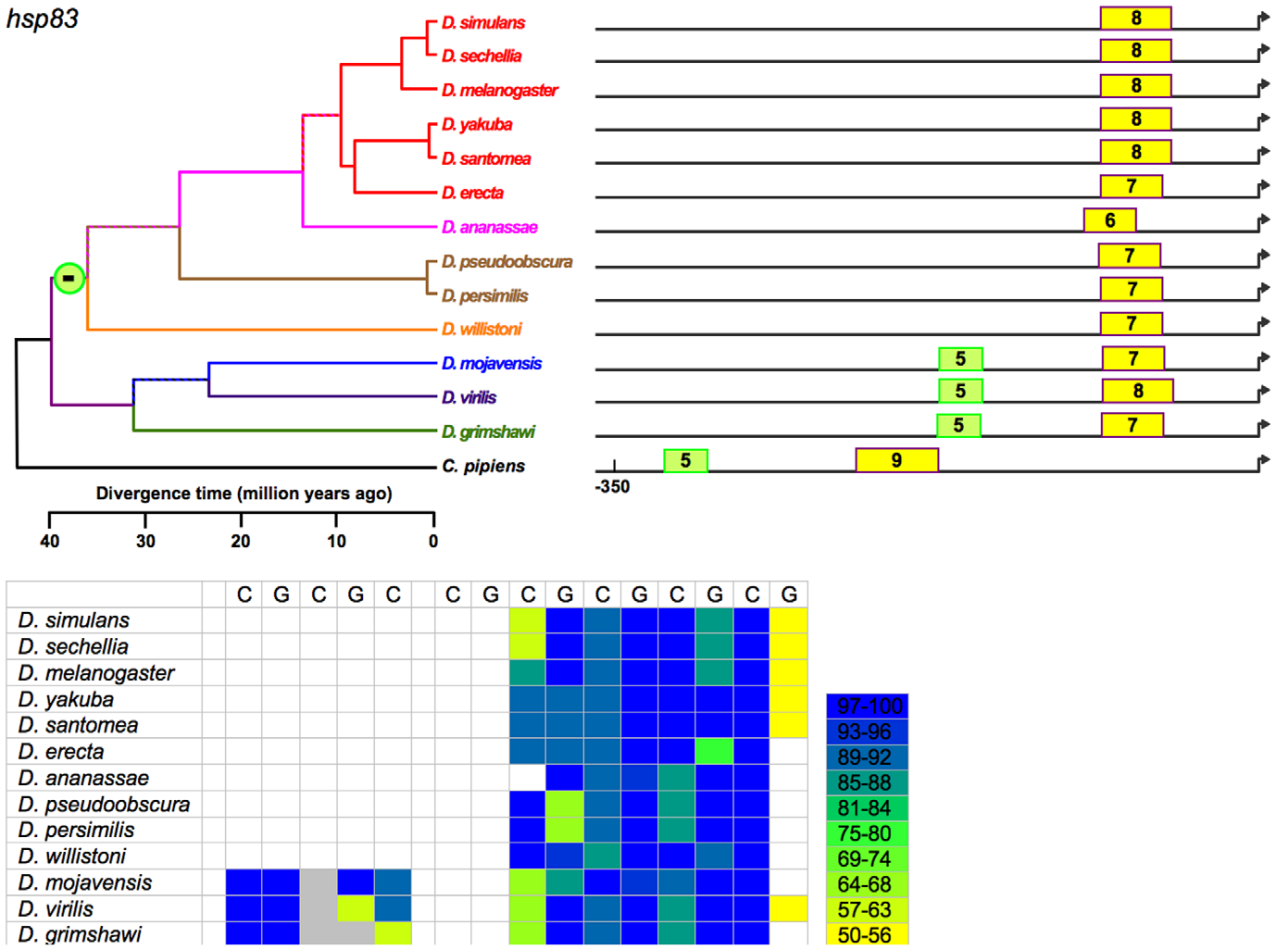
Sizes and positions of identified HSEs for *Hsp83* in 13 *Drosophila* species. Numbers indicate length of HSE in 5 bp units. Genome alignment indicates all proximal HSEs of 12 species occur in a conserved block near the TSS. These HSEs are colored yellow. Length variation in proximal HSEs is due to both point substitutions and small indels. A distal HSE in the subgenus *Drosophila* only is equally consistent with a single gain in this subgenus or a single loss of this HSE in Sophophora. Sequence of an outgroup (*Culex pipiens*) implicates the latter scenario by parsimony.

(a) Putative HSEs that previously had been overlooked. In *Hsp26*, for example, a third (and putative) HSE consisting of 2–3 5 bp units occurs in all *melanogaster* subgroup (Figure 4) species. This sequence was observed previously in *D. melanogaster* but reasonably declared not an HSE at that time [41]. Its conservation in 6 species for >10 MY suggests that this conclusion should be revisited.

(b) Tail-to-tail configurations, which are preferred and persist in evolution. Identified HSEs are both head-to-head (an HSE with a nGAAn-type motif at its most distal site) and tail-to-tail (an HSE in which nTTCn is the most distal subunit) [26]. The tail-to-tail configuration is preferred, however. Of the 393 HSEs in the analysis, 236 began with nTTCn while only 157 began with nGAAn (G-test: G = 15.19, df = 1, p<0.0001), despite an overall ratio of nTTCn-type subunits to nGAAn-type of 1.01. The orientation of an HSE appears conserved across species. This is true of most HSEs (Figures 2–9) and is especially clear in the proximal HSE of *Hsp83*.

(c) Gapped HSEs, which are common and conserved across species. We classify HSEs as gapped if one or more internal 5 bp subunits score <50% of the maximum possible for their motif type, and are flanked by subunits scoring >50%. Despite prior treatment in the literature as anomalies (see Introduction), gapped HSEs are commonplace in the 13 species of *Drosophila*. Gapped HSEs occur in every gene in our data set**,** in both proximal and distal HSEs, and in all species. The gapping, moreover, is conserved, appearing as light stripes in the heat maps in Figures 2–9. In *Hsp23*, for example, a low-scoring 5 bp unit (in grey) persists in 2 separate HSEs in *melanogaster* group (Figure 3) species diverging as much as 12 MYA, as do 2 tandem 5 bp units in the second most proximal HSE.

(d) Diversity in the organization of heat-shock promoters. Overall, species vary in the aggregate length of HSE sequence in their heat-shock promoter regions (G-test: G = 22.810, df = 11, p<0.019). Few if any heat-shock promoters are identical in the number, arrangement, and size of their HSEs (except that all include at least one HSE), suggesting that organizations sufficient for heat-shock responsiveness are multiple. Although the distal HSEs are more variable than the proximal HSEs, even the latter are not uniform. For example, whereas most promoters in the present study included a proximal (or solo) HSE within 67 bp of the TSS, the proximal HSE is 94–137 bp from the TSS in the *Hsp23* promoters of all species and >270 bp from the TSS in the *Hsp27* promoters of all *melanogaster* subgroup species, anomalies evidently conserved for >40 MY and >10 MY, respectively. In other cases, HSEs come and go during evolution, merge and diverge, move proximally or distally, and expand and contract – but with resultant patterns conserved for millions of years.

### Evolution of HSE features in heat-shock promoters

We cannot unambiguously decipher the relationships of the distal HSEs in *Hsp27* promoters of the subgenus *Drosophila* (Figure 5) species and *D. willistoni*, and so exclude them from the following analysis. While the presence or absence of the remaining HSEs varies, this variation is always consistent with the phylogeny. That is, in no case does a feature disappear in a clade and reappear in a descendant clade. This consistency, combined with the principle of parsimony, allows the unambiguous assignment of evolutionary gains or losses of HSEs to specific periods in cladogenesis (Figures 2–9):

(a) Multiple origins of unique HSEs. In *Hsp23*, three such origins of distal HSEs occurred independently after *D. mojavensis*, *D. willistoni*, and *D. ananassae* each diverged from the other species. In *Hsp26*, two such origins of distal HSEs occurred independently after *D. willistoni*, and *D. virilis* each diverged from the other species. A distal HSE evolved in *DnaJ-1* of *D. grimshawi* after it diverged from the other species. No such events occurred in the other genes examined.

(b) Unique losses of HSEs. A distal HSE disappeared in *Hsp22* of *D. grimshawi* after it diverged from the other species, and is thus absent in this species only. A distal HSE disappeared in the *Hsp83* of a common ancestor of all Sophophora (Figure 9) species examined, and is thus absent in all. This event cannot unambiguously be designated as a loss or a gain from the data for the 13 species, but the *Hsp83* sequence of an outgroup (*Culex*) indicates the HSE was lost in Sophophora rather than gained in a common ancestor of the non-Sophophora species.

(c) Origins of HSEs with subsequent retention in all descendant species. In *Hsp22*, HSE features evolved prior to the divergence of the 13 species, although the distal HSE disappeared after *D. grimshawi* diverged. [An alternative explanation, that the state in *D. grimshawi* was ancestral and a distal HSE of similar size and position originated independently first in a common ancestor of *D. virilis* and *D. mojavensis* and second in a common ancestor of all other species, can be rejected as less parsimonious.] In *Hsp23* and in *Hsp27*, a distal HSE evolved uniquely in a common ancestor of the Sophophora species (but after *D. willistoni* diverged for *Hsp23*), appearing in all descendant species. In *Hsp26*, a pair of distal HSEs evolved uniquely in a common ancestor of the *melanogaster* subgroup species, appearing in all descendant species. A distal HSE similarly evolved in *Hsp27*. In *DnaJ-1* a distal HSE evolved in a common ancestor of *D. persimilis* and *D. pseduoobscura*, and a pair of distal HSEs arose separately in a common ancestor of the *melanogaster* group (Figure 6) species, in each case appearing in all descendant species.

(d) HSEs of *Hsp70* are anomalous in their conservatism (Figure 8). The only routinely multi-copy gene surveyed includes four HSEs of similar size and location in the vast majority of its proximal promoter regions (except in a single copy each in *D. santomea* and *D. willistoni*).

**Figure 10:**
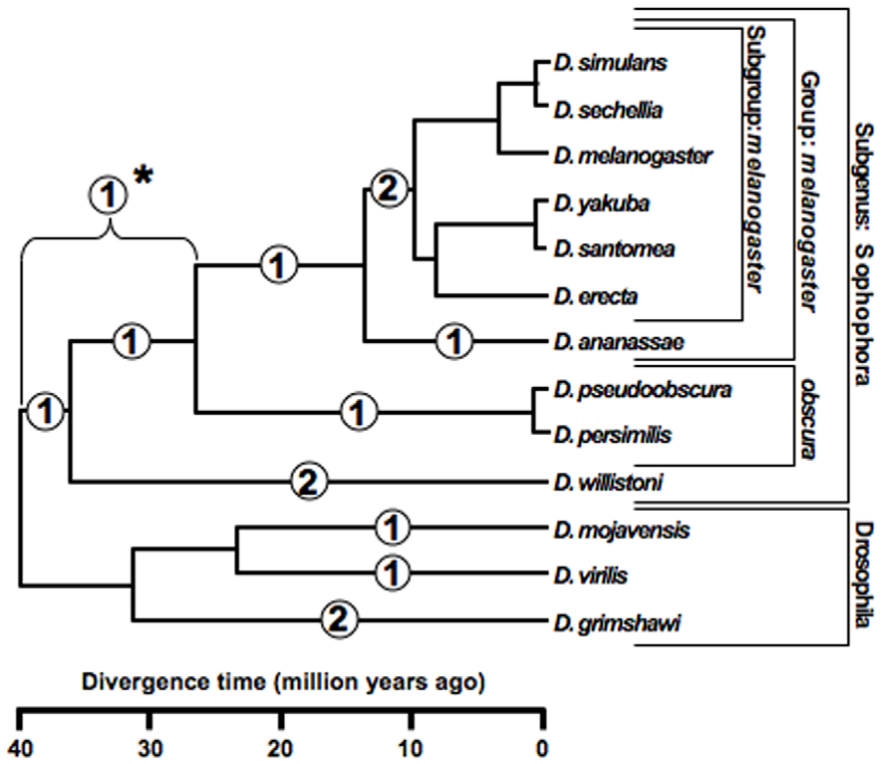
Evolutionary changes in numbers of HSEs superimposed on a phylogenetic tree of the 13 species. Tallies of total changes in all 7 genes surveyed are placed in the centers of the segments in which the changes occurred. The changes themselves, however, may have occurred at any time during the interval the segment represents. *One change, that for *Hsp68*, cannot be assigned to a specific segment because the published genomic sequence for *D. willistoni* lacks a proximal promoter region that can be aligned to those for the other species.

The data support 14 discrete evolutionary events (gains or losses) distributed among 24 branches in the phylogeny, with 0, 1, or 2 events per branch (Figure 10). Lengths of these three classes of branches average 5.2, 19.5, and 29.7 MY respectively; this variation is significant (Kruskal-Wallis test, 2 df, p = 0.0012). HSEs have thus neither appeared nor disappeared in the individual species of the *melanogaster* subgroup or the two species of the *obscura* group (Figures 2–9) presumably because the time since their divergence has been insufficient.

### HSE sequence variation and subunit site composition bias

**Figure 11:**
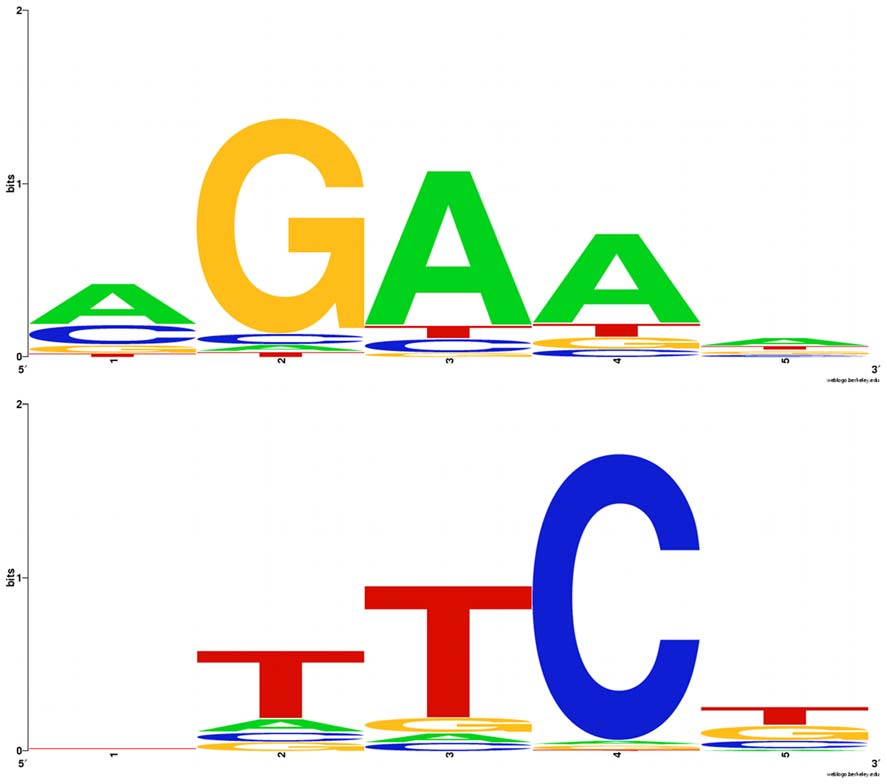
Sequence logos for nGAAn and nTTCn motifs. WebLogos were generated using the entire pool of available motifs, including duplications and multiple *Hsp70* copies.

**Figure 12:**
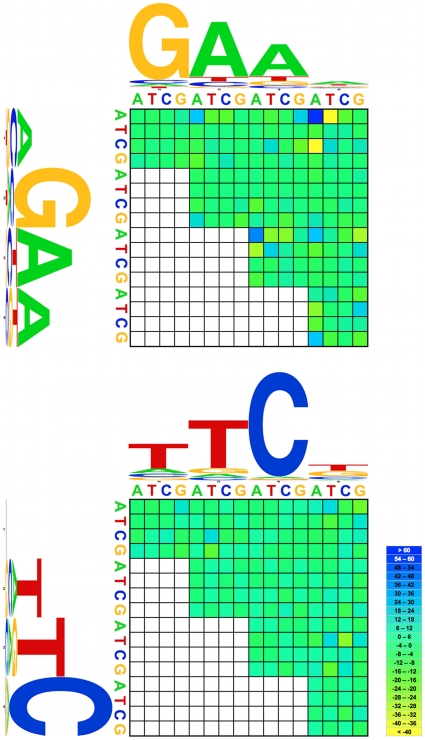
Results of two-way chi-square tests. Subunits were first separated, and tested independently. On the vertical axis, the four largest letters represent the first four positions in the consensus sequence, and the horizontal represent positions 2 through 5. Small letters indicate the pair of bases are tested. Results are summarized as the number of excesses of deficiencies found in each test. Key to heat map: numbers indicated deviation from expected number of observations.

The sequences of HSEs seem far more variable than their number, size, and position. With *Hsp70* HSEs excluded, overall HSEs vary among species (Tamura-Nei gamma distance: mean = 0.410, SE = 0.089; transversions only) as much as do the third positions of codons within the coding sequence of the genes in which they occur. (synonymous: mean = 0.413, S.E = 0.095; HSE: mean = 0.410, SE = 0.089; transversions only). By contrast, certain positions within HSEs are highly constrained. We analyzed 872 nGAAn-type 5 bp subunits and 880 nTTCn-type 5 bp subunits. While our findings (Figures 11 and 12) largely conform to previously reported patterns, some departures are evident. For example, whereas Position 1 in nTTCn has absolutely no nucleotide preference as previously reported, in the corresponding position in nGAAn (position 5) A accounts for 42.7% of the bases. Also, in position 5 in nTTCn only 40.5% of the motifs are T, less than previously reported.

Furthermore, even between otherwise poorly conserved sites in the 5 bp motifs, certain combinations of nucleotides were more prevalent than expected by chance for nGAAn-type motifs (Figure 12). For example, As in both position 1 and position 5 (denoted A1A5) were excessively more frequent than expected from their joint probability, as was A3A4. By contrast, A1T5 and C1A5 were excessively infrequent. Excesses and deficiencies in nTTCn-type motifs were smaller.

The foregoing analysis of 5 bp subunits included those from all HSEs in promoters we examined, including those in all copies of paralogous genes. To assess whether this universal inclusion affects our results, we repeated all analyses with HSEs from a single, arbitrarily chosen gene copy in each species. The results were not appreciably different than those for the more inclusive dataset.

## Discussion

Currently studies of *cis*-regulatory element (CRE) evolution emphasize (a) the discovery of novel CREs through their evolutionary conservation [42], [43], (b) the change in the combination of heterotopic CREs in *cis*-regulatory modules and networks [1], [44], [45], (c) evolved differences in CREs in sister taxa [3], [46]–[50], and (d) the deduction of the general properties of CRE evolution through data-mining on a multigenome-wide scale [7], [51], [52], among others. While these emphases are laudable, they curiously have bypassed the distribution and nature of discrete evolutionary changes in CREs on a phylogenetic scale (e.g., Figures 2–9). [By contrast, comparable investigations of coding sequence (e.g., [53]) and phenotype (e.g., [54], Figure 1 in [50]) are numerous.] Thus, the present study provides a unique glimpse at the evolution of *cis*-regulatory elements (CREs) and the promoters that include them. As foreseen (see Introduction), the combination of a large pre-existing literature on the focal CRE's structure and function, a well-established and detailed phylogeny, and aligned orthologous promoters of the included clades underlie this analysis, and ought to permit similar analyses wherever this combination is available elsewhere. Indeed, [6] have similarly described gains and losses of bicoid and hunchback binding sites, and [3] changes in the structure and function of an enhancer element, in a subset of the 13 species investigated in the present study.

HSEs are always present in the genes we have studied, and presumably the promoters that include them uniformly can respond to activated HSF. By inference, the *Drosophila* heat-shock promoters studied never completely gain or lose HSF responsiveness, but evolve the number of HSFs that can bind, binding affinity, and/or the locations of the HSEs, which putatively affects their ability of HSF-HSE complexes to interact with the transcriptional apparatus. Three studies examining the gain and loss of other CREs in *Drosophila* implicate more rapid turnover [6], [7], [52]; each reports, for example, turnover in the *melanogaster* subgroup species, within which we detect none. By contrast, while HSEs appear or disappear (but never entirely) in evolution, they more frequently expand or contract, merge or separate, and/or migrate towards or away from the TSS. A possible explanation is that the genes hosting CREs in the work of [6], [7], [52] are evolving more rapidly than heat-shock genes. The prior studies examine CREs involved in early development, whose evolution is responsible for the divergent phenotypes of the species. By contrast, the heat-shock response and its major genes are notoriously highly conserved [55], [56] and considerably predate complex eukaryotes [57]–[59]. An approximate counterpart concerns duplicate genes, wherein one member of a duplicate pair can evolve rapidly after duplication. In genes free to evolve in this manner, especially recent duplicates [60], both gene expression and *cis*-regulatory sequence evolve more rapidly than in non-duplicate genes whose evolution is constrained [61]. [*Hsp70*, the one multi-copy gene in the present study, is the only gene with almost no turnover of HSEs; its evolution, however, is constrained by gene conversion, at least among transcribed sequence [62]]. HSEs occur in numerous genes other than heat-shock genes; whether HSEs turn over more rapidly in less-conserved non-heat-shock genes might confirm or reject this explanation.

The phylogenetic context bounds the dates of each evolutionary event. While we have limited our dating to the most conspicuous of evolutionary events, the gain or loss of entire HSEs, changes in size and location of HSEs can similarly be dated. The dating can unambiguously exclude evolutionary explanations. We ourselves have posited that the heat-shock expression system in *Drosophila melanogaster* (and perhaps also *D. simulans* and *D. yakuba*) is an adaptation to the unique thermal environment that this species encounters. While *D. melanogaster's* thermal niche may be unique, the organization of its HSEs is both unremarkable and preceded the evolution of the species. The same is true of the desert species, *D. mojavensis*, and *D. yakuba* vs. its less-thermotolerant sister species, *D. santomea* [40]. With respect to a linkage between regulatory evolution and speciation, none is evident in the heat-shock promoters under study. Finally, the gain or loss of HSEs is evident only for long time periods, as none is detectable in species diverging during the last 10 MY (Figures 2–9).

Nonetheless, our analysis has several significant limitations. Obviously it is only as good as the phylogeny on which it rests. Although the relationships of the 13 species examined in the present study are well established [39], [63], the 12 whose genomes have been sequenced were chosen to achieve objectives [39] other than the elucidation of HSE evolution. Hence, they are less than ideal for present purposes. For example, inclusion of *D. montium*, whose subgroup diverged from the *ananassae* subgroup, would have enabled localization of the evolution of an HSE in *Hsp23* of *D. ananassae* to before or after the divergence. In particular, the time segments pertinent to the evolution of the *Drosophila* subgenus species, *obscura* subgroup species, and *D. willistoni* are too long to time evolutionary events precisely. This limitation can be addressed by selective resequencing of species whose divergence times progressively subdivide lengthy time segments; i.e., phylogenetic walking. *Drosophila* is a speciose group with a detailed phylogenetic tree (http://flybase.org/static_pages/allied-data/phylogeny/Drosophilidae-Tree/list3.html) and representative stocks (http://stockcenter.ucsd.edu) available, features lacking for many other taxa. Another limitation is that sequence variation, especially in non-transcribed sequence, often impedes the alignments on which the analysis depends, thereby reducing sample size and power [6], [7]. We attempted to include members of the *Hsp60* gene family in the analysis, for example, but were thwarted for lack of confidence in their orthology and alignment. In a sister study [64] of 117 primarily single-copy promoters in the 12 sequenced *Drosophila* genomes, despite extensive manual curation only 33% had conservation of the TSS sufficient to be alignable in all 12 species. Lack of conservation may limit the application of our approach to most genes, which are less well conserved than heat-shock genes [55], [56].

As noted, phylogenetic context also discovers aspects that are unique vs. repeatable and constrained vs. variable in evolution, thereby posing functional hypotheses for follow-up. Ordinarily caveats (e.g., chance resemblances among species, mutations in *trans* that compensate for mutations in *cis*) apply to this concept [6], but are minimal in the present study due to the detailed pre-existing mechanistic understanding of regulation of the heat-shock response. The prevalence and conservation of gapped HSEs, tail-to-tail organization, and interactions between positions 1 and 5 in nGAAn-type motifs are among these discoveries. In yeast, HSE variants beginning with nGAAn or nTTCNn (head-to-head or tail-to-tail, respectively) have distinct binding properties and biological activities [26], as tail-to-tail variants can bind more HSF trimers than head-to-head variants of equal size. In *D. melanogaster*, all that is known is that both variants bind HSF with similar affinity *in vitro* [21]. Thus, either the benefit of the tail-to-tail arrangement or some other explanation (e.g., genetic linkage) is yet to be discovered for *Drosophila*. Gapped HSEs have been documented in a number of heat-inducible genes in yeast and are capable of binding HSF [34], but to our knowledge have not been described in *Drosophila*. In the yeast, *Schizosaccharomyces pombe,* the modulation of heat-shock expression through HSF appears to vary based on HSE architecture, suggesting that whether typical or gap-type HSEs occur in a promoter may be related to gene- or stress-specific differences in regulation [65]. Although induction of heat-shock gene expression is not identical in yeast and flies [38], the nature of the HSF-HSE interaction is well-conserved [17], [19]. This fact, together with the conservation of gap-type HSEs across *Drosophila* species, argues for constraint on these motifs, and a possible functional role in *Drosophila*. Finally, the canonical sequence of HSE 5 bp subunits has been inferred largely from a single species, *D. melanogaster*, with inherent ascertainment bias. A phylogenetic and multispecies perspective can test the robustness of a canon based on a single species. Our analysis is consistent with the previously-reported conservation of sites 1 and 2 in the AGAAn motif and site 4 in the nTTCn motif [10], [23], sites intimately involved in the interaction between HSE and the HSF DNA-binding domain [23]. Additionally, the correlations between nucleotide states at pairs of sites (Figure 12) suggests that certain combinations of nucleotides outside the highly conserved regions favor or disfavor HSF binding. This finding suggests that the influence of nucleotide composition at individual sites in a HSE on binding affinity may be non-additive. Interestingly, significant correlations among states at different sites occurred in only the nGAAn-type motif. In conjunction with the finding of a bias towards HSEs with nTTCn motifs at the most distal site (tail-to-tail), this suggests the intriguing possibility that HSF-binding and heat-shock gene expression could differ mechanistically depending on HSE orientation in *Drosophila*.

While our sample of HSEs is both larger and phylogenetically more diverse than previously available, it reaffirms rather than alters the consensus sequence previously derived from more limited samples of HSEs. Importantly, our results overall suggest some caveats on the uses of this consensus sequence. For example, *in silico* searches for HSEs (e.g., [36] should accommodate gapped HSEs and recognize that sites outside the central 3 positions of each 5 bp unit may consequential. Such practices may reconcile the apparent contradiction between experimentally determined binding sites of HSF and predicted binding sites.

While we have posited that pre-existing detailed understanding of a CRE's function ought to facilitate the analysis of its evolution, we may have underestimated the level of detail that is necessary. Although the heat-shock promoter is among the best understood of eukaryotic promoters and piecemeal experimental investigations of numerous variants establish that most are consequential for heat-shock gene expression (see Introduction), a precise functional read-out of evolutionary variation in the heat-shock promoter [as, for example, [66] have attempted at the level of the *cis*-regulatory module] is currently difficult to impossible to obtain experimentally, even with recent advances in throughput [67]. As we have reviewed, the number, arrangement, sequence, and position of HSEs is consequential for heat-shock gene expression. Thus a single 3×5 bp-unit HSE represents 4^15^ potential combinations of nucleotides, as even the highly-conserved positions sometimes vary when a canonical 5 bp-unit is adjacent to it. The HSE may be tail-to-tail or head-to-head. HSEs may be larger than 3×5 bp units, presenting both more combinations and increasing potential for cooperative binding of HSF. Separation between the HSE and the TSS can vary. Multiple HSEs can occur within a single heat-shock promoter, with identical HSEs having different impacts on heat-shock expression depending on their position and arrangement. Finally, mechanisms in addition to HSF binding to HSEs, both in *cis* and in *trans*, regulate heat-shock expression [68]. The specific patterns observed in the 145 promoters of the 13 species presumably are feasible for heat-shock gene expression. Which unobserved patterns are not feasible and which are feasible but have not evolved? Of the observed arrangements, which encode higher vs. lower levels of gene expression (and what levels), and which are functionally equivalent? Elucidation of the *cis*-regulatory code rests on the answers to such questions. The enormous complexity of even such a seemingly simple and well-understood promoter as the heat-shock promoter, however, suggests that this elucidation may be among the “grand challenges” of biology [69]. While “nothing in biology makes sense except in the light of evolution” [70], sometimes even this light is insufficient to make sense.

## Materials and Methods

Sequences for 8 *D. melanogaster* heat-shock genes (*Hsp22*, *Hsp23*, *Hsp26*, *Hsp27*, *DnaJ-1*, *Hsp68*, *Hsp70*, and *Hsp83)* were obtained from FlyBase (http://flybase.org). We screened the highest scoring results of BLASTn and tBLASTn searches with *D. melanogaster* nucleotide coding and protein sequences using sequence homology, synteny, FlyBase orthology assignments, and reciprocal BLAST to *D. melanogaster* to identify orthologs in the other 11 species of *Drosophila* with sequenced genomes.

Several putatively single-copy heat-shock genes had lineage-specific duplicate copies, which underwent further analysis.

Six copies of *Hsp70* are in the *D. melanogaster* genome. We used BLASTn with the coding sequence of *D. melanogaster Hsp70Aa* as a query to each of the other 11 sequenced genomes. All hits with an e-value of 0.0 were further examined to determine whether they constituted a putative copy of *Hsp70*, using a combination of Gbrowse orthology calls, synteny and relative position, and reciprocal BLAST to *D. melanogaster*. *Hsp70* homologs were generally found in high-scoring clusters of >75% identity to D. melanogaster *Hsp70*, often in tail-to-tail configurations. *Hsp68* and *Hsp70* were distinguishable from diagnostic sites [71]. Analyses incorporated all verified copies found in each species [64], except where specifically noted.

The identification of regulatory elements is dependent on the recognition of the promoter region upstream of the TSS. We chose a 1000 bp region that should encompass the majority of proximal promoter regulatory elements [72]. Transcriptional start sites were first determined in *D. melanogaster* using experimental data collected in the Eukaryotic Promoter Database [73] and extrapolated to other species by referencing the whole genome alignments via the UCSC Genome Browser (http://genome.ucsc.edu/). The 1000 bp region upstream of the conserved TSS was then extracted for each gene in each species. In several cases this process was complicated by gene duplication or similarity. First, assessment of TSS location was confounded for *Hsp68*, as several species appear to have copies of *Hsp70* aligned to the *D. melanogaster Hsp68* gene region. We located the UCSC aligned regions in the genomes of species where the alignment appeared incorrect, and confirmed that the predicted coding sequence of the adjacent gene was *Hsp70* through comparisons to diagnostic sites [71]. Local multiz alignments obtained from the PromAn server [74] were exclusively used for the promoter region of *Hsp68* to define the TSS in each species. Second, for several lineage-specific duplications or for the multi-copy gene *Hsp70*, only a single paralog is aligned at UCSC. We used local alignments to determine placement of the TSS in all paralogs. Third, *Hsp22* is duplicate in *D. grimshawi*, but only one copy has identifiable HSEs in the region upstream of the putative TSS and was included in analysis.

We obtained heat-shock promoter sequence from *D. santomea* strain CAR1600 [40] with primers designed from the closely related *D. yakuba* [63]. DNA was extracted from single individuals using a DNeasy kit (Qiagen, Rockville, MD), and PCR was performed using the following conditions: 94°C for 2 min., 35 cycles of 94°C for 30 sec., 50–55°C for 30 sec., and 72°C for 1 min, with a final extension at 72°C for 6 min. Sequence reads were trimmed and edited using Sequencher 4.5.In *D. yakuba*, *Hsp70* occurs in two tail-to-tail clusters. One set of primers enabled sequencing of the entire intergenic region of one cluster (GE24511 and GE26203 in *D. yakuba*); the second cluster (GE26149 and GE24569) proved refractory to PCR amplification. As *D. santomea* is not included in the UCSC genome alignment, we aligned the TSS from this species based on the closely related *D. yakuba* for promoter region delineation.

The defined promoter region upstream of the aligned TSS in all promoters was used as the search region for HSEs. First, we employed a motif finder, part of Mod Tools [75], to locate potential HSEs by searching for the 8-nucleotide sequence TTCNNGAA, allowing for a maximum of 4 mutations and screening for 8 bp sequences with a matrix score of at least 70. This search motif ought to detect all HSEs, which will include this sequence regardless of whether NTTCN or NGAAN occupies the most distal position. HSEs longer than the search motif were obvious as they generated multiple hits in the same region. All HSEs identified in the initial motif search were further screened prior to retention for analysis based on the following criteria derived from experimental data: (1) they must contain at least 3 contiguous alternating 5 bp subunits matching the canonical HSE, (2) they must have no more than 2 substitutions and (3) they must have no mismatch to G in position 2 (NGAAN type) and C in position 4 (NTTCN type), unless the 5 bp mismatched subunit was interior to an HSE that otherwise contained at least 4 subunits. 10 bp gaps were not tolerated under any circumstance.

A final screen was performed to quantify the likely binding strength of each HSE subunit from its sequence similarity with the canonical motif, and to define boundaries of HSEs. We generated WebLogos [76] for each of the alternative 5 bp subunits recovered in the motif search. Bit-scores were summed for each base at the 5 positions in each subunit, and expressed as a percentage of the sum calculated if the preferred base were at every position. Subunits scoring <50% and at an external position in a putative HSE were removed from further analysis. We specifically retained gapped HSEs in the dataset by including subunits scoring <50% but flanked by higher-scoring subunits. Lastly, we used the 12-species genome alignment at UCSC supplemented with multiz alignments of local regions to assess the orthology of individual HSEs. Many HSEs detectable in this search were homologous to previously identified and, in some cases, experimentally confirmed HSEs in *D. melanogaster*, adding confidence to their assignment.

A final screen was performed to test for the possible presence of HSEs consisting of only 2 5 bp subunits [25]. To exclude false positives, we applied a stringent threshold: only motifs conserved across multiple species with a maximum of one substitution (excluding positions G2 and T4) from the canonical HSE motif were retained. This search yielded the final collection of HSEs.

We compared rates of divergence among species in HSE sequence with a neutral proxy, synonymous changes at 3rd positions of adjacent heat-shock coding regions. We performed this calculation among all orthologous HSEs and coding regions for all single copy genes except *Hsp27* to ensure adequate sample sizes. To minimize the issue of substitutional saturation, we based our calculations on transversions only.

We performed 2-way χ2 tests on all possible position pairs for the 5 positions within a subunit, using the entire pool of 1,611 identified motifs. The test was also performed on a corrected pool of motifs, to account for overrepresentation of HSEs from *Hsp70*, and other duplicates. The nGAAn-type motifs were tested separately from the nTTCn-type motifs, as these variants differ in binding ability [26]. The expect values for two given bases in their respective locations was the product of their rate of occurrences at those two positions. Results indicated where excesses and deficiencies arose in the data set. Three-way χ2 tests were not performed due to insufficient sample size.

## Supporting Information

Table S1 Extracted sequences of all HSEs and HSE motifs. All identified HSEs are indicated along with gene and species of origin. Individual motifs that constitute each HSE are also listed.
(0.14 MB XLS)

Table S2 Annotated HSEs in heat-shock promoters. All individual HSEs are indicated within promoters from all species and all genes. HSEs are color coded by distance to the transcription start site.
(0.23 MB DOC)
