# Supplementary material for: Phylogeny Disambiguates the Evolution of Heat-Shock cis-Regulatory Elements in Drosophila
Source: PLoS One. 2010 May 17;5(5):e10669. doi: 10.1371/journal.pone.0010669 (PMC2871787; doi:10.1371/journal.pone.0010669)
Supplement: Table S2 — Annotated HSEs in heat-shock promoters. All individual HSEs are indicated within promoters from all species and all genes. HSEs are color coded by distance to the transcription start site. (0.23 MB DOC) [file pone.0010669.s002.doc]

>hsp22_D_melanogaster

CAAAGGCTCCTGGAATGAATGGGCTCAAAAGGAGGGACTTTGACGATAAACGTCGCTATATTTCTGAATAAATGAAGATTAATATTAATTAATTAATTAATTATTAATAGCTAAAAAAAACAGAAAACTTTAATTATTTGTTAATATTAAGCTGTATTTTTCATATATCTCAAGTTCTTGACTACACCCATGGCAAGCTTATCAATACACACACGTATACACTCGCACTCAGAAAGCTGTGCACTCCCACAAAACTTTCTCTCTCCCACTCTCTAATGGAGCTCTCTCAATCTGTCTCTCTGCGTATGGAAACTGACCTTCCCCAAGGCGCAACAGCGAGAGAGAACTTCGCTAAATGCTAAAATAAAAGGTAAATAAAGTAATATTTGGACACCCAGAGAGCCCCAGAAACTTCCACGGAGTTCGCTAAAGAACAGTGAACAACCCCTAACTAAATGCCATTGCCCGATTTCAGGCAAAGTGGAAAATTGCATCAGCAAAGGGCGAAGAAAATTCGAGAGAGTGCCGGTATTTTCTAGATTATATGGATTTCCTCTCTGCCAAGAGTATAAATAGCCACCGGTTGGACACTGCGCTCTC

AGTTCAAAAAAACCAAACCAACTGCTAACAACTCGAAGAAAGTCAACTAAATTAAAATTT

-204 -175 6

-93 -59 7

>hsp22_D_simulans

ATAGTTTATATCGGATACTTGCTAATTTCAGTGTTGTGATCTACAAAGGCTCCTGGAATGAATGGGCTCAAAAGGAGGGACTTTGACGATAAACGTCGCTATATTTCTGAATAAATGAAGATTTTTATAATAATAGCTGAAAAAAACAGAAAACTTTAATTAATTGTTAATATACTTAAGCATCTACCTAATTTTAATATATCTCAAGTTTATCAATACACATACACCCGCACTCAGAAAGCTGTGCACTCCCACAAAACTTTCTCTCTCCCACTCTCTATTAGAGCTCTCTCAATCTGTCTCTCTGCGTATGAAAACTGACCTTCCCCAAGGCACCACAGCGAGAGAGAACTAAGTGCTAAAATAAAAGGTAAATAAAGTAATATTTGGACACCCAGAGAGCCCCAGAAACTTCCACGGAGTTCTCTAAAAAACAGTGAACAACCCCTAGCTAAATGCCATTGCCCGATTTCAGGCAAAGCGGAAAATTGCATCAGCAAAGGGCGAAGAAAATTCGAGAGAGTGCCGGCATTTTCTAGATTGTAGGAATTTCCTCTCTGCCAAGAGTATAAATAGCCACCGGTTGGACACCGCGCTCTC

AGTTCAAAAAAACCAAGCCAACTGCGAACAACTCGAAAAAAGTCAACTAAACTAAAATTT

-204 -175 6

-93 -59 7

>hsp22_D_sechellia

TGTGATCTACAAAGGCTCCTGGAATGAATGGGCTCAAAAGGAGGGACTTTGACGATAAACGTCGCTATATTTCTGAATAAATGAAGATTTTAATAATAATAACTGAAAAAAACAGAAAACTTTAATTATACTATAATATAGTTAAGAATCTACCCATTTTTCATATATCTCAAGTTTTTGACTACGCCCATGGCAAGCTTATCAGTACACATACACATGCACCTGCACTCAGAAAGCTGTGCACTCCCACAAAACTTTGTCTCTCCCACTCTCTATGGGACCTCTCTCAATCTGTCTCTCTGCGTATGAAAACTGACCTTCCCCACGGCGCCACAGCAAGAGAGAACTTCGCTAAGTGCTAAAATAAAAGGTAAATAAAGTAATATTTGGACACCCAGAGAGCCCCAGAAACTTCCACGGAGTTCGCTAAAAAACAGTGAACAACCCCTAACTAAATGCCATTGCCCGATTTCAGGCAAAGCGGAAAATTGCATCAGCAAAGGGCGAAGAAAATTCGAGAGAGTGCCGGCATTTTCTAGATTATATGTATTTCCTCTCTGCCAAGAGTATAAATAGCCACCGGTTGGACAGCGCGCTCTC

AGTTCAAAAAAACCAAACCAACTGCGAACAACTCGAAAAAAGTCAACTAAACTAAAATTT

-204 -175 6

-93 -59 7

>hsp22_D_yakuba

GTATCTGTAACCATATTTCTAGTGTTGTTGAAATACTGATAAAAATAATAAAATAATAATTGCTGAAAAAAGAACAGTTTTATTAATTGCTATGATCCATAAACATAAATATAAACATAATACATTTCTCATATAGTATGTAAATACATACAATGTATCTGAAGTTTTAAACTACGCCCATGGCAAGCTTATCAACACACACACACAATACAAACACACTCGCACTCAAAAAGCTGGCCACTCTCTCAAACTTTCTCCGCCTCCCACGCTCTTTTGGAGCTCTCACAAGCTTTCTCTCTGCGTACGAAAACTGACCTTCCCCAAGGCGCCAGCGCGAGAGAACTTCGCCAAGTGCTAAATAAAAAGGTAAATAAAGTAATATTTAAATACCCAGAGAGAGAGTCGCAGAAACTTCTACAAAGTTCTCTAAGAAACAGTGGACAACCCCTAACTAAATGCCAATGCCCGATTTCAGGCAAAGCGGAAAATTGCATCAGCAAAGGGCGAAGAAAATTCGAGAGAGTGCCGGCATTTTCTAGATTATATGCATTTCCTCTCTGCCAAGAGTATAAATAGCCACCAGTTGGGCACCGCACTCTT

AGTTCAAAAAAACCAAACAAACAGCTAACAACTCGAAAAAAATCAAATAAACTACAATTT

-204 -175 6

-93 -59 7

>hsp22_D_santomea

ACTGTAGCCAGATTTCTATATAAACGAAGTGTTGTTGTTGAAATACTGATAAAAATAATAAAATAATAATTGCTGAAAAAAGAACAGTTTTACTAATTGCTATGATCCATAAACATTAATATAAACATAATATATTTCTCATATAGTATGTATGTACAATGTATCTGAAGTTTTAAACTACGCCCATGGCAAGCTTATCAACACACATACACAATACAAACACACTCGAAAAAAGCTGGGCACTCTCCCAAACTTTCACCGTCTCCCACTCTCTTTTTTTGAGCTCTCTCAAGCTTTCTCTCTGCGTGCGAAAACTGACCTTCCCCAAGGCGCCAGCGAGAGAGAACTTCGCCAAGTGCTAAATAAAAAGGTAAATAAAGTAATATTTAAATAGCCAGAGAGTCGCAGAAACTTCTACAAAGTTCTCTAAGAAACAGTGGACAACCCCTAACTAAATGCCATTGCCCGATTTCAGGCAAAGCGGAAAATTGCATCAGCAAAGGGCGAAGAAAATTCGAGAGAGTGCCGGCATTTTCTAGATTATATGGATTTCCTCTCTGCCAAGAGTATAAATAGCCACCAGTTGGGCACCGCACTCTT

AGTTCAAAAAAACCAAACAAACAGCTAAACAACTCGA

-204 -175 6

-93 -59 7

>hsp22_D_erecta

TCGGATACGTGTGCATTTTAGTGTGGTGATCTACAAAGGCTCTTGGAATGAATGGGCTCAAAAGGAAGGACTTTGACTGTAACCAGGCCATATGTATTTCTAAATAATAAAATAATAGAAATAATGCTGAAACAATCAAATAACAATAACAATTTATTTCTCATATATCTATGTTCAAATGTATCTAAAGTTTTAAACTACGCCCATGGAAAGCTTATCAATACACATACACAATACACATGCACCCGCACTCAAAAGCTCAGCACTCTCTAAAAATTTCTGTCTCCCACTCTCTTTTGGTGCTCTCTCAAAAACTGACCTTCCCCAAGGCGCCAGCGAGAGAGAACTTCGCTAAGTGCTAAATAAAAAGGTAAATAAAGTAATATTTGGACACCCAGAGAGCCCCAGAAACTTCTACGAAGTTCTCTAAGTAACAGTGAACAACCCCTAACTAAATGCCATTGTCCGATTTCAGGCAAAGCGGAAAATTGCATCAGCAAAGGGCGAAGAAAATTCGAGAGAGTGCCGGCATTTTCTAGATTATATGGATTTCCTCTCTGCCGAGAGTATAAATAGCCACCAGTTGGGCACCGCGCTCTC

AGTTCAAAAAAAACAAACCAACAGCAAACAACTCGAAAAAAGTCAAATAAACTCAAATTT

-204 -175 6

-93 -59 7

>hsp22_D_ananassae

CTGTAAATCAGGAAGACGTGCTGAAAAGGCCGAAAAAATTGCCAAAGCTTTGGGATACACCAAGTGAGATTTTTAGTTAAGAATACTACTAATTCTAATTATATTTTAATTCCAAATATTTTCTTAATTTTTTAGCCTCTCACTTTACGCCGGAGCTTGGCTCGATTGGGCTAAAAAAGAGGACCTATAAATAAAAAATTAAATTTAATTTCTTAACTACACCCATGGTTAAGCTTTGCAATCCAAATACACCCATACACTCGCAAAAAAACCAGCCACTCTCTATTTAATTTTGAATTTCTTACCGCGCATGACCGTAAAAAACTGACCTAACCCAACAAAAGACGAGAGAGAACTCCGTTTATCCGGCAAATAAAACGTAAATAAACTCCGTAGAGAGTTCTGGTGGCATCAGGAAATTTTGAAAGAGCCATATAGCAACCCCTGAGTTAAATGCCATTGCCCGATTTCAGTCAAAGCGGAAAATTGCATCAGAAAAAGGCGAGGAAAATTCGAGAGGCTACGAGAGACCAGAATCGAGAAATTTCTCTCGGTGCTGTCGTTGGGTATAAATAGCAGTCTACGGAGCGCCGGGCGCAC

AATTCAAAACAAAAAGTCCAAACCGACGGATAGCAAGTCGCTAACGAGAAACAAGAAACT

-206 -182 5

-95 -71 5

-66 -52 3

>hsp22_D_pseudoobscura

CTGGAGGCCGAGAAAATTGCCAAGGCCCAGGGTTTTACAAAGTGAGTAGAAATGCATTATTTTTGCCTTACAGCTACTTAACACGGAACTTACATTTTCCCACAGTGTGGTTATTTACAAGGGCTCCTGGCTGGAGTGGGCCCAAAAGGAGGGTCTCTAAATTTATTAGTTAGATTTAAAATTTATGTATAATAAAATTGAATGAAGAAAGTCAAAACAAATTTGAGCTTTTCTACTAACGCCAAAGCTTAGCCATACATGCAAACACACGAGCACACACATGCACTTTAGCAACGCTAGTCGTCTCGCTTTCACTATAGCTCTCTTCAATTGCTCTCTCTGTCGAAAGCACTGACACTGACCTACCGCAAGAAGCAACCAAAATGAGAGAGAGAGATATACAAGCAAATAAACCGAAAGCTGTCTATGGAGCGCTCTAGAGACATCTCCCTGTTGCTAGAAGCCACCGTGCCACCACCAACCACCACCACATTGACCGCTCAAAATTGCATCAGACGTAGTACGCCAAGAAAGTTCCGGATGTTTCTATACACTAGCGGCGGCACGTATAAATAGCTGGCACAGAGGGCCGGCACTCGC

AATTCAACAAAAGCCCAGCAAGATAGCACCTCGCATACTGAGAGAGCAAGCAAGAGAAAT

-172 -153 4

-77 -53 5

>hsp22_D_persimilis

GGCCGAGAAAATTGCCAAGGCTCAGGGTTTTACAAAGTGAGTAGAAATGCATTATTTTTGCCTTACAGCTACTTAACACGGAACTTACATTTTCCCACAGTGTGGTTATTTACAAGGGCTCCTGGCTAGAGTGGGCCCAAAAGGAGGGTCTCTAAATTTATTAGTTAGATTTTTAAATTTATGTGCAATAAAATTGAATGAAGAAAGTCAAAACAAATTTGAGCTTTTCTACTAACGCCAAAGCTTAGCCATACATGCAAACACACGAGCACACACATGCACTTTAGCAACGCTAGTCGTCTCGCTTTCTCTATGGCTCTCTTCCATTGCTCTCTCTGTCGAAAGCACTGACACTGACCTACCGCAAGAAGCAACCAAAATGAGAGAGAGAGAGATATGTACAAGCAAATAAACCGAAAGCTGTCTATGGAGTGCTCTAGAGACATCTCCCTGTTGCTAGAAGCCACCGTGCCACCACCAACCACCACCACATTGACCGCTCAAAATTGCATCAGACGTAGTACGCCAAGAAAGTTCCGGATGTTTCTATACACTAGCGGCGGCACGTATAAATAGCTGGCACAGAGGGCCGGCACTCGC

AATTCAACAAAAGCCCAGCAAGATAGCACCTCGCATACTGAGAGAGCAAGCAAGAGAAAT

-172 -153 4

-77 -53 5

>hsp22_D_willistoni

AGAATTTATATAAGTTTAAATTTGAATTTAATTTATACATAAGTCCGAACATTTATTTAGCACTCATGTTTATGATGGTTCCTGGAACGATTGGGCTAAAAGAGAGGGTCTTGCTTAGAGCGAAAGGGCAGCACAATAAATAAGTTTTGGAAAGTTTTTAAATTTATGGAACCAGCCATCAATGGTATAAATACGTACTATCCTTATTTTATTTGGAATAAATTCTACTCTAAATTGTAAATATTTTTATACCCTTGCAAAAGTCATAGCCTTTTGGTTTTCACATCGCTAGTTTAACACACAGACAACAAACACATGAACACTGACCTGCCTCAACACGAAGAGAGAAAGCGCAGAGAGAGTAAGGAAACTTACGTTCTCGGAGCTTCTGTTAAAGCAGTGCAGCCCTTATTAGTATGGATATACATACGTATGTACACACATATATGTAGATGTATGCATAAAAATGTCACTGGCCAATTTCAAACGTTTTATAAAATTGCATCAGAATTTCGAGAGAGAGCTAGATACTTCTAGAGACGACACTTGCTGGGAATGTGGTGGGTATAAATAACAGCCGGCACTGCTAGCATGCTTCAC

AGTTGAAACTTATCAAATTTGGCGAAAGCATCGCTCTCCGGCAGTCCCTTTCTGGAAGTG

-225 -211 3

-90 -61 6

>hsp22_D_mojavensis

CAAGTTTAACACGCACGATGAAATTGGTTCATACAAAAATATATAAATGCTGTAGATAATTGGGAGAGGGGGTAGAAGCTACGCCTATAATATAATTATTATTTTCACAAGATAAAATCTAATCTTTTTCACGCGCAGGTTATATCATAATTATATTAAAAAGCTGACCCCTTTACTACGCCCATGCCCATACCTCACACACACACACATGAACGCAAGCTCATTTTGCTGAGAAGACACAGCAATGTATACTCTCAATAAACCACTCTCACTACCCACCCTCTCTCTCTCTCTCTCTCTCGTTCTCTTCTATCTCTTATTTTTTGAACTGACCTACCACACTGGTGTTTGTAAATAAAAACTGCTTCGCTTTGTTAAATTTCTTGAAACTTCCATTGTTGAGCAACCATTAAGAGAAATGAAAAGTATTTTTACTTTAAATGCAATAATAGCATGCCCTCAACAAACACTATCTATGTAAATGCCAGCCGTATAAGAATATTCAAAATTGCATCAGCTGCCTTGTCGGGAGAGTGCGAGAACATTCTCGAATGCGTTGACATTTCCTATAAAAGGCAGCCGGCATTTTCTGCTCTCAAC

ATTTGAACAAAGTACGTCAAAGTGAAAAGAACTGCCGATAAGCAGTAGCAAGCTAAAGAG

-221 -207 3

-77 -48 6

>hsp22_D_virilis

TAACCCATGTTTATGTTTGTGTTTAACACCCCCTTTCAGTTTTGATCAATTTTTTCTAGAGCAATATTTTGTATGTGTGAATTCGTTAAACAAAGCTCATGCGAAATATGTTTAAAATAAAAATTACAGCTAAGTAAATACTCAAGCTTAAGCAAATTTGTGCGCACCCTCACACTACGCCCATGTCTTATATGTATATGTGCCTCGCCCTCTCTCTTTCTCTCACACGCACACGCACGCACTCGCGAGCTTATTTACGGAACACACACACACATATTCTCTTTTTTTATCTGCCATTTGCTGCTCTCTTTTGCGTTACCAAACTGACCTGCCTTACATTTTTTGTAAATGCCGCAAAACTGCGTCGCATTAGAAAAAGTGCTTAAGAACAATCGAAAGTTTGATTGATGTACGTGAAATTAATCGAAAATATATTTACTTAAAAAGTTCACAAGCTGAACGCTGCTTAAATGCCAAAAAGGTAAAATAATTCTAGAAGCCCAACAAATTGCATCAGCTGCCAAGTCGAGAGAGCGCCGGAACGTTCTCGAGCGCAACGCCGTTTCGTATAAATAGCAGCCGGCGTCGCGCTCTCTCCAC

AGTTGAATCAAATCAGTCAAAGCGAACGGAGCTCGCGTGAAGCAGCAAGAAGAAGCAAGA

-191 -172 4

-77 -48 6

>hsp22_D_grimshawi

TTAGCACAAAGAATCAACAATGAAAAACAACTGCAAATTAAATTTCGATTCGCAATCAGAGTCAAACTGCGTTAGGTTTATAATGTGCATGCGTCTGATTAAGGTTATCATTGTTGCTGTTGCTATTGCTTTTTAATCTCGCCAATAAATGCATGAAGAGAGAATAAATTTAATTCACTCAAATTTGATTTTAATCTCGAACCAATAGCAACTAAGAAGCAAATTGAACAGCCCGCGCAAATATACCCTATTAGCAGTATTTGAATGTTATAAGTTTTAATGCCGATGAAACTCATTATATTTTATTTTATTATTACACTTTCATTGGAGGTTTGTTTTTTACCAACCAAAGTACCTCAGTTGGTACATTTGAACCACATTCGTCAGCCGTGGTGACATTACTGACCCGCCTTAACGAAATGTGAATTTATTAACATACAAGCTTAAATTCACCACAATATGTTGTGGAACTGTCGGAATTATGTTAACGTTATGTTTAGCGGTCGAGAGAGTGCCGGAAAGTTCTACAAAGTTCAAACTTAAATTCACCATTAATAAACCTAAAATGACGAACAGCTGCCCGAATTTATTAATGTACAA

ACTTAAAGTCACCATAAATAAACAAAAATCAATAAAATGCTGTGCAGTTGCCCTAACATT

-99 -70 6

>hsp23_D_melanogaster

TCATTTTTACATTTTCTTACTGATTGGAGTAATAATACGCTTACATCCCATGGGTTATTACCTACCCGGCATTAACCACTTATGTATTTTAATTAGATTTCCCCACTACAGAGCCCCATTCTTGGATATTAATTAAAGTTAATAGCTTAAATGCCAGGCCATAAAAAGAAGAACTGTTCTGCAGTCTCGAAGTTTCGCGAATTTACTCCATCCTTCGTGGAATATACTCCAACCTTCCTATCTGCTATGTATGTACATACATACGTGCTTACATACGTACATCTATACATACACATAATATTTGCCGGTGCTGATGCGACTTATCACTCCACCAGGCCTTTTCATTCCCACTCCCCTAGGAGATTGCTCATTTTCCATAGCGATACTCTCACTTTCAATGGCAGATAATGCGTAATTGCGGCAAATTCGAGAACTCTGCGATATTTTCAGCCCGAGAAGTTTCGTGTCCCTTCTCGATGTCGATGTTTGTGCCCCCTAGCACACAGACACGACGCGCACACACACAGCGCCGACGGGCGCACGCACACTACGATAGCGAGCGGTTGTATAAATAACCGGCACTTTCGTGCAACCGGCGTC

AGTTGAATTCAAAAAGCCAAAGCGATAACAGCTAAAGCGAAAGTAACCTATCAACAAAAG

-418 -384 7

-382 -363 4

-181 -152 6

-151 -122 6

>hsp23_D_simulans

CTTCATTGATCCAAATCGTATCATTTTTACATTTTCTTACTGATTGGAGTAATAATAGGCTTACATCCCAGGGGTTCTTAAATATCCGCCATTAACCACTTATGTCTTTTAATTAGATTTTCCCACTACAGATCCCCCTTCTCGTGAATTAATTAAAGTTAATAGCTTAAACGCCAGGCCATAAAAAGAAGAACTGTTCTGCAGTCTCGAAGTTTCGCGAATTTTCTCCATCCTTCGTTGAATGTACTCCAACCTTCCTATCTGCTATGTATGTACATACATACGTACATACACATAATATTTGCCGGTGCTGATGCGACTTATCACTCCGCCAGGCCTTTTCATTCCCACTCCCCTGGCAGATTGCTCATTTTCCATAGCGATACTCTCACTTTCAATGGCAGATAATGCGTAATTGCCGCAAATTCGAGAACTCTGCGATATTTTCAGCCCGAGAAGTTTCGTGTCCCTTCTCGATGTCGATGTTTGTGCCCCATTGCACACAGACACGACGCGCACACACACAGCGCCGTCGGGCGCACACACACAACGACAGCGAGCGGTTGTATAAATAACCGGCACTTTCGTGCAGCCGGCGTC

AGTTGAATTCAAAAAGCCAAAGCGATAACAGCTAAAGCGAAAGTAACCTATCAACAAAAG

-398 -364 7

-362 -343 4

-181 -152 6

-151 -122 6

>hsp23_D_sechellia

CTTCATTGATCTAAATCGTATCAATTTTACATTTTCTTACTGATTGGAGTAATAATAGGCTCACATCCCATGGGTTCTTAAATATCCGCCATTAACCACTTATGTATTTTAATTAGATTTTCCCACTACAGATCCCCATTCTCGTGAATTAATTAAAGTTAATAGCTTAAACGCCAGGCCATAAAAAGAAGAACTGTTCTGTAGTCTCGAAGTTTCGAGAATTTTCTCCATCCTTCGTTGAATGTACTCCAACCTTCCTATCTGCTATGTATGTACATACATACGTACATACACATAATATTTGCCGGTGCTGATGCGACTTATCACTCCGCCAGGCCTTTTCATTCCCACTCCCCTGGCAGATTGCTCATTTTCCATAGCGATACTCTCACTTTCAATGGCAGATAATGCGTAATTGCGGCAAATTCGAGAACTCTGCGATATTTTCAGCCCGAGAAGTTTCGTGTCCCTTCTCGATGTCGATGTTTGTGCCCCATTGCACACAGACACGACGCGCACACACACAGTGCCGTCGGGCGCACGCACACTACGACAGCGAGCGGTTGTATAAATAACCGGCACTTTCGCGCAGCCGGCGTC

AGTTGAATTCAAAAAGCCAAAGCGATAACAGCTAAAGCGAAAGTAACCTATCAACAAAAG

-398 -364 7

-362 -343 4

-181 -152 6

-151 -122 6

>hsp23_D_yakuba

CTATATACATCTGTACATACATGGGCTATAAATCTTATTATTTTTATTATTTCTTACAGATGAGCATTTACAGACTTACAGCCCATGGGTTATTACCTATCCGTCATTAACCATTTATGCAATTTAATTATTTCCCCCACTGTAGACCCCCTTTTTTGGATATTAACTAAAGTTAATAGCTTAAATGCCAGGCCATAAAAAGTAGAATTGTTCTGCAGTCTCGAAGTTTCGCGAAAGTGCTCCATCCTCCGAGGAATGTACTCCAACCTTCCTATCTCGTATATATGCTTGTATGTAGACAAAATATTTGCCTGTGCTGATGCGACTTATCACTCCACCAGGCCTTTTCATCCCCACTCCCCCAGCAGATTGCTCATTTTCCATAGCGATACTCTCACTTTCAATGGCAGATAATGCGTAATGCCGGCAAATTCGAGAACTCTGCGATATTTTCAGCCCGAGAAGTTTCGTGTCCCTTCTCGATGTTTGTGCCCCATTGCACACAGACACGGCGCACACACACACAGCGATGGCCGTCGCACCCATACAACGACAGCCAGCGGTTGTATAAATAGCCGGCACTTTCGTGCAACCGGCGTC

AGTTGAATTCAAAAAGCCAAAGCGATAACAGCTAAAGCGAAAGTAAACTTTCAACAAAAG

-384 -350 7

-348 -329 4

-180 -146 7

-145 -116 6

>hsp23_D_santomea

ATACACATCTGTACATACATGGGCTATAAATTTTATTATTTTTTTTATTTCTTACAGATGAGCATTAACAGACTTACAGCCCATGGGTTATTACCTATCCGTCATTAACCATTCATGCAATTTAATAATTTCCCCCACTGTAGACCCCCTTTCTTGGATATTAATTAAAGTTAATAGCTTAAATGCCAGGCCATAAAGAGCAGAAATGTTCTGCAGTCTCGAAGTTTCGCGAAAGTGCTCCATCCTCCGAGGAATGTACTCCAACCTTCCTATCTCGTATATATGTATGTATGTAGACATAATATTTGCCTGTGCTGATGCGACTTATCACTCCACCAGGCCTTTTCATCCCCACTCCCCCAGCAGATTGCTCATTTTCCATAGCGATACTCTCACTTTCAATGGCAGATAATGCGTAATGCCGGCAAATTCGAGAACTCTGCGATATTTTCAGCCCGAGAAGTTTCGTGTCCCTTCTCGATGTTTGTACCCCATTGCACACAGACACGGCGCGCACACACACACAGCGACGGCAGTCGCACCCATACAACGACAGCGGGCGGTTGTATAAATAACCGGCACTTTCGTGCAACCGGCGNN

-386 -352 7

-350 -331 4

-182 -148 7

-147 -118 6

>hsp23_D_erecta

GCATTATACATATATTGTATTACAAAACATTCATGAACAAAAACACATCCTTTAAATCCTTCTATGTTTGTACATTCTCTACATTCTTACTGATGAGGGTAATAATTGACATACAGCCCAGACTTAGTAAACCATTAACCATTTATGTAGTTTAATTAGATTCCCCCAATCTAGACCCCCTTTCTGGGATATTAATTAAAGTTAATAGCTTAAATGCCAGGCCATAAAAAGAAGGGAAGAACTGTTCTGCAGTCTCGAAGTTTCGCGAAAGTACTCCATCCTCCGGGGAATGTACTCCAACCTTCCTATCTGGTATATACACATAATATTTGCCTGTGCTGATGCGACTTATCACTCCACCAGGCCTTTTCATACCCACTCCCCCAGCAGATTGCTCATTTTCCATAGCGATACTCTCACTTTCAATGGCAGATAATGCGTAATTCCGGCAAATTCGAGAACTCTGTGATATTTTCAGCCCGAGAAGTTTCGTGCCCCTTCTCGATGTTTGTGCCCCACACACACAGCGACGACCGTCGCACCCATACAACGACAGCGGGTGGTTGTATAAATAGCCGGCACTTTCGTGCAACCGGCGTC

AGTTGAATTCAAAAAGCCAAAGCGATAACAGCTAAGCGAAAGTAAGCTATCTGCAAAGGA

-350 -316 7

-314 -295 4

-158 -124 7

-123 -94 6

>hsp23_D_ananassae

AAAAACACTATATATAAACTCGTATCAACTCATCAAATAATCTAGAATATTCTGGAAATTTCTCTTACCTCTCTACATAAAAGATTTTCCAATTACTCACTACAAAATTTTTAAACTCAACCGTACATCAATTTTCATATATTCTCTCCTATTTTTTGACATTTTAATTAACATTTAATGAAAGAAAACCCGTCATAAAATAAGGAATTAAGTGCAGAATATTCGCGAATAATCTCCATCCTTCGGGGTCGGAGTTCAGCAGCTGTCCCGTCCTATCTGGCAAATACATAAACATAATATTTTTACGCTGATGAGGCGACTTATCACTCCACCAGGCCTATAGCCACTCCCGGCTACCCTCCAGCCATCCGCAATCATTGCTTTCCCCTAGAAATACTCTCGCTTTCAATGGCAGATAATGCGTAATATCTGCAAATTCGAGAATGTTGCGATATTTTCAGACCGAGAAATTTCGTGGCCCTTCTCGGTGCTGTGCCTCTTTGCCCAGACACACCAACAGATACAGATACGCCGTAGCTCACAGATACAGATGCACGGCGAGCGAGTATAAATAGCGGGCGCTTGCGTTCCACCGGCATC

AGTTGAATTCAACAAGCAGCTCTAAACGCTACCAACCAAGCAACCACGAAACCAAGCTAT

-562 -538 5

-385 -356 6

-347 -333 3

-170 -141 6

-140 -111 6

>hsp23_D_pseudoobscura

ATGCTTAAATTTGTACAGACTATTCGTATTCCCTGTTGTTCTTAGCTTCTCCACTCTATCCCTATTTACTACGGCTCTCCCTGTATCTGTTCTCTATTCCGTATCTGTCTGTCTGATTATATGTATTCCTAGGTGAAAAAATAGACATCGAGAAAATTCTAGATATTTCCTTTAAAATTGCCAATTAAATCTGGCAAATCGAATTGTCTTTTTCTTAGTCTTTCTTAATTAATTTGCACCGAGTGCTTCCAGAATAATCCAGAACCTTTGCCTTTCGTGGTATTTTCCCTCACTTTTCATCTTTTATGGGCTGTACATAATATTTGGCCGTGCCTCTGATGATGGGACTTGTTATTCCACATGGCTCGCCCCGCCGCTCATTCCTTATCATTTTCCCGGCGATACTCTCGCTTTCATTGGGCAGATAATGCGTAATTTCTGCGATTCTGCCAGAAAGAGATAGAAGCTTCGTGCAGTTTCTAGATGTTGCCACGCCGCACACAGACAAAACACACACGCAAACTGCCCTCGAAGTAGAGCTGCCACCACCCAGCTGTCGTCAGAGTATAAATAGCGGGCGCTTTCGTTCGGACCGGCATC

AGTTGAATTCAAACCGTCAAACCGTTAAAGCGTCAGCCAAAAAAGCTTTCTCTCAGAGAA

-455 -431 5

-355 -336 4

-170 -161 2

-159 -110 10

>hsp23_D_persimilis_A

ACAAATCTAATATACATGCATACATATGTATGTATGTACATATTTACCCCTATACTTATTTTGAGTATCGGCTATAAAAATAGCCATCGAGAAAATTCTAGATATTTCCTTTAAAATTGGTCAGGACTTGCGGAAAAATTATGGAAATAAAATCTGGCCAATTTAATTGTCTTTTTCTTAGTCATCAACTAAAGTTACTGCTAAAACGCTGAAGAATGAAACTGTTAATTAAATTGCACAGAGTGCTTCCAGAATAATCCAGAACCTTTGCCTTTCGTGGTATTTTCACTCACTTTTCATCCTTTATGGGCTGTACATAATATTTGGCCGTGCCTCTGATGATGGGACTTGTAATTCCACATGGCTCGCCCTGCCGCTCATTCCTTATCATTTTCCCAGCGATACTCTCGCTTTCATTGGGCAGATAATGCGTAATTTCTGCGATTCTGCCAGAAAGAGATAGAAGCTTCGTGCAGTTTCTAGATGTTGCCACGCCGCACACAGACAAAACACACACGCAAACTGCCCTCGAAGTAGAGCTGCCACCACCCAGCTGTCGTCAGAGTATAAATAGCGGGCGCTTTCGTTCGGACCNNNNNN

-516 -492 5

-355 -336 4

-170 -161 2

-159 -110 10

>hsp23_D_persimilis_B

TATGCTTAAATTTGTACAGACTATTCGTATTCCCTGTTGTTCTTAGCTTCTCCACTCTATCCCTATTTACTACGGCTCTTCCTGTATCTGTTCTCTATTCCGTATTGTCTGTCTGATTATATGTATTCCTAGGTGAAAAAATAGACATCGAAAAAATTCTAGATATTTCCTTTAAAATTGCCAATTAAATCTGGCAAATTGAATTGTCTCTTTCTTAGTCTTTCTTAATTAAATTGCACCGAGTGATTCCAGAATAATCCAGAACCTTTGCCTTTCGTGGTATTTTCCCTTACTTTTCATCCTTTATGGGCTGTACATAATATTTGGCCGTGCCTCTGATGATGGGACTTGTTATTCCACATGGCTCGCCCTGCCGCTCATTCCTTATCATTTTCCCAGCGATACTCTCGCTTTCATTGGGCAGATAATGCGTAATTTCTGCGATTCTGCCAGAAAGAGATAGAAGCTTCGTGCAGTTTCTAGATGTTGCCACGCCGCACACAGACAAAACACACACGCAAACTGCCCTCGAAGTAGAGCTGCCACCACCCAGCTGTCGTCAGAGTATAAATAGCGGGCGCTTTCGTTCGGACCGGCATC

AGTTGAATTCAAACCGTCAAACCGTTAAAGCGTCAGCCAAAAAAGCTTTCTCTCAGAGAA

-455 -431 5

-355 -336 4

-170 -161 2

-159 -110 10

>hsp23_D_willistoni

TTATATTTATATACACATACATATGTATGTATGTATGAACATACATACATATATGTACATGTGTATATACATACATACATATACTAGAAGAATACTTTCGATCCTCATATATATGTACATATGTATGTATGAACTCATGTAGGTTCATATGTACATATGTATGCATTATTGTTAATTGCAGCTTATCTTATCACAACTGTCATCCGCTCTTCATTTCTTTAACCTCATCTCACTTTCATTTTCTGATAAAGTGTAATTTTCTGCAATTTCTAGAATAAAATTGCGAACTGTCTTATTTATTCCACACACACATATGTAAATAGGTACATGTATACATACATACATACATACATACATAAGTACATACATATGCATGGGCTAAAGTATCTATGGATATTCAACAAAACATCTCTGTGCTGTTTCCCTTTATTTCAAGTTTTGACTACGCATGTGACGACAATTCTGAGAGAGAACGATGATACCAGATTGTACGCGAATATACCAGAACATGCCCCAAACACACATACATCCACAGCAAATTGTTGCAACCACCGAATCAGCCACCCTATAAATAGCCGGAGTAGCTTCTGGCCCAGCGTC

AGTCACTTACTAAAAACCAGAGCGTTAAGACGCCACTGCAAAAGAAAGTAACAGTAAATT

-166 -137 6

>hsp23_D_mojavensis

ATTTACGTACATACAAACATACATACATACGTTGAGTATGGCAAAATTGCCATTAATAAATTCCGCTGTTAACAAATGAAAATAAAGTCAGTGAAATATTAATAAATAAATTTAAAAGAGCAAATGAAAATGTCTATAGCTATAAATTAACAAATTTTCTAGATATGTACATATGTACATTATCTGGAAAGTTACAGAACATTCTTCTATCTGCAATGTTTTTGTTTGTTTATTCTCTCTGTTATCACTTTTCTCGCTCTTTAACTGCTGCTGTTGCTATCTCAGCGAAACACTCTCACTTTCGTTGCTAGATAATGCTGCAAATGAATATAAATTTTATATACTTACTTACATATAAATGTATGTATTCTTGTATATACATATCTACAAGCATCACGTACATACACACATTCGAGCGCTTTCCAGAACATCGGCAAAATTATTCAACTTTAGCCAGAGCCAGAAGTTTCCTTGGCGTGCCCAGCTCTGCTATCTCTCTCGCACGCACAAGCGCGCGCGTAACGGAGTCACCCGGCAGGTTAATCGGCAAATTCACCACCGGGCAGCTATAAATACGCGACGCATTTCGTTCCCGTCATC

AGTTTACTTCGAAAAAGCAAAGCAACAAGCAACAAACAAAAAGTCAACACAAAGCCTTCC

-420 -396 5

-191 -172 4

-149 -130 4

>hsp23_D_virilis

ATTGATACATATGTACATATGTATACACTTACGCATGCATACATGTACAAGTACAAGTACATGTAGAAGTGTAGAAAGTAATCGTTATCATGGAAATGCAGAAATTGAGTGGGATGTAGCTGTATGCACACACTGTGTGTATATATGTATGTATGCACATACACATGCCCCTTCAATAATGTGTACAAATGTACGTACATATACTATATTTTGTTTTTTTATATTTTATTTGCACTGCCAAAAAACAGCCAGCAATTTTGTCTATAACATGACATGTATGGTGTGTGTATAGATACATGTACATGCATACATATGTACATATACTTTATGAAAAAGCATCAGCGAAACACTCTCACTTTCAGCGCTAGATAATGTTGCGTTGCGTTTAAATGCATAAATATGTGTGTATATATGTATGGGCATATGTATGTATTCGAGCAATTTCCAGAACATTTCAGAAATTATTCAACACTAGCCAGAGCCAGAAGTTTCGTAGGCTTTCCGCGACGCGCCGTCTCCCTCATTCGCACAGACGCGCCCGCAAGCAAAAGCCACCACCCGAGCAGCTATAAATAGCCGGCTTGCTGCGTAGTCGGCGTC

AGTTTGATTCGAAAAACCGAAGCAATAAGAAATATCTACACAAAGTCTCCAAACGAAGTA

-169 -140 6

-132 -108 5

>hsp23_D_grimshawi

ATAAATTACATAGATACATAGATATATACGCAAAGAACGTGTGTAAATATTTATGCATGCTTGTAAATAAATATTCATTATCACTTTGTATTTTTTACCTTATAGTTTTTATTTATTAGCACCACTGAATTAAATTTGACATTATATCGTGCAGTATCAGTAAAATATCAATCTAAGCCAGCAATTTTTAGTCGATTTAATAAAGCTGATGTGGAATATTCATTAAGAAGAGGAATCCGGAACATGGTTTAAAATATCCTCGCTTTTGCTCTTACTTTTGAGCTTGTTGTTGTGTGTTTGTTGCTCTCATTAGCTGCTCTCCAACATCCATTTGAGCGGCAAACATTTGTTGTCTTATCGGCAGACTCTCACTTTCATTTATAGATAACGTTGCGTATATATTCATGTGTGTGTGTGTGTCTGTGTGTATTTACTTATGCGGAAATACATTTTTTCGAGCGATTTCGAGAACATTTACAGAGCTAGAACTTTCGGCAAACATTGTGTCGTTGTCAGCCATCTCGCTCTGTCGCACAGAGTCGTGTGAAAATCCACCACTCAAGCAGCTATAAATAGCTGGAGTATTGCGTCGTTGGCATC

AGTTCGATTCGAATAACCGACGCAATAACAAATCGTCAGTCTGTCCACTACACAGCAAGT

-148 -129 4

-121 -107 3

>hsp26_D_melanogaster

AGCGAGAAAGCTGACGGGAAAAGCACTCAATTACTAATAGTGGGAGATTGCTGGCGTTATATGTATGTATGATTTTCTAAAAAACATATGTGACAACAACTACAAGTATTCCAGTAAAACTTAAAGACAGAAACACGAAATAATGTACTTAATAAAGAGGAAAACCAGAATAAAAAAAACTGACGTTTTGTTTTGTTTGCCGTTAGCCGGCTGTTTCTTTTGCGCTCTTTCTAGAAAATTGCAACAACTCTCTAGAAACTTCGGCTCTCTCACTCATACAGGCGCACTAGCTCTGCTTTTGCGCGTACGACAACAACTACATTTAAAATTTCTCGAAACTCATGGCATTTATTGGGAAAGGTTAGTTAGTTTTATTTTTTGTTTTTAGAGCAGCATTCAATTTAGACTTTTATAAAAGAAATTTCTAATTTGATCCCTCGTTTATCAAACGATACAAAGCTATATTCATAATTTTTTCTCTCTGTGCACGTTCTCTCTCTTCTCTTCTCTCTCTCTACTCTTTCCTTTTTCTGTCACTTTCCGGACTCTTCTAGAAAAGCTCCAGCGGGTATAAAAGCAGCGTCGCTTGACGAACAGAGC

ACAGATCGAATTCAAAAATCGAGCAGTGAACAACTCAAAGCAACTTTGCGCAAAAGCAAA

-373 -364 2

-352 -338 3

-73 -44 6

>hsp26_D_simulans

GAAGCGAGAAAGCTGACGGGAAAAGCACTCAATTACTAAAAGTGGGAGATTGCGGGCGTAATATGTATGTATGATTTCCTAAAAACATATGTGACAACAACTACAAGTATTCCAGTAAAACTTAAAGACAGAAACACGAAATAATGTACTTAATAAAGAGGAAAACCAGAATAAAAAAAAAAATGACGTTTTGTTTTGTTTGCCGTTAGCCGGCTGTTTCTTTTGCGCTCTTTCTAGAAAATTGCAACAACTCTCTAGAAACTTCGGCTCTCTCACGCATACAGGCGCACTGCCTCTATTTTTGCGCGTAAGACAACAACTACATTTTACAATTTCCCGAAACTCACGGCATTTATTGGGTAAGGTTAGTTAGTTTTATTTTTTGTTTTTAGAGCAACATTTAATTAAGACTTTTATAAAAGACATTTCTAACTTGATCCCTCGTTTATCAAACGATACAAAGCTATATTCATCATTTTTTTCTCTCTGTGCACGTTCTCTCTCTTCTCGTCTCTCTCTCTTTTCTTTTTCTGTCACTTTCCGGACTCTTCTAGAAAAGCTCCAGCGGGTATAAAAGCAGCGTCGCTTGGCGAACAGAGC

ACAGATCGAATTCAAAAATCGAGCAGTGAACAAGTCGGAGCAACTTTGCGCTAAAGCAAA

-370 -361 2

-349 -335 3

-73 -44 6

>hsp26_D_sechellia

CTGACGGGAAAAGCACTCAATTACTAAAAGTGGGAGATTGCGGGCGTAATATGTATGTATGATTTCCTAAAAACATATGTGACAACAACTACAAGTATTCCAGTAAAACTTAAATACAGAAACACGAAATAATGTACTTAATAAAGAGGAAAACCAGAATAAAAAAAAAACTGACGTTTTGTTTGCCGTTAGCCGGCTGTTTCTTTTGCGCTCTTTCTAGAAAATTGCAACAACTCTCTAAAAACTTCGGCTCTCTCACGCATACAGGCGCACTGCCTCTATTTTTGCGCGTAAGACAACAACTACATTTTACAATTTTCCGAAACTGACGGCATTTATTGGGTAAGGTTAGTAAGTTTTATTTTTTGTATTTAGAGCAGCATTTAATTTATACTTTTATAAAAGAAATTTCTAGCTTTGATCCCTCGTTTATCAAACGATACAAATCTATATTCATCATTTTTCTCTCTGGGCACGTTCTCTCTATTCTGGTCTCTCTATTCGGGTCTCTCTCTCTCTTTTCTTTTTCTGTCACTTTCCGGAAATCTTATAGAAAAAGCTCCAGCGGGTATAAAAGCAGCGTCGCTTGGCGAACAGAGC

ACAGATCGAATTCAAAAATCGAGCAGTGAACAAGTAGGAGCAACTTTGCGCTAAAGCAAA

-387 -378 2

-366 -352 3

-75 -56 4

>hsp26_D_yakuba

AGCTGCCGGGAAAAGCACTTCATTACTAAGCGTGGGAGATAGCGGGCGATATATGTATGATTTCCTAAAAACATATGTGACAACAACTACAAGTATTCCAGTAAAACTTAAAGACAGAAACACGAAATAATGTACTTAACAATAAAGAGGAAAACCAGAATAAAAAAAAAACTGACGTTTTGTTTTGTTTGCCGTTAGCCGGCTGTTTCTTTTGCGCTCTTTCTAGAAAATGGCAACAGCTCTCTAGAAACTTCGGTTCGCTCACGCATACAGGCGCACACTGCTCTCTTTTTACGCACAAAACAAGAATTACGTTTTAAATGATTCCCAAAGTTTGCGGCTTTTATTGGGTAAGGTTAGTAAGGGTTATAAATTGTTTTGAGAACAACATTTAATTAAGATTTTTATAAAAATATTTTCAAAATTGTTCCCTAATATATCAAGTGTATAATATAATATTCAGTATATATAATATTCAGTATTTCTCTCTGTGCACGCGCCCTCTCTCGCTCTCTCTCTCTTCTCTTTTTCTGTCACATTCCGGACTCTTCTAGAAAAGCTCCAGCGCGTATAAAAGCAGCGTCGCTTGGTGAACAGAGC

ACAGATCGAAGTCAAAAATCGAACAGTAAGGAAGTCGGAGCAACTTTGCCGAAAAGCAAA

-381 -372 2

-360 -346 3

-73 -44 6

>hsp26_D_santomea

TTTTTTTTTTTGCCGTTAGCCGGCTGTTTCTTTTGCGCTCTTTCTAGAAAATTGCAACCACTCTCTAGAAACTTCGGTTCGCCACGCATACAGGCGCACACTGCTCTCTTTTTACGCACAAAACAAGAATTACCTTTTAATGATTCCCAAAGCTTGCGGCTTTTATTGGGTAAGGGTAGAAGGGGTTATAAATTGTTTTGAGAACAACATTTAATTAAGATTTTTATAAAAATATTTTCAAAATTGTTCCCTAATATATCAAATGTATCAAAGCAATATTCAGTATTTCTCACTGTGCACGCGCTCTCTCTCTCTCTCTCTATCTCTCTCTCTCTGTCTTCTCTTTTTCTGTCACATTCCGGACTCTTCTAGAAAAGCTCCAGCGGGTATAAAATCAGCACCGCTTGGTGAACAGAGC

-378 -369 2

-357 -343 3

-73 -44 6

>hsp26_D_erecta

ACAAATAAAAAGAACCGAGAAAGCTGACGGGAAAAGCACTCAATTACTAAGCGTGGGAGATAGCGGGCGATATATGGATGTATGTATGATTTCCTAAAAACATATGTGACAACAACTACAAGTATTCCAGTAAAACTTAAAGACAGAAACACGAAATAATGTACTTAACAATAAAGAGGAAAACCAGAATAAAGAAACTGACGTGTTGTCTCATTTGCCGTTAGCCGGCTGTTTCTCTTGTGCTCTTTCTAGAAAATGCAACAACTCTCTAGAAACTTCGGCTCGCTCACGCACACGGGCGCACACTGCAAAATTTTCGCTCAAAACAAGAATTACATTTTGAATAATGCCCAAAATTCGCGGCTTTTATTGGCTAAGGTTAGTACATGTTATTAATTTTTTTAAGAACAACATTTAATTTATATTTTATAAAAAATATTTTCAAAATTGTGCCCTAATGAATCAATTGCTACAAAGCAATATACAGTATTTCTCTCTGTGCACGCTCTCTCGCTCTCTTTTCTCTTTTTCTGTCACTTTCTGGACTTTTCTAGAAGAGCTCCAGCAGGTATAAAAGCAGCGCCGCCTGGCGAACACAGC

ACAGATCGAATTCGAAACTCGAACAGTAAACAAGTCGGAGCCCAAAAGCAAAACTTCAAA

-355 -341 3

-335 -321 3

-73 -44 6

>hsp26_D_ananassae

GAACGGGGCCGCTGCTGCCAAGGTCGTCGAGGAGTTGGCCAAGTGAAGACTGAACTAACTGAGGGAAAAAGACAGTAAAATAACAGTACGAGTATGCGTGCGTTTTATTCCATAAATGTTTTTTGTGTTTTCTTTTGTATTTCACACGAAAAACAGTCTAGAAATAGAAGCTTAAAAGGAAACCGGACACTCAATTACTAAGCTCAGCGCAGCGAGATAAAAGTATGATTTCCTAAAACATATGTGACAACAACAACAACTATAGGTATTCCACTAAAAATTAAATAAAAAAAAAACACGAATTATTGTACTTAACAATAAATGGAAAAAAATAAATAAGAAAATAAAGCGAGTACGCAATATTGCACTTTTCTATTTTTGTTTCTCCGTTAGCCGGTTCTGACCTCTCTCTCTCACTCGCTTGGGGGCTCTCATCGTGGGGTTCACTCTCTTCTTGTCACGCACACCTTTGCATTTACTCTCACTCACACAGGTATGACACTCTCTCTCTCTCTCTCTCTCTCGACCTCTCTCGTATTTGCCAGCTTTTTCTAGAAGCCCTCAGCTCGGGTATAAAAGGCGGCGCATTTTTGGCGAACG

GCAGATCGAAAAAAAAAATCATTCTGCAAACATCAGAGACCGAACCGGAAAAGTTCAAGC

-77 -43 7

>hsp26_D_pseudoobscura

GCCGGTGGGGAGAATGGAGCAGCTGCGGCAAAAACCGAAGAGGTGGCCGCAAAGTGAAGACTGAAAGAAAACGAAAAGAGAAAGAGAAAAATAATATGTATGTATGCGTGAGTTTTATTCGTAAATGTTTCCTGTGTTCTCTTTTGGCATTTCGCTATAAACAGACAGAGATAGAAGCTAAGTATGATGAAAAAGAGGGAGATGATGTCGACTCAATTACTACGCGAGCGAGATAGCAGTATGATTTCCTAAAAAATATGTGACAACAACTACAAGTATTCCAGTAAAACTTAGAGGAAAAACCACTTAATGTACTTACAATAAAAACAAATTAAGAGAATTCAAAGTGAGCGGCGTTGCTTTTTTTTTGCCGGTAACTCTTTCTCCCTCACACACAACACACACACACACACGAACAAGGGGGAACAATTTTTGCTCTCACATACACAAACATATGGTGAACGACCGAAAGCTTTTTTGCTCTCTCGCTTTAGCTCTCTCTTTCTGACACACACACACACACACACACACTCCCAGAAACTGCCAGACGCTTCTAGAAGAGCGAAGGGGCTGTGGCGGCTATAAAAGCCGCAGCGCAGA

TCAAAGCGGACTAAAACAACCGTTGACTTCCACCGTGCAAACAGCAAAGCAGAGCGAGTG

-70 -41 6

>hsp26_D_persimilis

AGCCTCTGCCGGTGGGGAGAATGGAGCAGCTGCGGCAAAAACCGAAGAGGTGGCCGCAAAGTGAAGACTGAAAGAAAACGAAAAGAGAAAGAGAAAAATAATATGTATGTATGCGTGAGTTTTATTCGTAAATGTTTCCTGTGTTCTCTTTTGGCATTTCGCTATAAACAGACAGAGATAGAAGCTAAGTATGATGAAAAAGAGGGAGATGATGTCGACTCAATTACTACGCGAGCGAGATAGCAGTATGATTTCCTAAAAAATATGTGACAACAACTACAAGTATTCCAGTAAAACTTAGAGGAAAAACCACTTAATGTACTTACAATAAAAACAAATTAAGAGAATTCAAAGTGAGCGGCGTTGCTTTTTTTTGCCGGTAACTCTTTCTCCCTCACACACAACACACACACACACACGAACAAGGGGGAACAATTTTTGCTCTCACATGCACAAACATATGGTGAACGACCGAAAGCTTTTTTGCTCTCTCGCTTTAGGTCTCTCTTTCTGACACACACACACACACACTCCCAGAAACTGCCAGACGCTTCTAGAAGAGCGAAGGGGCTGTGGCGGCTATAAAAGCCGCAGCTCAGA

TCAAAGCGGACTAAAACAACCGTTGACTTCCACCGTGCAAACAGCAAAGCAAAGCGAGTG

-70 -41 6

>hsp26_D_willistoni

CAGTCTAACCAACATTCTCTTGCACTCATCACTCTCATCGCACACACACACACACACACATTAAAAAAGCTTCGCGCTCTCTTTATTCTAGCAGAGGGTGGACTCTTCTAGAAGAGAAGCGCTCCTCTATAAAATCAGCAGCAGCATTTGTTCTCTTTCTTCGACGTTCGATTCCGAGGTTGCATGTTCGACTCTATTGTCGGAGTTTGACTCCATTGCTCGATTCCGACCATTTCTCACGGTTTTTACTCTCTTTTTACTCTTTGTTGCATGTTGGATCGAGCTGCGACATCGAGTTGCGATCCCCCCTTGCAGTGGTGCCGTCAAACATAGTCATTTTTTTTGTTTCACGTCGTCTCTATTAGAAACAACCGTTAATCGTGGTACATTGCATATACATATGTACATATTTTGCAATTGAGTTAAGAAACAGCGAAGAGTGAGTAACAGTCTAACCAACATTCTCTTGCACTCATCACTCTCATCGCACACACACACACACACACATTAAAAAAGCTTCGCGCTCTCTTTATTCTAGCAGAGGGTGGACTCTTCTAGAAGAGAAGCGCTCCACTATAAAATCAGCAGCAGCATTTTGTT

TCACGTCATATAGAAAAACAACCGTTAATCGTGGTACATCTAAGAAAGTTGACAAAGAAG

-516 -487 6

-69 -40 6

>hsp26_D_mojavensis_A

ACAGCCCTACAAATATATTTTGAATGCAATTTCTAGGTTTCCCAATAAATGCAATTTGAAAATATAAAAATAAAATCGTAGGCATATGTATGTATGTACATACATATTATGTTTGTATGTATGTACGTACACTCATACATCCGTATATGTATCTATGTATGTATGTATGTATGTAGTCGTATATGTACATAATCAGACGTGTGCATATACATATGCACATATGGATGTACATATGTATGTATGCATGTCTGTATGTAAATATAAACATATATTACATAAATAAAAGGCTTTTTAAAAAAAAAAAAAAAAAAAAAAAAAAAAAAAAAAAAGGAGTTTGGAATGAGCTTCCGAAGTTGCCTGTTTTAATTGCTTTTGTTATTTAGTTTGACTTTTTGCACCGTCTGACATTTTCTCCCGCTCTCACACACACAATATTAACACGCATTCTCTCGTATATTCGATTCTCTTTTTTTGTGACACACACGAACACTCTAGCAAGCACGCGATGTTTCTCGCTCTGTTTCTCCGATTCGCACTCAACTCGTAAAAGCTCGATGCTTCTAGAAAACCACTCAGCCGGCTATAAAAGGCCGCTGGCTG

AGTTCATTGCTTCAGTTGAAAAATATCAATCATCAAGTGAAGATATTCACAGCGCTTAAA

-58 -34 5

>hsp26_D_mojavensis_B

TAAGGACGCAGAGGAGCCAACCCTGGCAGCGGCTAAGTGAATGTAGAGACTGATAATTGATGCGCAACGCAGAACTATAAAAGAACTAAAAAAAGAAAAACAAAATATAATAACGATAAATAATGATAAACAAATATCAAATTGGAATGCGTTTTAATCTATAAATGTTTTATGTGTTTTTTCAATAATTCGCAATAAATACATGAGATAGGGCATGCAGGTGGACGAAATGCAAAAGGAGAAAGAGATGAGAAACTATGATATGAAATTATGATTTCCTAAACAAGTGTGAAACAATTACGACTACAAGTATTCAGTAAAACTAAGCACATTATGTACTTAAAACTTACAAGTAAATAAAGATTCAAAGAGTGCGTTTTGTGTTTTGTCTTTTCACTTGCTTTTCTTTACTCATTGCTGTTGTTTTCCAGTCTATGATTCTCTTCTCTTCACACAGGCATACTCTTTCTAGTTACGCTCGCTCAGTGGAGAGCATACTCTTTACAGCTATGCGATGTTTTTTCATCTTTCTCACTATGCTCTAGCAATTGAGCGATGTTTTCAGAAGAGCACGATTGCCGGCTATAAAAGCAGCAGCCG

ACAGGCTGCGATGTTACAGTTGAACAACAGTATTAGTCAAAGTGAAGTTTCGCTGAGCGA

-62 -33 6

>hsp26_D_virilis_A

ACATACATACGTATGTATGTGCAAATAAGCGACATACACATATTATCTGTGTATGTATGTATGTACTTTGTTTCATCATGCGTAAAAGCATACATATGTACATGTGTGTGTGTGTGTATGTATGTGCAAATGCATTCTTCATGGATCCTAATTTTAATGAACTGCAAATTTAAAACGCTTTAAGCTGTCTGTTAATAAGAGTATATTAACATTAACATGTACATGTATGTATATACATAACGACGCATACATGTGTGTATTTATGTATGTACGTACGACATCTGTGTATGTATGTTTCGAAAAATGTATTTTTATAAACAAGGAGTTCAAAAAGTGCAATTAAAGTTGACTTTAAAATTTGCTTTGACTTTTTACAGTCTGCCAAGCGAACTCTTTCTCTTTCGCATACTATATTTACCCATACTCACACTCTTAGCACTCTTTCTGTTTCACTCATTTGCGTCACGCACACACTCTCGCAAGCTCGCGATGTTTCTCGATTTACTTCTCTGTTCGACACATAGCACACACACACGCACGAGCAATAGCTCGATATTTCTAGAAAATCGATTCAGCCGGCTATAAAAGCGGTTGTCGAAC

TCAGTTTGCCAACAGTTGAAAAAAAGTGTTCGTCAAGCAAAGAACTTCGCCGTGCGAAAA

-128 -104 5

-60 -36 5

>hsp26_D_virilis_B

GCAGGCCAGCGAGAACGGCGCGCAAGGCTGAGGAGCCGCCCCTAGCAGCGGCCAAGTGAATGTAGAGACTGACAGAATTGATACACATGGCAGAGAACTAAACAAAAACTAATAATAATAATAATAATAATGATAATGATAACAGAACAAAATATCTAATTGGAATGCGTTTTATTCTATAAATGTTTTTTGTTTGTGTGTGTTTTCAATAATTCACAATAAATAATACATGATAGGGCCTGCAGGTAAACGAGATGAGAACTATGATATATGAAATTATGATTTCCTAAACAAGTGTGAAACAATTACGACTACAAGTATTCAGTAAAAATAAGCGTATTATGTACTTAAAATTTGAAGTAAATAAAGATTCAAAGAGTGCGTTTGCGTTTGCGTTTGCCTTTGCTTTTCTTCGCTGTTATTTCCCCGTCTATGATTCTCTTTTACACACAGCAACAGCAACAGCAACTCTTTCCAGCTGTTTTGCTCTCTGAGTTACACATACTATGACAACTGTGCGATGTTTTTTCTTCTGCTCTCTAGACTCTGCGCGATGTTTCCAGAAGTGCACATTTGCCGGCTATAAAAGCAGGCGCCAAC

TGCTGTGCCGTTACAGTTTAACAACAGTATTCGTCGAGAGTAGAACTTCGCTGTGCGAAA

-64 -35 6

>hsp26_D_grimshawi

ACTTAAAAGAGAGACTGACTGATAAAGTTGATGCGCAAGCCACAAAATTAAAACTAAAAACTAAAAAACAAACAAACAAATAAGAAAAAGTAGAAAATCCACTTGGAATGCGTTTTATTCTATAAATGTTTTTTGTGTTTATCAATAATTCGCAATAAATACACACACAACATACAAACATATGTAATAGCAGCGAGAAGAGAAAATAGAGGAGAATTAGAGGCGAAGAGAGGCCAACTACGCTATAAAATTATGATTTCCTAAACACAAGTGTGAAACAATTACGACTACAAGTATTCAAGTAAACATAAGCACATTATGTACTTAACATTTAACAGTAAATAAAGATTCAAAAAGTGTGTCTTTGTGTGTGTTCTTTCCCTCTTGCCGGCAATTAAATTTATTCTATTTCTCTTTAGACGACCGGCATTTATAATTCTCTTCACACTACTCTCTAAATATTTCGACCCGTTTGTGGTGAATAACGTGCTCTCTCTCGCACATATACTTGAACTGTGCTGTGCGATGTTTTTTCGCTCTCACCACAAAAGTTGTGCGATGTTTCTGGAAGAGCAGCGTCTATAAAAAGAGGCGGCAAAT

GCAGTTCCGTTACAGTCGCAAGACAGTTATCATCAAGTGCATACAAACTTAACTTCGCTG

-44 -30 3

>hsp27_D_melanogaster

AACAAACAAAAGAACGGCAAACATGAGGAGCAGAACGAAGCGAGACAAGGGTTCAATGCACTTGTCCAATGAAAATACAAGCTCTGTTGCACTCTGAAAAGACAGCTTTTAAAAGAGCGATAAGAGAAGAAAATGCTTTAAATAAATACATATATCTGCATATATACGTACATGTACATACATATGTATGTACTGCATTTTAACTGTTCGTTTTGCTTTTTATTCGCAAAGAGAAACTCCCCAGAAAAGAAATGTCAAGAAGTTTCTGGTTCTTTCTCCCTCTCTCTATGAAAAGCCGGCTGTGCTAGAAAGAGCCAGAAGATGCGAGAGAAAACTGTTTGTTGAATTACGGGGCGTATTCAAAGGGGCTTTTAAATGTCGCTTAAATTTTAAGTTTGACAGGCTAATAATTGCTTGCCTATATCTAAATATTATTATATTTGCATTAGGGGATCATAGGGAAAACCTTCTCTGCAGGCAAAATCTAACGAAGATGGCAACCCCCCATCATTTTATTAAAGTTCCGTCCCTGGTTGCCATGCACTAGTGTGTGTGAGCCCAGCGTCAGTATAAAAGCCGGCGTCAACGTCGCCCGAGCAC

AGTCTAAACTGAAAAATTGAAGGCAAACGTTGAAGCAAACTTCGCTAAAAAAATTCGAAA

-369 -360 2

-353 -324 6

-299 -270 6

>hsp27_D_simulans

AACAAAAGAACGGCAAACATGAGGAGCAGCACGAAGCGAGACGAGGGTTCAATGCACTTGTCCAATGAAAATACAAGCTCCGTTGCACTCTGAAAAGACAGCGTTTAAAAAGCGCGATAAGAGAAGAAAACGCTTTAAATAAATATATCTGCATATATATGTACGTACATGTACACTCATACATATGTACTGCATTTTAACTGTTCTTTATGCTTTTTATTCGCAAAGAGAAACTCCCAGAAAAGAAATGTCAAGAAGTTTCTGGTTCTTTCTCGCTCTCTCTATGAAAAGCCGGCTGTGTTAGAAAGAGCCAGAAGATGCGAGAGAAAACTGTTTGTTGAATTACGGGGGCGTATTCAAAGGGGCTTTTAAAGGTCGGTTAAATTTTAAGTTTGGTAGGCTAACAATTGCTTCCCTATATCTAAAAATTATAATATTTGCATTAAGGGATCATAGGCGGAAAACCTTCTCTGGAGGCAAAACCTAACGAAGATGGCAACCCCCCATCATTTTATTAAAGTTCCGCCCCTGGTTGCCATGCACTAGTGTGTGTGAGCCCAGCGTCAGTATAAAAGGCCAGCGTCAACGTCGCCCGAGCAC

AGTCTAAACTGAAAAATTGAACGCAAACGTTGAAGGAAACTTCGCTCAAAAAAAAAAAAA

-372 -358 3

-357 -328 6

-298 -274 5

>hsp27_D_sechellia

TTGCAGCAAACAAAAGAACGGCAAACATGAGGAGCAGCACGAAGCGAGACGAGGGTTCAATGCACTTGTCCAATGAAAATACAAGCTCCGATGCACTCTGAAAAGACAGCGTTTAAAAAACGCGATACGGAGAAGAAAATGCTTTAAATATTCTGCATATATATGTACGTACATGTACACTCATACATATGTACTGCATTTTAACTGTTCTTTATGCTTTTTATTCGCAAAGAGAAACTCCCAGAAAAGAAATGTCAAGAAGTTTCTGGTTCTTTCTCGCTCTCTCTATGAAAAGCCGGCTGTGTTAGAAAGAGCCAGAAGATGCGAGAGAAAACTGTTTATTGAATTACGTGGCGTATTCAAGGGGCTTTTAAAGGTCGGTTAAGTTTTAAGTTTGGTAGGCTAACAATTGCTTCCCTATATCTAAAAATTATTATATTTGCATTAAGGGATCATAGGAAAAACCTTCTCTGGAGGAAAAACCTAACGAAGATGGCAACCCCCCATCATTTTATTAAAGCTCCGTCCCTGGTTGCCATGCACTAGTGTGTGTGAGCCCAGCGTCAGTATAAAAGGCCGGCGTCAACGTCGCCCGAGCAC

AGTCTAAACTGAAAAATTGAAGGCAAACGTTGAAGGAAACTTCGCTCAAAAAAAAAAAAA

-368 -354 3

-353 -324 6

-294 -270 5

>hsp27_D_yakuba

ATGGGGAGCAGCACGAAGCGAAACGAGGGTTCAATGCACTTGGCCAATGAAAATATAAGCTCCGTTGCACTCTGAAAAGACAGCGTTTAAAAAGCCCGATAAGAAAAGAAAATGCTTTAAATAAATACATATATCTGCATATGTACATACATACGTACTTACATAGGTTCTGCATTTTAACTGTTCTTTATGCTTTTTATTCGCAAAGAGAAACTCCCATAAAAGAAATGTCAAGAACTTTCTGGCGCCCTCTCGCTCACTCTATGAAAAGCCGGCTGTGCTAGAAAGAGCCAGAAGGTGCGAGAGAAAACAGTTTGTTGAATTCAGGGGCGTATTAAAGGGGCTAATAGGGGTCACTTAAATCTATCGCTGTGCAGGCTAACGATAACTTACACTTGCTTTGAAATCACAGAATAACCTTTAGATAAATTCTATTATATTTGCGTTACGGGCTCAAAGGAAAAACCTTCTCTAATGACAAAACCTAACAGAGATAAAGGCACCCCATCATTTATTTAAGGTCCGCCCCTGGCTGACTTACACTAGTGTGTGTGAGCCCAGCGTCGGTATAAAAAGCCGGCGTCGACGTCGCCCGAGCAC

AGTCGAAACTCAAAAATTGAAGGCAAAAGTTGAAGGAAGCTTCACTCGAGACTAAAACCA

-392 -378 3

-377 -348 6

-323 -294 6

>hsp27_D_santomea

GCAGCACGCAGCGAAACGAGGGTTCAATGCACCTGGCCAATGAAAATATAAGCTCCGTTGCACTCTGAAAAGACATCGTTTAAAAAGCCCGATAAGAGAAGAAAATGCTTTAAATAAATACATATATCTACGTACATACGTACTTACATATGTATGTAGGTACTGCATTTTAACTGTTCTTTATGCTAAATCTTTATGCTTTATTCGCAAAGAGAAACTCCCAGAAAAGAAATGTCAAGAACTTTCTGGCGCCCTCTCGCTCGCTCTATGAAAAGCCGGCTGTGCTAGAAAGAGCCAGAAGGTGCGAGAACAGTTTGTTGAATTCAGGGGCGTGTTAAAGGGGCTAATAGGGGTCACTTAAATCTATCGCTGTGCAGGCTAACGATAACTTACAATTGCTTTGAAATCACAAAATAACCTTTAGATAAATATTATTATATTTGCATTACGGGGTCAAAGGAAAACCCTTCTCTAATGCCTAAACCTAACAGAGATAAAGGCACCCCATCATTTATTTAAGGTCCGCCCCTGGCTGACTTACACTAGTGTGTGTGAGCCCAGCGTCGGTATAAAAAGCCGGCGTCGACGTCGCCCGAGCCC

-388 -374 3

-373 -344 6

-319 -290 6

>hsp27_D_erecta

GGTGGGAGAGCAGTACGAAGCGAAACGAGGGTTCAATGCACTTGTCCAATGAAAATGTAAGCTCCGTTGCACTCTGAAAAGACAGCGTTTAAAAAAGCCCGATAAGAGAAGAAAATGCTTTAAATAAATATATCTGCATACAAATGTATGTACATACGTACTTATGTACTGCATTTTAACTGTTCTTTATGCTTTTTATTCGCAAAGAGAAACAGCCAGAAAAGAAAAGTCAAGAACTTTCTAGCGCCCTCTCGCTCACTCTATGAAAAGCCGGCTGTGCTAGAAAGAGCCAGAACATGCGAGAGAAAACAGTTTGTTGATATCAGGGACGTATTGAAGGGGCTAAAAGGGATTGCTTAAATTTTTAAATCTGAAGGATAACGATAACTTACAATGGCTTCGAAAGCGCAGAATAACTAATATCTAAATATTATTATATTTGCATTACTGGTTCAAAGCGACAACCTTCTCTGGAGCCAGAACCTACAAAATATAACAGCCCCCCATCATTTTATTTAAGATCCGCCCCTGGCTGCCTTGCACTAGTGTGTGTGAGCCCAGCGTCGGTATAAAAAGCCGGCGTCGACGTCGCTCGAGCAC

AGTCGAAACTCAAAAATTGAAGGCAAACGTTGAAGGAAGCTTAGCTCAAAAAAAAAAAAA

-393 -379 3

-378 -349 6

-324 -295 6

>hsp27_D_ananassae

GTATGTACATGCATTTTTATGTAGCATGATGTATGTATGTATTGTATATTGTATGTTGCATGTTGTATGTTATGTTGTATGTTGCAAATCGTTGAATTGCCCTTGTGCCTGACTTCGGGAAGTAAAAGAGGAGCGCCACCAGAAGCGGCTAAATGTTTGCAGCAAACACAAAGAACTCAAACACGAGAGAGCCGAGCGAGACGGAAAGTTCATCGCCCCCAACTGCAATAAAAGTTCGCGGATGCAGCCGACTCCAATGCACTTCACCCAGACGGTTCTTGACGTTTAAACAATTCACGGCGATAAGAAAAGAAAATGCTTAAAATAAATATGCACATACATATGTACTGAACAGTGAGTAATTTAACTGTTCTTTTTTATGTTTGCCAAGAGAAGGTCCCAGAAAATGCAAAAATTTCTTTTTTCCCCAACACACACACACACACGTAGCTTTTCTCTCCCTCTCACGCAATCCAAAGCCGGCTGCGCCAGAAAGAGCTAGAACTTGCGAGAGAGCTAAAGAGAGAACGAGAACACTTTTCTGTGTGTGTGTGAGCCGGCGTCGGTATAAAAGGCCGGTCCCGACGTCGGCCGCGCCGC

ATTTTGAATCTGACAAGCTTTTCGGAGTGCAAGCTTAAAAAAATCTGTTTTTTTTTGTCC

-209 -185 5

-115 -66 10

>hsp27_D_pseudoobscura

ACAGCAACCGACTGCACATATTTTTTTAAATTCTGCTTTACTTGAGATTTCTCAGCAGCAGCAGCCGCAGCAGCAGTGGCTGCAAAAATGTTTGGCACAAAAAAAACGCAAGCACAACTACACTCTCGTACATACACACATACATATGTATGTATGTATGTGTACATATAAAACAGAAACGGCGCAGGTTCAATGTACTTTCCAGCTAATATCAATGCACTGGGCTGCGCCACAGACAGCAGCACGAACGTGACTTCAAACATTTAAACAATTGAATTTACAACAACTTCGATAAGAAAAGAAAATGATTTAAATAAACGTGTTTGTTGTATATACATATGTATGTTCTTACATATGTATGTATGTACATACATATACATGGCTATTTAATTTGCGTTGAGAATCTCCCAGAAATTGCAAGAACTTTCTCACACACACACACAGTCAACCACCGGCATTGCGGTGGTGCCGGCTGTGCCAGAAAGAGCGCGAAGCTTCCACAGAGCGCGAGAGAGAACGAGAAAACAGCTTGTTGCTTGCGCCAGTGTGCGTGAGCAGAGCGTCGGTATAAAAACCCGACGTCAAGGGCGACCAGTTAAC

AGTTGCATTTCAAATCATGTAAAAGCATCAACGCGAGAGCATTTTTTTTGTCCATACAAA

-201 -172 6

-126 -77 10

>hsp27_D_persimilis_A

TTTTTGCACATTGCCACATGCAGCTGCCACAGCAACCGACTGCACATATTTTTTTAAATTCTGCTTTACTTGAGATTTCTCAGCAGCAGCAGCCGCAACAGCAGTGGCTGCAAAAATGCTTGGCACAAAAAAAACGCAGGCACAACTACACTCTCGTACATACATATGTATGTATGTGTACATATAAAACAGAAACGGCGCAGGTTCAATGTACTTTCCAGCTAATATCAATGCACTGGGCTGCGCCACAGACAGCAGCACGAACGTGACTTCAAACATTTAAACAATTGAATTTACAACAACTTCGATAAGAAAAGAAAATGATTTAAATAAACGTGTTTGTTGTATATACATATGTATGTTCTTACATATGTATGTATGTACATGGCTATTTAATTTGCGTTGAGAATCTCCCAGAAATTGCAAGAACTTTCTCACACACAGTCAACCACCGGCATTGCGGTGGTGCCGGCTGTGCCAGAAAGAGCGCGAAGCTTCCACAGAGCGCGAGAGAGAACGAGAAAACAGCTTGTTGCTTGCGCCAGTGTGCGTGAGCAGAGCGTCGGTATAAAAACCCGACGTCAAGGGCGACCAGTTAAC

AGTTGCATTTCAAATCATGTAAAAGCATCAACGCGAGAGAATTTTTTTTGTCCATACAAA

-195 -166 6

-126 -77 10

>hsp27_D_persimilis_B

CACATTGCCACATGCAGCTGCCACAGCAACCGACTGCACATATTTTTTTAAATTCTGCTTTACTTGAGATTTCTCAGCAGCAGCAGCCGCAACAGCAGTGGCTGCAAAAATGCTTGGCACAAAAAAAACGCAGGCACAACTACACTCTCGTACATACATATGTATGTATGTGTACATATAAAACAGAAACGGCGCAGGTTCAATGTACTTTCCAGCTAATATCAATGCACTGGGCTGCGCCACAGACAGCAGCACGAACGTGACTTCAAACATTTAAACAATTGAATTTACAACAACTTCGATAAGAAAAGAAAATGATTTAAATAAACGTGTTTGTTGTATATACATATGTATGTTCTTACATATGTATGTATGTACATGGCTATTTAATTTGCGTTGAGAATCTCCCAGAAATTGCAAGAACTTTCTCACACACACACACAGTCAACCACCGGCATTGCGGTGGTGCCGGCTGTGCCAGAAAGAGCGCGAAGCTTCCACAGAGCGCGAGAGAGAACGAGAAAACAGCTTGTTGCTTGCGCCAGTGTGCGTGAGCAGAGCGTCGGTATAAAAACCCGACGTCAAGGGCGACCAGTTAAC

AGTTGCATTTCAAATCATGTAAAAGCATCAACGCGAGAGAATTTTTTTTGTCCATACAAA

-201 -172 6

-126 -77 10

>hsp27_D_willistoni

AAATGTTTGCAAAAAGAAGCGCAGACGAACAATGCGAAGCGACAGAGAGAGAAAGTTCAATGCACTTTTTTCAGTGTCTCTTTGCTCTTGAATATTTCAAACAGCAGCATCGGCTGCAAATTTGCGATAAGCCAAGTGCTTTAAATAAATGTTTATGTGCACACATATGTATGTACATACATACATACATATGTATGGCATGTATGTACATATGTACATAAATAATGAATTGTACACGAAGTACATTGTACATACATACGTATGCAAAAGTCAAATGCGTTCACAAATATGTACATACATATGTACGTATGTATATAAAACATAAACATTTATAAAAACTTCTTTAGCACAAAGTAAGAGAACTTTGCAGAAATTGCAAGAATATTTCGTGCAGATGCATACAGTTACATGCTTGTATACATACATACATGCATACGTATGTATTTTTACTCTCTATACTTTCGAGTTAGTTCGGGAAGTTCGCGAGAAGCTTTGAGAAATTTCGAGAGAGAGCGAGAACGAACACAAAGAGAGCGTAAACAGCTTGTTGCTTGCCGGCGTGGGTATATAAAAGGAATGAAGAGCAGCTGCTCGCTTTAC

AGTTACTTTTACAAACTTCTGCCGTGCAGAGATACGCTTAGTGAGAACAAAAAGAAAAAG

-242 -218 5

-141 -122 4

-120 -81 8

>hsp27_D_mojavensis

ATTACATATGTATGTATGTGTGTATATACATGATTGCAATGCAGAGAAAAGAAAACGAGGATAAAGAATGACAAACAAACCGGTCTCGGCAAACAGAGACTGCAAGTTCATTGTACTTGCAAAATGGCAAAGTTGACGTCTGAGTGGATGCGTTTTTCTGCCAGTGGTCCCCTAAATATACTTTAACATTTTAGCAAAATGCCATACGAATTTTAAAATGCAGGCGGTTTAAATAAAGAAATACTTAAAATATCGTATATGCACATAGTACATGCATACATACATATAGTATTTGTATAGGCACATGCATGTACGTAGTATGTATTTACACACATACATACATATGTATGTATTATTTACAAATTTACCACGCACAGAAATTTCAAGAAACTGGTAGATTTTTATTTGTGGCAAAGAGAGCAAACAAAATAAAATGCCGGCTGGCACACACATACACACACATACGTACATATTTACTTGCCGACAGAAAGAGCGAGTAAATTCAAGAGCGAACGAGAACAATCACCAATATGTTTTCTCTGTGTGTGTGAGTCAGTTTGGCGTCGGTATAAAAACCCGCTGCGTTCCTTGCGCCTCTTC

AATTCTATTCAAACCGTCGTTTAAAACACGTCTGCTACGCAGAAGCAATAAAAAAAGACA

-225 -201 5

-115 -76 8

>hsp27_D_virilis

GAACACCGAAATCGGTATATAAATACATACTACATACACACGCACACATATAGACCGGTATATACATGCATGCATATGCATGTTTGCACATACAATACATAAATAAAAAGAATGACAAACAAACCGGTCTCGCCAAACAGAGATAGCACGAGTTCATTGTACTTGCGAAACGTCAGTTTCAATGTATGCGATTTTCTCCCTGTGGACCCTTGAACGTACTTTAACATTTTAAACCGATGAATTTACAACAATGGCGCAAATGCAATAGGGATATACATGTATGTATGTTAAAATACAGGCTCTTAAAATAAATGTACTCGAACATACAATGTATATGCAAAATATACGTACGTACATATGTATGTATTTGCGAACAAGCAGAAACTCCAAGAAATTGCAAGAACTTTCCGTGTGTGAGAGAGCCAGAAAGATCGAGAGAACAAACAAAACAATTTGCCGGCGCACACAAACACACGCATGGCGGCAGAAAGAGCGAGTAAGTTCAAGAGTGAAAAAGCAACAATCAGTTAACTTCTTGCTTGTGTGTGTGTGTCATGTTGGCGTCAGTATAAAAACCCGCTGCGCCGTCCGCTCCTCTTC

ATTTCTATTCGAACCGTTGTTCTAAACGTATCTGCTGCGCAGAAGCAAACAAAAAAGTTT

-221 -192 6

-186 -162 5

-115 -81 7

>hsp27_D_grimshawi

AGTTTATTCAAGTAATTTAACAGCAAATTGAGCATTAAAAATGTTTGCTACTAATACATACATACATTCGAAAGAACCACATACACATGCAATTTTCGGCAAAGGGAGGGATTCATGTACATATGCACACATACACCTTTACATTTTTTTTACATTGAAAAATAAGAGAGTGACAAACAAACCGGTTGCGGCAAACAGAGATAGCGTAAGTTCAATGCACTTCCGAAACGTCAGCCACTGTCCCCCTTTGAATCATTACATGTACTTTAATATTTTAAAACATTGCATTTACAACAGCAGCAATAATGAGATGAAAATTGAGCATTTCAAAATGCATGAGCTTTACTTCTATGCTTCTGTGTGTAATAGAGAGCGAAAGGGAGAGAACGAACATGCAAAACATATTTTGCCGACAGACTCACACATACACATGCATGTACAGGCCACAGAAAGAGCGAGAAACTTCAAGCATGGCCACAGAAAGAGCGAGAATTTTCAAGAGCACAACAGCAACAATTGTTGATTTAACTGCCAATGCTTCTGTGTGTGTGTCAACTTGGCGTTGGTATAAAAAGCCCGCTACGTCAACCTCTCCTCTAC

ATTTGTATTCGAACCGTTGCACGAAATACGACTGCTGCGCAGAAGTAAGCAAAAAGTCGA

-153 -129 5

-122 -88 7

>hsp40_D_melanogaster

CTTTGGTTGAACAATATAAGTGCAACTTTCTCCATCACCTTCCTATCTTTTTACAATATGCTTACCTCGTCAATACGTTTTTTCTATTTCAAATATTTCAATATTTCAAAGAAATATTTTGTTTATTTTTCTGTGTGTTTTTAAGCAATCTGACCCCTGTAGAAGAATCCCTTATAATATTAACAAATGTATCCTCAAAATAGATCGATCTCTATCTTCGCAGACTTACACGAAACATTCCAGAACCGATAGTTTTATGCGATATATGAGATTTAAGGAGTACTTTCCGCATTTCGCCATCACAGTCACGCTTTCCTTGGCATTTGCAATCAAATAAGCGCTAATAATAATCGTAAAAGCATAAGAAGCATATAAAGAAGAGTCACCGCCAAAAGCATGCACAAATATATATAAATGGGGAGCGATTTAAAAACAGTGCACTGTGTTTAAAACATCGACAGCTATCGGTTAGCATATCGATATTGACATTCGCAGTCAAACGTTTTCGAGATACAACCCTAAAATCCGAGAAGCATCCAGAAATTTCGACGTAGACGAGGGCGAACCTATAAAATGAGGTTGACGCACGAATCCCGCTCA

TTATTAAACAAAATTTTGAAGAGAAAGAAACTCTGAAGTAGGGTGTGTTTTTAGTGCGCA

-385 -366 4

-364 -355 2

-97 -53 9

>hsp40_D_simulans

TTTTTTAAACATCACATCACATTCCACACCTCTCGACTGTATTATATTTTATATCATCTAGAGATTATTTATTATTATTTATTTATTTCTGCTGATCTTTCGATAGTAAATTATTTAGTTGAAAAATATAAGTGCAAATTTCTCCATCACTTTTCAATCTTTTTATAATATGCTTACCTCGTCAACGGTTTTGTTCTAGTTAAAATAAATATTTTGTTTATGTTTCTGAGTGTTTTTAAGCAATTGTAGAGAATCCCTTTAGGATATCAAAATATATATCCCCAAAAATAGATCGATCCATAACTAGGAATTCGAGGACTTTCACGAATCATTCCAGAACCGATAGTTTACTTACTTTTCCCACGTCGTTTACAGTCACGCCTTCGTAGGCATTCGCAATCAAAGAAGCATATAAATAAGAGCGATTTAATAAAAGTGCACAGTGCTTAAAACATCGACAGCTATCGATAAGCATATCGATATTGACATTCGCAGTCAAACGTTTTCGAGATACAACCCTAAAAACCGAGAAGCATCCAGAAATTTCGACGGAGACGAGGGCGAACCTATAAAATGAGGTTGACGCACGAATCCCGCTCA

TTATCAAACAAAATTTTGAAGAGAAAGCAACTCGAAAGTAGAGTGTGTTTTTAGTGCGCA

-291 -272 4

-270 -261 2

-97 -53 9

>hsp40_D_sechellia

ATGTATTATATTATATATCATATAAAGATTATTTATTATTATTTATTAATTTCTCCTGATCTTTCGATTATAAATTATTTAGTTGAACAATATAAGTGCAAATTTCTCCATCACTTTTCTATCTTTTTATAATATGCTTACCTCGTCAACGGTTTTTTTCTAGTTAAAATAAATATTTTATTTTGTTTATGTTTCTGAGTGTTTTTAAGCAATTGTAGAGAATCCCTTTAGGATATCAAAATATATATCCCCAAAAATAGATCGATCTATAACTAGGAATTCGAGGACTTTCACGAATCATTCCAGAACCGATAGTTTACTAACTTTTTCCCACGTCGTTTACAGTCACGCATTCGCAATCAAAGAAGCATATAAATAAGAGTCACCGCCATCAACATGCGCAAATTTATGTAAACGATGAGCGATTTAATAACAGTGCACAGTGCTTGAAACATCGCCAGCTATCGATAAGCATATCGATATTGACATTCGCAGTCAAACGTTTTCGAGATACAACCCTAAAAACCGAGAAGCATCCAGAAATTTCGACGGAGACGAGTGCGAACCTATAAAATGAGGTTGACGCACGAATCCCGCTCA

TTATCAAACAAAATTTTGAAGAGAAAGCAACTCGAAAGTAGAGTGTGTTTTTAGTGCGCA

-322 -303 4

-301 -292 2

-97 -53 9

>hsp40_D_yakuba

AATTAGGCGTTTAAAATTCATCTTTTTTTATATACAAAGTTATTTTGTGCAATTTGTTATAAAAATATTTTTTAATTTTAATACTACATTGGGATCGAATGTATGTTAAATTATATTATCTAAAGTTATTATACAAACCCCATAAAATTCCTCTTATTCTAAAGATTGTTGTTTTCTAAAAATTTACGAAAATTGTTAGCTTCTTTTATTAGTTTTCTTTACCTCGACAACAGGTGATTTGTTTAAAATTTATATGAAATACACATTTTTGTTTGTTTTTAAACAATTGCATAGATCTATAGACCGCAGTCTTGTAAAATGCATCGGCGTATTTTTTAACTAAAAATAGTTCGCGAAATATAGGTCGATTTATAACTAAACATTCGCGGTCTTTCAGGAAATATTCCAGAACCGATAGTTTATGCGATATGTGTGACTTTAAAATCTTTTGTCTACCCAAATCGTTCGCAGTCACGCTTTCGTTGCCATTCGCACTCAAACGTTTTCGAGATACAACCCTAAACGACGAGAAGCATCCAGAAACTTCGACGGAGACGAGGGAAAACCTATAAAACGAGGTTGACGCGCGAATGCCGCTCA

TTATCAAACAAAATTTTGAAGAGAAAGGAACGCGAAGAGGAAAACAGGGTGTGTTTTCAG

-219 -200 4

-198 -189 2

-97 -53 9

>hsp40_D_santomea

CATTTAAAATTCATATTATTTTATATACAATTTCATTTTGTGCAAATTGTTATTAAAATATTTTTTAATTTTAATACGACATTTATTCGATTCCATGTTAAATTATATTATCTAAAGTTATTATTATACAAACACGGTAAAATTCCTACTATTCTATTGTTAAGTTATTTTCTAAAAATTTACGAAAATTGTTAGCTTCTTTCATTAGTTTTCTTTACCTCGTCCACAGGTTATTTTTTATTTTGATATTTTTATGAAATAAACATTTTGGTTTGTTTCTAAGCAATTGTATAGATCTAAAGACCTAAGGTTTTTAAAATTCATTGACTTATCTTTTAAGCAAATATAGTTCGTCAAATATAGGTTGATCTATAACTAAACATTCGCGACCTTTCAGGAAATATTCCAGAACCGATAGTCTATGCGATATGTGTGATTTTAAAATCTTTTGTCTGCCCAAATCGTTCGCAGTCACGCTTTAGTTGCCATTCGCACTCAAACGTTTTCGAGATACAACCCTAAACGACGAGAAGCATCCAGAAACTTCGACGGAGACGAGGGAAAACCTATAAAACGAGGTTGACGCGCGAATGCCGCTCA

-219 -200 4

-198 -189 2

-97 -53 9

>hsp40_D_erecta

GTGTGACTGTCCGCCGCTGTCTGCGAAATACCAAACGCCTTTGAACTGCGGTAATCGCTAAGTCTACGTCTTAGGGTCACACTGGCAGCGTTTAAAACATTTCTTTATTTTAATTTTCAGTTACTTTTCTGATTTACTTATAAGTTGGTCTGAGTATTACACAAATAATTTAATTAGACATTGAAAAATATCTTTTTACTTTGTTAAAATTTTTATTATTCAAATTTTGCACTACATTTATTCGACGCCTATCGGCGGTTTTTTAATTTATTTTATCTAAAAATAATTATTAAGTCAATATATTATTCTATCGATTGTTAAGTTTTTTTTTTTAAATTGAAAAATATTAAAAAAATATCGCAACGTACATCCCTTATTTTTTCGCCAAAAATAGGTAGATCGATCTATAACCATCCAGAACCGATAGTTCTATGCGATATGTGTGATTTTGTCTCCCCACATCGTTCGCAGTCACGCTTTCGTTGTCATTCGCATTCAAACGTGTTCGAGATACAACCCTAAAAAACGAGAATCATCCAGAAACTTCGACGGAGACGAGTGAAAACCTATAAAAGGAGGTTGACGTGTGAATGCCGCTCA

TTATCAAACAAATTTTTGAAGAGAAAGGTACGCGAAGAAGAAAGTAGGGTGTGTTTTTAG

-189 -180 2

-179 -170 2

-97 -53 9

>hsp40_D_ananassae

AGATTGATAAAGAATATATCTACAAATCTTTTAAGGTATCCCGATTAAAAAATAGGATGATATTTCTGAAGATATATTGAACACGTTTAGAATGACTAGGATTTGAAAATTCAAATTTAAATTTATTCTGTCGCTTTGCAACACTGTCTGTTGTTCCTCTGTCTGTCGTTTTCCTCTGCTTATATTAACGAAAAGAAAAAGATTGAAAACTGCAAGTATTTTTAAAATATTTGCGTAGAATTGTTTACCTATATTTATTTACTTTAGTTTGTTGTTTAGTTTATTTATGTGATAAACTTTTTTATTTAACTCTGCGGCATGGTAAATAAGGTATGGTAATTTAACAAAAAAAACGATTTCATTGAATGAAGTTTAGAATATTTTAATATAGAAAACATAAGAAATTGGTTAATTATGAAAGGAAATAATAAGAAAATTCCTTACTTTTAGCCCACAGTGCACTAAATAAGGTCCCACTAAAAAACTATCGATATATTGATAGCAGCATTTCCTTCCCGATAATACCCTAAATCGCGAGAAACGACGAGAAGCTTCGATGAGACCGAAAAAATCTATAAAAGGCGTGCGCATAGATTTTTT

TTCTCAAACTCACAAAATTTCTCAGCAAAAGAAAGCAAGTCGAAAGAAGGCGAGCCACGC

-180 -161 4

-89 -45 9

>hsp40_D_pseudoobscura

TATTCCACAGGAGTGCATTTGTATCCTTTTCACTCATTCATTTTCAAGCAATTCCACTTTGGCGCGCAGTCGATAGTATCGATAGCCGTTTCTGTGAATTAATCATACTTAGGTTGACTCTTTTGCTTGAACCTTTTTTAGACACAGTCAAAAAAAGAAAACATCCTTAAATAGGACCATTCTTGAACAAAATTGATTCATCTATCGAAGAAAATTATGTCCTTAGGGCTGACAGTCCTAGATAGCTGATAATATTCAGCCGAATATCAAAATCGAGGAATACGATTTCCGATTATGGACAGAGAACGATTAACCCATTGTATTAAAATAAGCTCCACGAAAAAAATTAAATTCGAAGAATTTAGCGCAGTTCCGGTAAAAAATTTGGTAGTCATTATTTTTGTGTAAGAAAAAAAATATAAATATATTTACAAAGTATTTCCAAACAGTAGTATTTGCCATGGGTTACTGTAGCTGTCCACATAATTATATGAATGGGGGGCCTAAGTGGGTTAAGTTCTCCAAGCAGCGAGAAACATCGAGAAAATTCGACGAGCAGACGGAACAGAGTCTATATAAAGGCCTGCGATATTCGGGCGA

CAAACAAAAAAATTTTATCGACTGAGCAACAAGGGTGTGTGCTAACACAGCAAACGAGAA

-427 -413 3

-84 -50 7

>hsp40_D_persimilis

ATTCCACAGGAGTGCATTTGTATCCTTTTCACTCATTCATTTTCAAGCAATTCCACTTTGGCGCGCAGTCGATAGTATCGATAGCCGTTTCTTTGAATTAATCGTACTTAGGTTGACTCTTTTGCTTGAACCTTTTTTAGACACAGTCAAAAAAAGAAAACATCCTTAAATAGGACCATTCTTGAACAAAATTTATTCATCTATCGAAGAAAATTATGTCCTTAGGGCTGACAGTCCTAGATAGCTGATAATATTCAGCCGAATATCAAAATCGAGGAATACGATTTCCGATTATGGACAGAGAACGATTAACCCATTGTATTAAAATAAGCTCCACGAAAAAAATTAAATTCGAAGAATTTAGCGCAGTTCCGGTGAAAGATTTAGTAGTCATTATTTTTGTGTAAGTAAAAAAATATATAAATATATATAAAAAGTATTTCCAAACAGTAGTATTTGCCATGGGTTACTCAAGCTGTCCACATAGTTATATGAAGGGGGGCCTAAGTGGGTTAAGTTCTCCAAGCAGCGAGAAACATCGAGAAAATTCGACGAGCAGACGGAACAGAGTCTATATAAAGGCCTGCGATATTCGGGCGA

CAAACAAAAAAATTTTATCGACTGAGCAACAAGGGTGTGTGCTAACACAGCAAACGAGAA

-428 -414 3

-84 -50 7

>hsp40_D_willistoni

CTTAGGTTTGACACGATTGGACATTTTCTACAACATGTCCAAAACGGAAAACAACAAAAACAAACCACAGAATTATTTGGTACACGTAGATAAGCAGCAATTTACGCGGCGAAATGTAAATGTAAAGAAGTCGTTACATAGCTATCGATAAAATGTAGATAACTAAAATTGACAGTGGCCAATAAATTTGATAAGAAAAAACAAATGTATCCTGTTCCGTTTGGAACAAATAGTAACAAATAGTAAATCATATATGTATTATTATACAATACGACTGTTAAAATAAATATCGATATCGAACATGAATTTATCGATAAGTAACTAAAAAGGATTTTTAAAATGCTTTTTCTTGTTTATAAACAAAATTTTAAAATATTTATTCCTAAACAAATGACAAAGCCGTAACTTTAATAGCGAATTTCACTATGAATTCCCTCGCAATGTAAAGTTATTCACTATTGATTGCCATTCAAAAACACGCGCTTAATTTTGAGTGACTATCGTTTCCAGATGCATCCCTGCGCATCTGGAACGTTCAAGAACATTCGACGAGCAGACGCACCCGAAACTATAAAAGAGGCGAGCGCTGGGAATTTCGCT

TCAATATCAAACAACAAAACATACCGAGAACAAGGTGGGCGTGTGAAAACACAATTTTCT

-97 -53 9

>hsp40_D_mojavensis

ACATATGTATGATTTCACTTACCTGAACTGTGAAAATGTTGCAATATTGTGTTTTGGATAATGCACAATCCGGCCCTACGGCTGGGAGTACACTGTTCGTCTAATTTGATACTATCTAAACTGTTTAAACGGAGGATGCTTGATGTTGACACCGAGAAGCAACCATTTTTATAATTGAACCAAATGTTTTTTGTTGTTCGGCTGCGGTTTGGCTGCAGTTCTATCGATGATAGTACCGATAGCCACACATACTGTCGAATATATATTCGATAGCTCAAACGTCAGTTGACGAAGAGTATCGATGCTACGGCTGCAATTCGACCATCCAAGTATCGATAATTGGTATTTAAAAAACATGGTCAATTTATTAATCCCATCATGTGTAATCGATGCTTAGTTAAAAATAATTTATTAACTAAAATATCTTTATTTTTATTCAAAAAATTCTTTACAATTTATTCAAAAAATTATATTTTGGCGCCAATTGTTATCGAGATGCCAGTTTCCGGATGCAACCCTACTTTGCTAGAAGCATCGAGAATTTTCGACGAGCAGACTGCTCCGAGTCTATATAAAGGCAAGGCACGCTGTCATTTCGAC

TCATTTAGCAAAAATCAGAGAAACGTATAACTAGTGTGTAGTTCACACAAGAGAGAAATA

-98 -54 9

>hsp40_D_virilis

ATACATATGTATGATTTCACTCACCTGAACTGTGAAAATGTTGCAATATTGTGTTAGGGTTAAATGCACAATCCGGCCCAAGGGCTGGGACTACACAGGCCAATTGATTTGTTGCTATCTAAACTGTTTAAACGGAGGATGCTTGACGTTGATACCGAGCAGCAACCATTTTTATAAATGAAACAAATGTTTTTTGTTGTTCTGCTGCGGTTTGGCTGCAGTTCTATCGATGGCAATACCGATAAGCCTGCCTGCTATCGATGGTTATCCGATAGTTCCATCAGTCACCCTACACAAAGTATCGATGCTACGGCTGCAATTCGATTCAAATCAAATCGATTATTGATTTGTAACAAACGCAGTTAATTGACACACTATAATCCACAAATCAATAACCCATGCTTATTTAAATTCAATTTATCAACTAACATGCAATTTTTATGTTCTTAGCAAAATGATTTAAATATTTGAATTTTGGCGCTATATGTTATCGAACCGTTGTGTTCCGGATGCAACCCTATTATGCTAGAAGCATCGAGAATTTTCGACGAGCAGACTGCTCCGAGTCTATATAAAGGCAACATCCGTTGCCAGTTCGAC

TCATTTAGCAAAAATCAAGAGAAACGAGTAGTGGGTGTGTTTTCACACTAGATAAAAAAA

-98 -54 9

>hsp40_D_grimshawi

ATGTATGTATGATTTCACTTACCTGAACTGTGAAAATGTTGCAATATTGTGTGTGGGATTTATACACAATCCAGCCCAATGTCTGGGCGAACACAGGGCTTTCGATTTAATGCTATCTAAATTAGTTAAACGGAGGATGGTTGATGTTGGTACCGAGCAGCAACCATTTTTCTAAATGAAACAAATGTTTTTTGTTGTTCGGCTGCGGTTTGGCTGCACTTCTATCGATGACACTACCGATAGGCAACTCCTGCTCTCGATGGTATATCGGTAGTTGAAGTAGATTATTCACGAGAAGCATCGATGCTAGAGTTGTAATTCGATTGCGGCATAATCGATTTTTTTTATATGTTAAAAACACTTTAAATTCGACAAATCAAATACTTCAAATACGTAAAATGTCTATTGCTTGTGAATTACTCAACTAAAATTCAATTGGTTGATTACTAGATAAATTTTGTGTTAATTTTAATTCTGGCGCCCTTTGTTGTCGGAACGCAATATTCCGGATGCAACCCTACCTTACGAGAAGCATCGAGAATTTTCGACAAGCAGACTGCCCGAAGTCTATATAAGGGTGACGTACGCTACCACTTCGAT

TTATTTAGCAAAAATTAAGACGAGTGTTCAGAGGGTGTGTACCCAAAACAAGAGAGAACC

-319 -305 3

-98 -54 9

>hsp68_D_melanogaster

TTAAAATTCCCTATTTATAAACACATTAAAGATTGTATCGATCTACGAAGTATTCTATATACATCCTATCTATGTACATCCTATATATGTATCTATATATTTATGTATCCTGGATATAGAGCAGTACGATTCTTTAAATTTTTGGAAATAATTAATTGGAATAATTAAGTTTTGAAAAGTGTGTTCAACTTTTAAGGTAATTGTATGTCATATTCTGCAGAGGAAATATTCCAATCAGAAGAAAAATACACACATATGTATTGCACATATAATAAAAGAGATATAAACCAAAAACAGAATCTAAAAATATGAATGGCTTAACGTATCAATCCGCAAATCAGCCAAAAATAGCTTGCATACGAATTTAGTTTAATGACCACTTGGAATGATGACCTATGTCAATAAAAAAAATATATTTCTCGAAATTTCCTGAACTTTCAGTCCTTTCAACTGACAATTTCCCCTGCGAAGTGCACACTAGTTGACTCGCTCGCACACACACGAACTGACTGGAATGTTCTGACCCTTTCTCGCAGGGAAATCTCGAATTTTCCCCTCCCGGCGACTGAGTATAAATACGGGCGCAAATTTCCCAGACGC

TACATTTGAAATCAAACAGCCAAAGTGAAAACACTTCAGCAAACGAAAAATAAAAAGCAG

-185 -161 5

-99 -65 7

-61 -47 3

>hsp68_D_simulans

ATATTAATAATGCACACTTGGCATTTGGTCTTGCATCTTAAAATTCGCTATTTATAAACACATTAAAGATTGTGCCGATCTACGAATTATTCTATATATCCTGTATTTATATCAGCACGATTCTTTAAATTTGTGGAATTAAATAATTGAAATAATTAGGTTTCGAAAAGTGTGTTCAACTTTTAATCTAATTGTGTGTCATATTCTGCAGAGGAAATATTCCAATTAGAAGAAAAATACATATGTGTATTGGAATATGCACATTTAATTATAGAGACATAAAACAAAAACAGAATCTAAAAATATGAATGGCTTAACGTATCAATACGCAAATCAGCCAAAAATAGCTTGCATACGAATTTAGTTTAATGGCCACTTAAAGTGCTGACCTATGGCAATAAAAAAAATATATGTAGCTCTCGAATTTTCCTGAACTTTTACTCCTTTCAACTGACAATTTCCCCTGCGAAGTACACACTAGTTGACTCGCTCGCACACACACGAACTGACTCGAATGTTCTGGCCCTTTCTCGCAGGGAAATCTCGAATTTTCCCCTCCCGGCGATTGAGTATAAATAGCGGCGCAAATTTCCCAGTCGC

TTCATTTGAAATCAAACAGCCAAAGTGAAAACACTTCAGCAAACGAAAAATAAAAAAAAA

-185 -166 4

-99 -65 7

-61 -47 3

>hsp68_D_sechellia

TCTATGCACACTGAGATATTTGGCATTTGCTCTTGCATCTTAAAATTCGCTATTTATAAACACATTAAAGATTGTGCCGATCTACGAATTATTCTATATATCCTGTATTTATAGCAGCACGTTTCTTTAAATTTGTGGAATTAAATAATTGAAATAATTAGGTTTCGAAAAGTGTGTTCAACTTTTAATCTAATTGTGTGTCATATTCTGCAGAGGAAATATTCCAATTAGAAGAAAAATACATATGTGTATTGGAATATGCACATTTAATTATAGAGACATAAAACAAAAATAGAATCTAAAAATATGAATGGCGTAACGTATCAATACGCAAATCAGCCAAAAATAGCTTGCATACGAATTTAGTTTAATGGCCACTTAAAGTGCTGACCTATGACAATAAAAAAAAAATATTGCTCTCGAATTTTCCTGAACTTTTACTCCTTTCAACTGACAATTTTCCCTGCGAAGTACACACTAGTTGACTCGCTCGCACACACACGAACTGACTGGAATGTTCTGGCCCTTTCTCGCAGGGAAATCTCGAATTTTCCCCTCCCGGCGATTGGGTATAAATAGCGGCGCAAATTTCCCAGACGC

TACATTTGAAATCAAACAGCCAAAGTGAAAACACTTCAGCAAACGAAAAATAAAAAAAAC

-185 -166 4

-99 -65 7

-61 -47 3

>hsp68_D_yakuba

GTCTTTATGTAAAGATTTATATACATATGTATATATGTTATAAACTCAAATAAAACACAAAATATAAAATTAAGATCTAAATATTCCATATATCCTGGTTATGTCAGTCCGACTTCTAAGAATAGTACTTTCCTAGTTAATCTATAAAGTTGGATACGATCAACTTTTAAAAGTACAATTAACATTTAAGCTAATTGTATGTCTTTTCTGCAGGGGAAATATTCGAATCAGAAGAAATATATATGCGTATGGTAGATGTCCATGCACATATAATTATAGAGACGTCAATAAACCAAATAAACAGAATCTAAAAATATGAATGGCTTAATGTATTAATACGTTAGTCAGCCAATAATAGCTTGCATACGAATTTAGTTTTATGGCCACTTGGAATGCTGACCTATGGCAATAAAAAATATATCCCTCGATTGTTCCTGAACTTTCTACTCCTTTCAACTGACAATTTCCCCTGCGAAGTGCACACTAGTTGACTCGCTCGCACTACTGGAATGTTCTGGCCTTTTCTCGCAGGGAAATCTCGAATTTTCCCCTCCCGTCGAATGAGTATAAATAGCGGCGCAAATTTCCCAGCCGCTACAT

TTGAAATCAAACAGCCAAAGTGAAAACACTTCAGCAAACAAAAAGTAAAGAAAACATTTC

-180 -156 5

-99 -70 6

-66 -52 3

>hsp68_D_santomea

CATATGTATATATGTTATAAACTCAAATAAAACACAACAAATAAAATAAAGATCGAAATATTCCTGGTTATGTCAGTCGGACTTCTAAGAACAGTTCTTTCCTAGTTAATCTATAAAGTTGGATACAATTACGATTTTAAAAGTACGATCAACTTTTAAAAGTACAATTAACATTTAAGCTAATTGTATRTCTATTCTGCAGGGGAAATATTCCAATCAGAAGAAATATATATGCGTATGGTACATGTTCATGCACATATAATTATAGAGACGTCAATAAACCAAATAAACAGAATCTAAAAATATGAATGGCTTAATGTATTAATACGTTAGTCAGCTAATAATAGCTTGCATATGAATTTAGTTTTATGGCCACTTGGAATGCTGACCTATGGCAATAAAAAATATATCCCTCGATTGTTCCTGAACTTTCTACTCCTTTCAACTGACAATTTCCCCTGCGAAGTGCAGACTAGTTGACTCGCTCGCACCCAGACGAACTGACTGGAATGTTCTGGCCCTTTCTCGCAGGGAAATCTCGAATTTTCCCCTCCCGGCGACTGAGTATAAATAGCGGCGCAAATTTCCCAGCCGCTANNN

-191 -167 5

-99 -70 6

-66 -52 3

>hsp68_D_erecta

AGAAACATTTAGAGGTATGCCACTTCAAAATCAGATTAAAATAAGTTGAATTATTGAGTAAGCTCTATGTACATACACGTTCTATTTAAACATGATATAGCCGTGTTTGTATTTTCTAGAAATTTGAAACAATTACGATTTTAAAAGTACGATCAACTAACTAATTGTATGTACATACATATGACTATTCTGCAGGGGAAATATTCCAAACAGAAGAAACATACATATATGAGTATGGTACATATTCTTGCACATGTACATATGTAGCTATAGAGACGTCAATAAGCCAAATAAACAAAATCTAAAAATATGAATGGCTTAAGTATTAATACGTTAATCAGCTAAAAATAGCTTTCATACGAATTTAGTTTTTTTGCCCACTTAGAATGCTGACCTATGACAATAAAAAAATATATCCCTCAATTGTTCCTGAACTTTCTACTCCTTTAACTGACAATTTCCCCTGCAAAGTGCACACTAGTTAACTCGCTCGCACTTAGGCGAACTGACTGGAATGTTCTGGCCTTTTCTCGCAGGGAAATCTCGAATTTTCCCCTCCTGGCGACTGAGTATAAATAGCGGCGCAAATTTCCCAACCGC

TACATTTGAAATCAAACAGCCAAAGTGAAAACACTTCAGCAAACGAAAAATAAAGCTTGC

-185 -161 5

-99 -65 7

-61 -47 3

>hsp68_D_ananassae

TCTATCATTCTGCATTATAATCTAGAAACGAAATATTTTTTAGATTTTTTGTTACATTTTCTGGGGGACCCCCTTCAAATGTGGCGAGAAATGCATGGAAGGACAATATGGCCCTCAGCGTTGGCTATATCTTGGCCAATTTTCATCCGATTCTTGAGCGGAATCCCTAAAACGGTTATTGATTGCTTGAACTACTGATTGCTTTCTTATTTTTTTGTATTGAGAACGAAAAGAAACACTTTCCAAAGTATAAATACAAATATTTTTGTTTAGTTTTCCAGTTGTGAGAAAATTGAAAACTGTTGTTCTTTGATTCAATTTTAAACACACGCCAATAAGTAAAATAAACATTGTCTAAAAATACTAATGGCTTAATCATCAATATGCGAATACGCCAAAAATAAATATAATTTTTAATAATCGAAATGCCGACCTGCGACAACCAAAACTACCCCTCAATTGTTCCTGAATTTTCCATTTTTCTCCGCTGCTGTCTCACTCGCTCTCATAGGAAACGTCGTGAGACTTCGAGAAGGGAAATCTCGAATTTTCCCCGCCCGAGCAGCCGAGTATAAATAGCGGCGGAAATTTCCCAGCCAG

CACATTTGAAAAGTGAAAAGCAAGTGAAAAGTTTGAAAGAAAGACAGCTGAAAAAGCTAA

-149 -125 5

-90 -66 5

-62 -48 3

>hsp68_D_pseudoobscura_A

CTGTTAGATCTCACTATAAAACGTATATCTCAAAATTTCTCCCCACCCTGTCTCCCGCACGCACCCATTGTCGCTTAATGTAGCCATACTGACTATGTATCGGGTATATGTAAATGTAGATTTGCGGTCGCCGCAGCAACTCACAACGTTCCCCCTTGTTTTGTTTTATGGCCATGTTTCCCTTTTTCATCACTTTTTCTCTAACATATTGAACTGTCTAAAAAAATCTCAGAAAATGTGCTCAATATCGGTTAACGGTTTTGATGAAAAATTCGCCTAATTTTTAAAAGTAGACTGGTTAATATATACCAGGCAGCACTGACCTACGTTAACGCTTCCCCTTAATTATTCTTGAATTTTCCATACCCTACAGTTCACATAATTTCCCCTTACACATGCTCTTGTTATCTCTCTCGCACTCTCTCGTAGCAAAGCGAACGTGTTTCACTTTTCACAACCTTCCCCTCTTTCGACACTACGGTTATCTCACTCGCACTCTCGCGAAACTTCGAGAATCATCGCGAACGTTCTCCCCTCTTCTGGCAAGGGAATCTCAAACTGGCCGGGAGTATAAATAGCGGCGCCGTTCGCTACCCCTGC

ACATTTCAATTCAAGAAAGCAACAACACAACAAGCAAAGCAAGCGAAAGTGACGGTTACA

-253 -239 3

-109 -55 11

>hsp68_D_pseudoobscura_B

ATCTGTACATATGTGTGTACACATACATATTCGTTTTTGTCAACTCTAAAAGAGGAGAAATTAAATAGGAATACCTCTTGCATTTATCAGCTAAATTTAGAAGACAACATATACATATCAATATTTTATAATAACAAATAGGTACCACCTGCTAAAAAAAGCTTATAAACAATATTATTTAGACGTCAATAAGCTGAATAAACAGTTTTAGAATATTACCAGTTCTGCACGTACACAAATATTGGTATATAGAGAGAATCTATAAATAAGCCAAATTGTTAAAAATAGACTGAATTAATAGATAAGTGGCAGCGCTGACCTACGATAATGCATCGCTCCCCTTAATTATTCTTGAATTTTCCATACCCTACAGTTCACATAATTTCCCCTTACACATGCTCTTGTTATCTCTCTCGCACTCTCTCGTAGCAAAGCGAACGTGTTTCACTTTTCACAACCTTCCCCTCTTTCGACACTACGGTTATCTCACTCGCACTCTCGCGAAACTTCGAGAATCATCGCGAACGTTCTCCCCTCTTCTGGCAAGGGAATCTCAAACTGGCCGGGAGTATAAATAGCGGCGCCGTTCGCTACCCCTGC

ACATTTCAATTCAAGAAAGCAACAACACAACAAGCAAAGCAAGCGAAAGTGACGGTTACA

-253 -239 3

-109 -55 11

>hsp68_D_persimilis_A

ATATGTATGTGTGTACACATACATATCGGTTTGGTCAACTCTAAAAGAGGAGAAATTAAATAGAAATACCTTTACATTTATCAGCTACATTTAGAAGACAACATATATGTACATACATATCAATATTTTATAATAATAAATAGGTACCACCTGCTAAAAAAAGCTTATAAATAATATTATTTAGACGTCAATAAGCTGAATGAACAATTTTAGAATATGACCAGTTCTGCAGGTACACAAATATTGGTGAAAGAATCTATAAATAAGCCAAAAATTGTTAAAAATAGACTGAATTAATAGATAAGTGGCAGCGCTGACCTACGATAATGCATCGCTTCCACTTAATTATTCTTGAATTTTCCATACCCTACAGTTCACATCATTTCCCCGTACACATGCTCTTGTTATCTCTCTCGCACTCTCTCGTAGCATAGCGAACGTTTTTCACTTTTCACAACATTCCCCTCTTTCGACACTACGGTTATCTCACTCGCACTCTCGCGAAACTTCGCGAATCATCGCGAACGTTCTCCCCTCTTCTGGCAAGGGAATCTCAAACTGGCCGGGAGTATAAATAGCGGCGCCGTTCGCTACCCCTGC

ACATTTCAATTCAAGCAAGCAACAACACAACAAGCAAAGCAAGCGAAAGTGACGGTTACA

-253 -239 3

-109 -55 11

>hsp68_D_persimilis_B

GATCGGATCATTTTTATAGCCAAAAGGAACAAATTATTTTGCAGTGGCTATGCAGCGCCCGACGACATGTTCAGACACGTTTTCTGTCTCCCGCACGCACACATTGTCGCTTAATGTAGCCATACTGACTATGTATCGGGTATATCGGTCACCGCAGCAACCTAACCTTTACCTTTTTCCTCACTTTTTCTCTAACATATCGAACTATCTAAAAAAATCTCAGAAAATCTGCTCAATATCGGTTAACGGTTTTGATGATAAATTAGCCTAATTTTTAAAAGTAGACTGGTGCGGCTGTTTAATATATGCGAGGCAGCACTGACCTACGTTAACGCTTCCCCTTAATTATTCTTGAATTTTCCATACCCTACAGTTCACATCATTTCCCCGTACACATGCTCTTGTTATCTCTCTCGCACTCTCTCGTAGCATAGCGAACGTTTTTCACTTTTCACAACATTCCCCTCTTTCGACACTACGGTTATCTCACTCGCACTCTCGCGAAACTTCGCGAATCATCGCGAACGTTCTCCCCTCTTCTGGCAAGGGAATCTCAAACTGGCCGGGAGTATAAATAGCGGCGCCGTTCGCTACCCCTGC

ACATTTCAATTCAAGCAAGCAACAACACAACAAGCAAAGCAAGCGAAAGTGACGGTTACA

-253 -239 3

-109 -55 11

>hsp68_D_mojavensis

TTTTGGTAGCATATACACACAAACAATCTATGTGCCAGAGCAATTTGACAACCCAGAAAGTATTTTCCAGAGTACTAAAGATATAAATACATACATATGTACATATGTATGATATACTTATGTATGTATGATATTAATATATATAGGATTAGATTAGGCAAACTTTCTGGTTGATCTGATTTACAAATTACAAGAGCAAACAGAGAATAACCCAATTGCAAAGTTTTTAAATTATTAAAGAACAAACAAAAATGCAATAAGTACTTTTATTTAATCTAACCATACAAAATCTGTTCATGCGAGCATTAAAAATTTAGACTCTAAATTTTATAGTTGTTAACAAATTATTGAGCAAGGTAAGTTTTGACTGAGTGGGTCAATCGACTGTCAAACCAATAACAAACGCTTGACTTTATATATTCGACATGACTGTTTAATGTCATTCCCCTTCGGCATTACACACACACATACACATTTTATACGAATCGCACTGTCGACAGTGTCGAATTGCCTCGAACATTCTTCTCTCTTTGAGGGGAATCTCGAAATTTCCACCACTGACCGGCTGAGTATAAATAGCAGTGCGCATTTCCACCTCAT

CACAGTTCATAGCAAGCAATTGTAGTGAAAACACAACAAGCGAAACAAAGAAAAGCTGAA

-97 -78 4

-62 -48 3

>hsp68_D_virilis_A

TAAACAAATTGAAATATCGAAGAGGTTAATGTCGCGATGCAGCTCTCCGTCGAAAAGTCACGAAAGAAAACTTATTTTCACTACGAATATAAAGTTCACTTGTACCGCATTACCCAAAATGCCGCCAAAATATTTACCAACAACGATTGGCATGGGGAAAGATTAATTGCGCATTTTGTTTGACGTAGTACGGCTGCGCAACTGGAAGACGCAACATTCCAATTTCTCAGGGGAGCAGGGCAGAGCAAATTTTCGAAACGCATTAAGGCTCGTTGCACTAAATAAATAATATCATATTTATTCTCAAAAAAATCAAATGTCGTTGATTTCATACGCACTGCCAGTCGAAACTCAAGCAAATAATTGGCAGAAGGCATTTGCCGCGCATTATATATTGGAAGTCGTGCGTGTTAAAAGCAGTCGTTCCCCTTCACACAACAAACACACCACATACATACATATACGCCTATAAAAAACACTGTCTGGCTCGCTCGCACTCTCTCGAATCGTCTGGAGTCAGTACTCTTCTATTGAGGGGAATCTCGAATATTCCACCACACAGCGTCAGAGTATAAATAGCAGAGAAAATTTTCAGCTCGT

CACATTTCATAGCAAGCAGTCGTAGTGGAAACATCAAGCGAACCAAAGAGAAGCTAAACA

-108 -84 5

-62 -48 3

>hsp68_D_virilis_B

GAATTTAATGATAAATGCTATTACATATGTATGTATGTATATTATTCCATTGGTAAGGCTGCCATACCGTTTCCCTTTTAGAGAGTTGCCAAACTTTTTTTTTCCGTATTTCGGATAAATTAAAACAAAAATAGCTTGACACCGGCAAACGCAAGAATGGGGATTTCTAGTTCAATTCACTCGTTTATTTTCTGGTCATTAACTAAATCGGTCATTTAGTTCGCTGGTTTATTCGGTCATGATATATACTTACTGAACAACTGAACATCGGAATAGAATATCGTTAATGAACGAGTGAACGAGTATGGATTATTATTTTGCTCTTATTTCCCTTTCTGCGCTTTCTCTGAACAGAGGATATAAATGTGATATGATATGTGTAAATATATATGCATATGTATGCACACTTTATAGTTTATTTCTAGCTAGAAAAAGGCTTACATGGCTGCGCTGACGTTTATCTGTGAACATACAGACGAGTAATGTCTGGCAGTCTCTCGAATCGTCTCGAACCTTCTGCACTCTTCTTTTGAGGGGAATCTCGAATATTCCACCACACAGCGTCAGAGTATAAATAGCAGAGCAAATTTTCACCTCGTC

ACAATTCATAACAAGCTATCGTAGTGGAAACACAGCAAGCGAACCAAAGAGAAGCTAAAC

-122 -73 10

-63 -49 3

>hsp68_D_grimshawi_A

CGATTGGCTTTTAACTGAATTATCCATATTTGTGTAGTAGTTTTGTCTCTCATCGGTATCCTTAGTGTCTACGCCGATCTCGATCCCACAACTTAAAACTTCTCGGTTTTCCTAAGCAAGCTATATAATATAGTGATCCGGTCCGGTCGCCTCAAAAAGCGAAGGCAAAAGAACAGATTTATAGTTTCACCAAGTTATATAAGCAAACAAAATACATCAAATTAATTTACTTAAGAAAAAACTGGTTTATTACAGCCCACTCAGCATATACAAATAAACATAATACGTATGGATTTCTGGCTTAAATATTTCTATTGAAAATTATTGTCTGGTAAAACGAGATTTGCGATTTAAGCAAATATTCAACTGCAAACCAGATTCCATTTAAGTTGGCATTAAATATTGGACTGGTCAGTTTTTGAAAATAAACATTCCCCTTTACGCATACCCATTCCACAAGCAGTCGAACACACTCTATGTGCCTCACTCGCACTTTCCTGAATCGTCTCGAACATTCTGCACTGTTCTTGTAAGGGGTATCTCGAAATTTCCACCACTCGGTATCTGAGTATAAATAGCAGAGCAAATTTCCACCACGGC

ACAGTTGCAAACAAGCAATTATTGTGAATACACAAAAAGCTAACACTTGGAATAAGAAAA

-112 -68 9

-63 -49 3

>hsp68_D_grimshawi_B

ATTTGCTAGGGTTAGTAATCTTGGGTGTGTCCTTGTTTATTTATTTATCTACTTCAGTTTCAACTTTTAGAATCAGCCCTTCACTTACTTCACTTGGTGCTCTGAAGAGTCCATACCATTGCGGACTCATGGTGGCGAAATCTTTATTTATTATACAACTCGGGGAAATAAGCTACACAATGGACTAATCAATACATCGAAGCGTCCTCAAATTTTCTGCTAATAGCACTACGTAGAATGCTTAATGGCCACTAAAGAAATTAATGTGTTTTTTATGCATTTGGCTTAAAGATACTTTTACCACCCACGACATGACGTAGAAATTCGTAATAAACCAATAATAGAATTTCTATTTTCAATTGAATTTCTACTTCTACTTCTATTTATATTTTGTATTTTTACTTTTATGCTAAAAATAATTCGTTTCCATATGTTTATGAATTGTATGTACATATATATAATTTATACTAATGTATGTATCTCGCTCTCGCACTTTTCTGAAGCGTCTTGAACATTCTGCACTGTTCTTGTAAGGGGTATCTCAAAATTTCCACCACTCGGTATTTGAGTATAAATAGCAGAGCAAAATTCCACCACGGC

ACAGTTGCAAACAAGCAGTTATTGCGAATACACAAAAAGTGAACTGGAAAAGAGAAGCTGA

-112 -68 9

-63 -49 3

>Hsp70_D_melanogaster_Aa

ATATTGAATAAAAAAATTGCGTTTAATTAAGCAAAGAAACCTTCATATTTACCTTTAAAGCGAAAAATTCAACTTATTTCACAGTGTAAACAGTTGACAACAACAGTCTTGACAACCTTTACGTAATTTTAAATAAAAAACACTAACAATCATCTGCATGCAATTGTCTGTATTAATCTAATAAATAAATAGCTTTTTTAAGTTAGTATGTAAATACATTTTTAAGAATATCTTGTCAAAGTTCCATAGGCCTTTCTGGCGGACAACATCCGCGTAACAAACCCTTCGATTATCTCTAACATAATTAACTTAAGCAGCCGTATTTATAAAGAAATTTCCAAAATAAAGCGAATATTCTAGAATCCCAAAACAAACTGGTTGTTGCGGTAGGTCATTTGTTTGGCAGAAAGAAAACTCGAGAAATTTCTCTGGCCGTTATTCTCTATTCGTTTTGTGACTCTCCCTCTTTGTACTATTGCTCTCTCACTCTGTCACACAGTAAACGGCGCACTGTTCTCGTTGCTTCGAGAGAGCGCGCCTCGAATGTTCGCGAAAAGAGCGCCGGAGTATAAATAGAGGAGCTTCGTCGACGGAGAGTCA

ATTCTATTCAAACAAGTAAAGTGAACACATCGCTAAGCGAAAGCTAAGCAAACAAACAA

-252 -238 3

-192 -173 4

-88 -69 4

-65 -46 4

>Hsp70_D_melanogaster_Ab

TAAACTTGAAATTTGTTGTTTATTTTCTTGTATATTTGAAATTTTGAATTTGTTTTTTAATTTATAAGTAAAATAAATATTGTTTAATCATATTTCATAAAAAAATTGCGTTTAATTAAACAAATAACCCCTTATTTTGACCTTTAAAGTCAAAAATTCAACTTATTTCACGGTGTGTAAACAGTTTCTTGACAAACTGTATGCCGAATGGGCATTTATTGGTTTATTGGATTGGCTGCGCCGAAAAATAAGCCATAGTCGGCACCATAAGCATAACCTAGCTCTGCGATTATCTCTAACATAATTAACTTAAGCAGCCGTATTTATAAAGAAATTTCCAAAATAAAGCGAATATTCTAGAATCCCAAAACAAACTGGTTGTTGCGGTAGGTCATTTGTTTGGCAGAAAGAAAACTCGAGAAATTTCTCTGGCCGTTATTCTCTATTCGTTTTGTGACTCTCCCTCTTTGTACTATTGCTCTCTCACTCTGTCACACAGTAAACGGCGCACTGTTCTCGTTGCTTCGAGAGAGCGCGCCTCGAATGTTCGCGAAAAGAGCGCCGGAGTATAAATAGAGGAGCTTCGTCGACGGAGAGTCA

ATTCTATTCAAACAAGCAAAGTGAACACATCGCTAAGCGAAAGCTAAGCAAACAAACAAG

-252 -238 3

-192 -173 4

-88 -69 4

-65 -46 4

>Hsp70_D_melanogaster_Ba

ACATTACATAGCTTTCATAAAAGCCTCGGCTTTTGCATATGTTTTGCATGCATATGTAATACATAATATATATGTACATACATATATCATATTCATAACATATATAATTTTAAATTAAAAAAGCAAGCAATCATCATGCAATTGTCTGTATAAATCAAATATATATATAAATAATAAATTTGGCTTTTTTAAGTTAATATATACATACATTTTAAAGAGTATATTGTCAATGTTACATAGGCCTTTCTGACGGACAACAACCGCATAACAAAGCGCTTCGATTATCTTTAACATAAGTTTTTAAGCAGCCGTATTTATAAAGAAATTTCCAAAATAAAGCGAATATTCTAGAATCCCAAAACAAACTGGTTATTGTGGTAGGTCATTTGTTTGGCAGAAAGAAAACTCGAGAAATTTCTCTGGCCGTTATTCGTTATTCTCTCTTTTCTTTTTGGGTCTCTCCCTCTCTGCACTAATGCTCTCTCACTCTGTCACACAGTAAACGACATACTGCTCTCGTTGGTTCGAGAGAGCGCGCCTCGAATGTTCGCGAAAAGAGCGCAGCAGTATAAATAGAGGCGCTTCGTCTACGGAGCGACA

ATTCAATTTAAACAAGCAAAGTGAACACGTCGCTAAGCGAAAGCTAAGCAAATAAACAAG

-261 -247 3

-201 -182 4

-88 -69 4

-65 -46 4

>Hsp70_D_melanogaster_Bb

CAATTGGGAAATGTAATCTTTGCCAGAATGGTTACGGAGTTCAACAACAAAAACAACTATAGAAATAATAGCCTTTCCTTTCCTCATATGTATGTAAATATGTAAAATAAGTCGCAACTAAATTCTAATACACTTCTCAGTCTTAAATTAATTTTATCGTCTATTAAAACAGAAGAAAGTCCGTTAATCGTTGATTTCGTTAACTAAAAGTACAAAATAATCTTTAATCTTTAGAAACGCAGCAATGTTTTGGCGGCATACGCATAACAAAGCGCTTCGATTATCTTTATCATAAGTTATTTAAGCAGCCGTATTTATAAAGAAATTTCCAAAATAAAGCGAATATTCTAGAATCCCAAAACAAACTGGTTATTGTGGTAGGTCATTTGTTTGGCAGAAAGAAAACTCGAGAAATTTCTCTGGCCGTTATTCGTTATTGTCTCTTTTCTTTTTGGGTCTCTCCCTCTCTGCACTAATGCTCTCTCACTCTGTCACACAGTAAACGACATACTGCTCTCGTTGGTTCGAGAGAGCGCGCCTCGAATGTTCGCGAAAAGAGCGCCGGAGTATAAATAGAGGCGCTTCGTCTACGGAGCGACA

ATTCAATTCAAACAAGCAAAGTGAACACGTCGCTAAGCGAAAGCTAAGCAAATAAACAAG

-261 -247 3

-201 -182 4

-88 -69 4

-65 -46 4

>Hsp70_D_melanogaster_Bbb

CCTTTAAAGTCAAAAATTCAACATATTTCACAGTTTGTAAACTTGATAAACTGTATGTAATAGTCTTGGCTTTCCTCATATGTACATATGTATGTAAATATGTAAAATAAGTCGCAACTAAATTCTAATACACTTCTCAGTCTTAAATTAATTTTATCGTATATTAAAACAGAAGAAAGTCCGTTAATCGTTGATTTCGTTAACTAAAAGTACAAAATAATCTTTAATCTTTAGAAACGCAGCAATGTTTTGGCGGCATACGCATAACAAAGCGCTTCGATTATCTTTATCATAAGTTATTTAAGCAGCCGTATTTATAAAGAAATTTCCAAAATAAAGCGAATATTCTAGAATCCCAAAACAAACTGGTTATTGTGGTAGGTCATTTGTTTGGCAGAAAGAAAACTCGAGAAATTTCTCTGGCCGTTATTCGTTATTGTCTCTTTTCTTTTTGGGTCTCTCCCTCTCTGCACTAATGCTCTCTCACTCTGTCACACAGTAAACGACATACTGCTCTCGTTGGTTCGAGAGAGCGCGCCTCGAATGTTCGCGAAAAGAGCGCCGGAGTATAAATAGAGGCGCTTCGTCTACGGAGCGACA

ATTCAATTCAAACAAGCAAAGTGAACACGTCGCTAAGCGAAAGCTAAGCAAATAAACAAG

-261 -247 3

-201 -182 4

-88 -69 4

-65 -46 4

>Hsp70_D_melanogaster_Bc

CAATTGGGAAATGTAATCTTTGCCAGAATGGTTACGGAGTTCAACAACAAAAACAACTATAGAAATAATAGCCTTTCCTTTCCTCATATGTATGTAAATATGTAAAATAAGTCGCAACTAAATTCTAATACACTTCTCAGTCTTAAATTAATTTTATCGTCTATTAAAACAGAAGAAAGTCCGTTAATCGTTGATTTCGTTAACTAAAAGTACAAAATAATCTTTAATCTTTAGAAACGCAGCAATGTTTTGGCGGCATACGCATAACAAAGCGCTTCGATTATCTTTATCATAAGTTATTTAAGCAGCCGTATTTATAAAGAAATTTCCAAAATAAAGCGAATATTCTAGAATCCCAAAACAAACTGGTTATTGTGGTAGGTCATTTGTTTGGCAGAAAGAAAACTCGAGAAATTTCTCTGGCCGTTATTCGTTATTGTCTCTTTTCTTTTTGGGTCTCTCCCTCTCTGCACTAATGCTCTCTCACTCTGTCACACAGTAAACGACATACTGCTCTCGTTGGTTCGAGAGAGCGCGCCTCGAATGTTCGCGAAAAGAGCGCCGGAGTATAAATAGAGGCGCTTCGTCTACGGAGCGACA

ATTCAATTCAAACAAGCAAAGTGAACACGTCGCTAAGCGAAAGCTAAGCAAATAAACAAG

-261 -247 3

-201 -182 4

-88 -69 4

-65 -46 4

>Hsp70_D_simulans_A

CATGTAAATTTCGCGCTAAATTCTTGCAATAGAAAGATGATTAAATTTAGTTACATTTGTTTTCATAAAAGCATCTGTGTTTGCATATGATTTTGCATGCATATGTAATACAAACATACATAAACCTACATATGTGATAATTTAACGTATATGTATATGTATGTAATTTTAAATACAATAAACTAATCATATGCATCCAATTGTCTGATCAAACTAATAAATAAATGTCTTTTTTAAGTTCACAAATACATTTTGAAGCGTATTTTGTCAAAGTTGCATAGGTCTTATTGACGGGGACCATAAGTTATTTAAGCAGCCGTATTTATAAAGAAATTTCCAAAATAAAGCGAAAATTCTAGAATCCCAAAACAAACTGGTTGTTGCGGTAGGTCATTTGTTTGGCAGAAAGAAAACTCGAGAAATTTCTCTGGCCGTTATTCTCTCATCGTTATGTGGCTCTCCCTCTCTGCACTAATGCTCTCTCACTCTGACACACAGTAAGCGGCATACTGTTCTCGTTGCTTCGAGAGAGCGCGACTCGAATGTTCGCGAAAAGAGCGCCCGGAGTATAAATAGAGCCGCTTCGTTCCCAGAACGTCA

ATTCAATTCAAACAAGCAAAGTGAACACATCGCTAAGCGAAAGCTAAGCAAACAAACAAG

-253 -239 3

-193 -174 4

-89 -70 4

-66 -47 4

>Hsp70_D_simulans_B

AAATTGGTTTAAATGTAAATACTGCGCTAACATATGAGTGTAGGCCCATATCGCTTCTTGCAATAGAAAGATGACCCTTCGCAAAGATTAGTAATAAAGTTTAGTTGCATTACATTGTTTTCAAAAAAGCCTCCTCTGTTTTTGCATACGTTTTGCATCCATTTGTAAAACAAACATACATATGTACGTAAATCTATGTGATTTACAATCACCTGCATACAATTGTCCGTATCAAACTAATAAATAAATCGCATTCGCTATGTACATCCGCATAACAAAGCGCTTCGATTATCTTTAACATAAGTTATTTAAGCAGCCGTATTTATAAAGAAATTTCCAAAATAAAGCGAAATTTCTAGAATCCCAAAACAAACTGGTTGTTGTGGTAGGTCATTTGTTTGGCAGAAAGAAAACTCGAGAAATTTCTCTGGCCGTTATTCTCTCGTCGTTTTGTGGCTCTCCCTCTCTGCACTAATGCTCTCTCACTCTGACACACAGTAAACGGCATACTGTTCTCGTTGCTTCGAGAGAGCGCGCCTCGAATGTTCGCGAAAAGAGCGCCCGGAGTATAAATAGAGCCGCTTCGTTCCCAGAGCGTCA

ATTCAATTCAAACAAGCAAAGTGAACACATCGCTAAGCGAAAGCTAAGCAAACAAACAAG

-253 -239 3

-193 -174 4

-89 -70 4

-66 -47 4

>Hsp70_D_simulans_C

ATAGCATTTAGTTTCCAAAGCTGCATACAATGTTATGTATGTATGTACATAATTAATTTCAATGAATCTAGTACACATTTAGATGCTTACATATGTAAATTAAGTCGCAACTAATTTCTTATACAGTTTCCAGACCCGTAAAAGTTATATTATCGTATATTAAAACAAAAGAAAGTCCGTTAATAGTTGAGTTCATTAACTAAAAGGCCAAAATAGTCTTTAATACATATGCCGAGCAGACATTTATTGGTTTTAAAACGGTGGCATACGCACAACAAAGCGCTTCGATTATCTTTAACATTAGTTATTTAAGCAGCCGTATTTATAAAGAAATTTCCAAAATAAAGCGAAATTTCTAGAATCCCAAAACAAACTGGTTGTTGTGGTAGGTCATTTGTTTGGCAGAAAGAAAACTCGAGAAATTTCTCTGGCCGTTATTCTCTCGTCGTTTTGTGGCTCTCCCTCTCTGCACTAATGCTCTCTCACTCTGACACACAGTAAACGGCATACTGTTCTCGTTGCTTCGAGAGAGCGCGCCTCGAATGTTCGCGAAAAGAGCGCCCGGAGTATAAATAGAGCCGCTTCGTTCCCAGAGCGTCA

ATTCAATTCAAACAAGCAAAGTGAACACATCGCTAAGCGAAAGCTAAGCAAACAAACAAG

-253 -239 3

-193 -174 4

-89 -70 4

-66 -47 4

>Hsp70_D_sechellia_A

TATGTACTTACGTACATACATGTATATGTATGTAATTAAATTTTAATGAATCTAGTACACATGTAGTTGCCGTATTGAATGGAGCTATCCATATAAACAACATACAAACATATGTAAAATAAGTCGCAACAGACAGTTTCCAGACCCTTAAAGTTATGTTATCGTATATTAAAACAGAAGAAAGTCCGTTAATAGTTGAGTTCATTAACTTAAAGGCCAAAATAATCTTTAATGTATGCCGGAGCAGACATTTATTGGTTTAAAAACGAAGCAATTTTTTGGAGGCATGCGTAATAAAGCGCTGCGATTATCTTTAACAAAGGTTATTTAAACAGCCGTATTTATAAAGAAATTTTCTAGAATCCCAAAACAAACTGGTTGTTGCGGTAGGTCATTTGTTTGGCAGAAAGAAAACTCGCGAAATTTTTCTGGCCGTTATTCTCTCATCGTTATGTGGCTCTCCCTCTCTGCACTAATGCTCTCTCACTCTGACACACAGTAACGGCATACTGTTCTCGTTGCTTCGAGAGAGCGCGCCTCGAATGTTCGCGAAAAGAGCGCCCGGAGTATAAATAGAGCCGCTTCGTTCCCAGAGCGTCA

ATTCAATTCAAACAAGCAAAGTGAACACATCGCTAAGCGAAAGCTAAGCAAACAAACAAG

-247 -238 2

-192 -178 3

-89 -70 4

-66 -47 4

>Hsp70_D_sechellia_B

GTAAAATATGGGTGTAGGCCCATATCCCTTCTGGCAATAGAAAGATGACCCTTCGCAAAGATTAGTAATTAAGTTTTGTTGCATTACATTGTTTTCAAAAAAGCCTCCTCTGTTTTTGCATACGTTTTGCATCCATTTGTAAAACATACATAAATCTATGTGATTTACAATCATTCTCATACAATTGTCTGTATCAAGCTAATAAATTAATAGCTTTTTTAAGTTAATAAATACATTTTGAAGAGTATCTTGTCAAAGCTATATACATCCGCATAAAAAAGCGCTTCGATTATCTTTAACATAGGTTATTTAAGCAGCCGTATTTATAAAGAAATTTCCAAAATAAAGCGAATTTTCTAGAATCCCAAAACAAACTGGTTGTTGCGGTAGGTCATTTGTTTGGCAGAAAGAAAACTCGCGAAATTTTTCTGGCCGTTATTCTCTCATCGTTATGTGGCTCTCCCTCTCTGCACTAATGCTCTCTCACTCTGACACACAGTAACGGCATACTGTTCTCGTTGCTTCGAGAGAGCGCGCCTCGAATGTTCGCGAAAAGAGCGCCCGGAGTATAAATAGAGCCGCTTCGTTCCCAGAACGTCA

ATTCAATTCAAACAAGCAAAGTGAAAACATCGCTAAGCGAAAGCTAAGCAAACTAACAAG

-252 -238 3

-192 -178 3

-89 -70 4

-66 -47 4

>Hsp70_D_sechellia_C

AAATTGGTTTAAATTTAAATACTGCGCTAACATATGGGTGTAGGCCCATATCGCTTCTTACAATAGAAAGATGACCCTTCGCGAAGATTTAGTAATAAAGTTTAGTTGCATTACATTGTATTTAAAATAAGCCTCCTCTGTCTTTGCATACGTTTTTCATCCATTTGTAAAACAAACATTCATATGTACGTAAATCTATGTGATCTACAATCATCTGCATACAATTGTCTTTATCAAGCTGATAAATAAATGGCATACGCTAGGTACATTCGCATAACAAAGCGCTTCGATTATCTTTAACATAAGTTATTTAAGCAGCCGTATTTATAAAGAAATTTCCAAAATAAAGCGAAATTTCTAGAATCCCAAAACAAACTGGTTGTTGCGGTAGGTCATTTGTTTGGCAGAAAGAAAACTCGCGAAATTTTTCTGGCCGTTATTATCTCATCGTTTTGTGGCTCTCCCTCTCTGCACTAAAGCTCCCTCACTCTGTCACACAGTAACGGCATACTGTTCTCGTTGCTTCGAGAGAGCGCGCCTCGAATGTTCGCGAAAAGAGCGCCGGAGTATAAATAGAGCCGCTTCGTTCCCAGAGCGTCA

ATTCAATTCAAACAAGCAAAGTGAACACATCGCTAAGCGAAAGCTAAGCAAACAAACAAG

-251 -237 3

-191 -177 3

-88 -69 4

-65 -46 4

>Hsp70_D_sechellia_D

TGTATGTACATAGCATTTAGTTTCCAAAGCTGCCAATCACCTACATACTATGTACGTACATAATTAATTTCAATGAATCTAGTACACATTTAGATGCTTACATATGTAAATTAAGTTCCAACTAATTTCTTATACAGTTTCCTAACCCTTAAAGGTCTATTATCGTATATTAAAACAGAAGAAAGTCCATTAATAGTTTAATTCATTAACTAAAATAATCTTTAATGTATGCCGGAGCAGACATTTATTGGTTTAAAAACGGTGGCATTCGCATAACAAAGCGCTTCGATTATCTTTAACATAAGTTATTTAAGCAGCCGTATTTATAAAGAAATTTCCAAAATAAAGCGAAATTTCTAGAATCCCAAAACAAACTGGTTGTTGCGGTAGGTCATTTGTTTGGCAGAAAGAAAACTCGAGAAATTTCTCTGGCCGTTATTATCTCATCGTTTTGTGGCTCTCCCTCTCTGCACTAAAGCTCCCTCACTCTGTCACACAGTAAACGAAAGACTGTTCTCGTTGCTTCGAGAGAGCGCGCCTCGAATGTTCGCGAAAAGAGCGCCGGAGTATAAATAGAGCCGCTTCGTTCCCAGAGCGTCA

ATTCAATTCAAACAAGCAAAGTGAAAACATCGCTAAGCGAAAGCTAAGCAAACAAACAAG

-252 -238 3

-192 -173 4

-88 -69 4

-65 -46 4

>Hsp70_D_yakuba_A

ACATATGTACGTATATGTATGTACATATGCCCAGGTCATCAACCTGGGTAAGGACATATGTATATTCGGTTCTACCTCTTGCAGTTCACAAAATAATGCAAAACCCCATTTGCTTAAATATCTTTAACTTATTAAACATCTTATATAAGCATTGCGGGACGTCTAGCAGGACAAGGCTACATATATTCATAGAGTTAAAGGAAATACATTGGAATATCTTTGGTTTTGCGGTATAAAGCAACTTCGAATTCAGTGTATTGGGCACCGAAAATCAGCATATCAAAGCGCTTATCTTTAAAAAAAGTTTGTTACGCTGCCGTTTATATAAAGCTTTTCCAAAATACCACAAACATTCTAGAATTTAAAAAACAACATAATTGTTGTGGTAGGTCTTTTGTTTGACAGAAAGAAAACTCGAGAAATTTCTTTTCCATTATTTCTCTCTTAGTTTTGCGGCTCTCCCTCCCTGAGCCTAATGCTATCTTATTCTGGCACATACTAAACAGCGTACTATTCTCGTTGCTTCGAGAGAGCACGCCTCGAATGTTCGCGAAAAGAGCGCCGGAGTATAAATAGGGCCGCTTGGTTCTCTTAGCGTCA

ATTCAATTCAAACAAGCAAACTGAAAACATCGCTAAGCGAAAGCTAAGCAAACAAACAAG

-249 -240 2

-193 -174 4

-88 -69 4

-65 -46 4

>Hsp70_D_yakuba_B

GCTTAATCTCAACTTTCTAGCTTTTGTAGTTCCTGAGATCTCGACGTTCATACGGACAGACGGACAGACGGACAGACAGACGGACGGACAGACGGACATGGCCATATCGACTCTGATATTGATCCTGATCAAGAATATATTCTTTATATGGAGGCACCCCGCACCCATAACCCCACACCCCAGTTAAATATATTTAACTTTTTGAACATCTTATATTAATATTGCGGGACGTCTAGCAGGACAAGGCTATATTCATAGAGTTAATAGAATTATCAAAGCGCTGCGATTATCTTTAAGAAAAAGTTAGTTACGCTGCCGTTTATATAGGGCCTTTTCAAAATACCGCAAACATTCTAGAATGCGAAAAACAATCTAGTTGTTGTGGTAGGTCTTTTGTTTGGCAGAAAGAAAACTCGAGAAATTTCTTTTCCGTTATTTCTCTCTTAATTGTGCGGCTCTCAATCCCTGGAAACAAATGCCTTCTCATTCTGGCACATACTAAACAGCGTGCTGTTCTCGTTGCTTCGAGAGAGCACGCCTCGAATGTTCGCGAAAAGAGCGCCGGAGTATAAATAGGGCCGCTTGGTTCTCTTAGCGTCA

ATTCAATTCAAACAAGCAAACTGAAAACATCGCTAAGCGAAAGCTAAGCAAACAAACAAG

-250 -241 2

-194 -175 4

-88 -69 4

-65 -46 4

>Hsp70_D_yakuba_C

ATACATAAACTTTATATGGTCGGAAACGCTTTATTTTGCCTGTTACATACTTTTAAACGAATCTAGTATTCTCCTTTACTCTAAGAGTAAGGGGTATACTTGTATCTATATCTGTTTAGAATTTCAATTATATTACACATAAACCTATCCACATATATAATATAAGTTTAAATAAGAAGACTGAACATATCTGCCGGTTAATGTTGGTTATAACTAATAGAAATTTTGTCAGAGATATGTACAAGTAGTCTCACTAGTGGAAACCATCCGTATATCAAAGCGCTCCGATTATCTTTAAAAAAAGTTTGTTACGCTGCCGTTTATATAAAGCTTTTCCAAAATACCACAAACATTCTAGAATTTAAAAAACAACATAATTGTTGTGGTAGGTCTTTTGTTTGACAGAAAGAAAACTCGAGAAATTTCTTTTCCATTATTTCTCTCTTAGTTTTGCGGCTCTCCCTCCCTGAGCCTAATGCTATCTCATTCTGGCACATACTAAACAGCGTACTATTCTCGTTGCTTCGAGAGAGCACGCCTCGAATGTTCGCGAAAAGAGCGCCGGAGTATAAATAGGGCCGCTTGGTTCTCTTAGCGTCA

ATTCAATTCAAACAAGCAAACTGAAAACATCGCTAAGCGAAAGCTAAGCAAACAAACAAG

-249 -240 2

-193 -174 4

-88 -69 4

-65 -46 4

>Hsp70_D_yakuba_D

GCCCAGGTCATCCACCTGGGTAAGAACATATGTATATTCGGTTCTACCTCTTGCAGATCACAAAATAATGCAAAACCCCATTTGCTTAAATATCTATAACTTGTTAAACATTTTATATAAACATTGCGGGATGTCTAACAGGACATGGCTATATTGATAGAGTTAAAGGAAAGACATTGGAATCATTATTATTCCATTCTAATATCTTTGGTTTTGCGGTATATAGCAACTTCGAATTTTGTGTATTGGGACTGGGTGGGGTCCATCCGCATATTAAAGCGCTGCGATTTTCTTTAAAAAAAAGTTAGTTACGCCGCCGTTTATATAAAGCTTTTCCAAAATACCGCAAACATTCTAGAATGCGAAAAACAATCTAGTTGTTATGGTAGGTCTTTTGTTTGGCAGAAAGAAAACTCGAGAAATTTCTTTTCCGTTATTTCTCTCTTAATTTTGCGGCTCTCACTCCCTGAAACCAATGCCATCTCATTCTGGCACATACTAAACAGCGTGCTGTTCTCGTTGCTTCGAGAGAGCACGCCTCGAATGTTCGCGAAAAGAGCGCCGGAGTATAAATAGGGCCGCTTGGTTCTCTTAGCGTCA

ATTCAATTCAAACAAGCAAACTGAAAACATCGCTAAGCGAAAGCGAAGCAAACAAACAAG

-249 -240 2

-193 -174 4

-88 -69 4

-65 -46 4

>Hsp70_D_santomea_A

NGGACGGACAGACAAACGAACGGACAGACGGACATGGCCAGATCGACTCGGCTATTGATCCTGATCAAGAATACAAATACTTTATATGGTCGGAAACGCTTTATTTTGCCTGTTACATACTTTTAAACGAATCTAGTATTCCCCTTTACTCTAAGAGTAAAGGGTATACTTGTACATATATGTTTACATATATACGTACATATATATGTACATATATGTATGTATGTTTAGAATTTCAATTATGAATATGTGCATACATATTGTAACACATAAACCTATCCATATATATAATATAGGTTTAAATAAGAAGACTGAACATATCTGCCGGTTAATGTTGGTTATAACTAATAGAAGTTTTTTCAGAGATACATGTAGTCTCACTAGTGGAAACCATCCGTATATCAAAGCGCTCCGATTATCTTTAAAAAAAAGTTTGTTACGCTGCCGTTTATATAAAGCTTTTCTAAAATACCGCAAACATTCTAGAATTCAAAAAACAACAGCAGCGTACTGCTCTCGTAGCTTCCAGAGAGCACGCCTCGAATGTTCGCGAAAAGAGCGCCGGAGTATAAATAGGGCCGCTTGGTTCTCTTAGCGTCA

-121 -112 2

-88 -69 4

-65 -46 4

>Hsp70_D_santomea_B

NNNNNNNNNNNNNNNNNNNGGATATGAACCTATGTATATATATAGATATGCATGTATTAATGTATGCACATATGTACATACATACATATGTACGTATATGTACATATGCCCAGGTCATCAACCTGGGTAAGGGTAAGGACATGTATATTCGGTTCTACCTCTTGCAGATCACAAAATAATGCAAAACCCCATTTGCTTAAATATCTTTAACTTATTAAACATCTTATATAAGCATTGCGGGACGTCTAGCAGGGCAAGTCTACATATATTCATAGAGTTAAAGGAAATACATTGGAATATCTTTGGTTTTGCGGAATAAAGCTACTTCGAATTTTGTGTATTGGACACCGAAAATCAGCATATCAAAGCGCTCTGATTATCGTTAAAAAAAGTTTGTTACTCTGCCGTTTATATAAAGCTCTTCCAAAATACCACAAACATTCTAGAATTAAAAAAACAACATAATTGTTGTGGTAGGTCTTTTGTTTGACAGAAAGAAAACTCGAGAAATTTCTTTTCCATTATTTCTCTCTTAGTTTGCGGCTCTCCCTCCCTGAAACTAATGCCATCTCATTCTGGTACATACATAGAGCAGCGTACTGCTCTCGTAGCTTCCAGAGAGCNCGCCTCGAATGTTCGCGAAAAGAGCGCCGGAGTATAAATAGGGCCGCTTGNNNNNNNNNNNNNNN

-250 -241 2

-194 -175 4

-88 -69 4

-64 -45 4

>Hsp70_D_erecta_A

AATTCGTTTCATAGATCAAAATTGATTTCGGACCGAAAATCGACTTCTAGCCCAAGTACGCACACATACACACATTCTTGCTTACTGTTTTCATTCGCACAAGCAAGTAAATTATATTTTTCGATTTTTATGCTCTCAATTTTAAGCGAGCAGGGAGAAAGCAGTAATTGTTATTCTAATGTCTTTTCTGGAGCTAAATTAAAACATTATTTAATTTATTTATTTAATGTATCGTGCCAATACCTTCCTAATTAAAAATTTTGTCAGAGCTCACTGGTGGGCATCATCTGCATAACAACGAGCAGCGATTATTATAAAAATTAGTTTTTCACGCTGCCGTTATTATACAGAAATTTTCAAAATAGTGCGAATATTCCAGAATCCGCAAGAGAATCTAGTTGTTGTGGTAGGTCATGGGTTGGAAATTTCTCCTCTGTATTCTCTTAATTTTGCGCCTCACCTTCCCAGACATTATTGCTATCTCATTCTGGCACATACTAAACAGCATACTGTTCTCGTTGCTTCGAGAGAGCACGCTTCGAATGTTCTCGAAAGGAGCGCCGGGTGTATAAATAGCGCTGCTTGTTTCTTTTAGCGTCA

TTTCAATTCAAAGAAGCAAAGTGAACACATCGCTGAGCGAAAGCTAAGCAACCAAATAAG

-233 -219 3

-180 -171 2

-89 -70 4

-61 -47 3

>Hsp70_D_erecta_B

AATTCGTTTCATAGATCAAAATTGATTTCGGACCGAAAATCGACTTCTAGCCCAAGTACGCACACATACACACATTCTTGCTTACTGTTTTCATTCGCACAAGCAAGTAAATTATATTTTTCGATTTTTATGCTCTCAATTTTAAGCGAGCAGGGAGAAAGCAGTAATTGTTATTCTAATGTCTTTTCTGGAGCTAAATTAAAACATTATTTAATTTATTTATTTAATGTATCGTGCCAATACCTTCCTAATTAAAAATTTTGTCAGAGCTCACTGGTGGGCATCATCTGCATAACAACGAGCAGCGATTATTATAAAAATTAGTTTTTCACGCTGCCGTTATTATACAGAAATTTTCAAAATAGTGCGAATATTCCAGAATCCGCAAGAGAATCTAGTTGTTGTGGTAGGTCATGGGTTGGAAATTTCTCCTCTGTATTCTCTTAATTTTGCGCCTCACCTTCCCAGACATTATTGCTATCTCATTCTGGCACATACTAAACAGCATACTGTTCTCGTTGCTTCGAGAGAGCACGCTTCGAATGTTCTCGAAAGGAGCGCCGGGTGTATAAATAGCGCTGCTTGTTTCTTTTAGCGTCA

TTTCAATTCAAAGAAGCAAAGTGAACACATCGCTGAGCGAAAGCTAAGCAACCAAATAAG

-233 -219 3

-180 -171 2

-89 -70 4

-61 -47 3

>Hsp70_D_erecta_C

AAAAAAGAATTTCTAAATTTAAATGCAGATTTATAGAGAATTTCGCCGGAAACATTTATAGATATGCCACTTCAAAATCCGATTAAGATAAGTTGAGTCTTACGCGAGCGAGGAGAAAGCAGTTATTGTTATTCTAATGCCTTTTCTGGAGCTCAGTATCTGAGAGTCTGAGTCCAGCAATGCGTTTTATTGAAATTTAAAACATTATTTAATTTAATTATTTAATGTATCGTGCCAATACTTTCCTAATTAAAAATTTTGTCAGAGCTCACTGGTGTGGGCATCATCCGCATAACAACGAGCAGCGATTATTATTAAAATTAGTTTTTCACGCTGCCGTTATTATACAGAAATTTTCAAAATAGTGCGAATATTCCAGAATCCGCAAGAGAATCTAGTTGTTGTGGTAGGTCATGGGTTGGAAATTTCTCCTCTGTATTCTCTTAATTTTGCGCCTCACCTTCCCAGACATTATTGCTATCTCATTCTGGCACATACTAAACAGCATACTGTTCTCGTTGCTTCGAGAGAGCACGCTTCGAATGTTCTCGAAAGGAGCGCCGGGTGTATAAATAGCGCTGCTTGTTTCTTTTAGCGTCA

TTTCAATTCAAAGAAGCAAAGTGAACACATCGCTCAGCGAAAGTTAAGCAACCAAATAAG

-233 -219 3

-180 -171 2

-89 -70 4

-61 -47 3

> Hsp70_D_erecta_D

TCAAATTCGTTTCATAGATCAAAATTGATTTCCGACCGAAAGACTTCTAGCCCAAGTACGCACACATACACACATTCTTGCTTACTGTTTTCATTCGCACAAGCAAGTAAATTATATTTTTCGATTTTTATGCTCTCAATTTTAAGCGAGCAGGGAGAAAGCAGTAATTGTTATTCTAATGTCTTTTCTGGAGCTAAATTAAAACATTATTTAATTTATTTATTTAATGTATCGTGCCAATACCTTCCTAATTAAAAATTTTGTCAGAGCTCACTGGTGGGCATCATCTGCATAACAACGAGCAGCGATTATTATAAAAATTAGTTTTTCACGCTGCCGTTATTATACAGAAATTTTCAAAATAGTGCGAATATTACAGAATCCGCAAGAGAATCTAGTTGTTGTGGTAGGTCATGGGTTGGAAATTTCTCCTCTGTATTCTCTTAATTTTGCGCCTCACCTTCCCAGACATTATTGCTATCTCATTCTGGCACATACTAAACAGCATACTGTTCTCGTTGCTTCGAGAGAGCACGCTTCGAATGTTCTCGAAAGGAGCGCCGGGTGTATAAATAGCGCTGCTTGTTTCTTTTAGCGTCA

TTTCAATTCAAAGAAGCAAAGTGAACACATCGCTGAGCGAAAGCTAAGCAACCAAATAAG

-233 -219 3

-180 -171 2

-89 -70 4

-61 -47 3

>Hsp70_D_erecta_E

ATGTCCCGAGGCTGAAGTTCTAATCTCCATAACTCAACTTATCTTAATCGGATTTGAGTATGTGAGAGTTATTATATTTAGTCCAACAATGCGTTTTATTTAAATTTAAAACAAAATTAACATTTTTAATAGGCCTAAATGTACATACATATCTTGTCAAAACCTTCCCAATAAGAAATTTTGTTAGAGCTACATACATACATACAAAACAAAATTAACATTTTTAATAGGCCTAAATGTACATACATATCTTGTCAAAACCTTCCTCACTGGTGGACACTATCCGCATTACAAAGCGCTGCGATTATCTTTAAAATTAGGTTTTCATGCTGCCGTTTATATAAAGAAATTTCCAAAATAAAGCGAATATTCTTGAATGCGAAAAACAATCTAATTGTTATGGTAGGTCATTTGTTTGGCAGAAAGAAAACTCGAGAAATTTCTTCTCCGTTATATCTCTCCGTCCCTCCCAGACACTATTGCTCTCATTCTCACATACTAAACAGCATACTGTTCTCGCAGCTTCGAGAGAGAACGTCTCGAACGTTCGCGAAGAGAGCGCCGGAGTATAAATATGGCCGCTTGGTGCTCCCAGCGTCA

ATTCAATTAAATCAAGCAAACTGAAAACATCGCTGAGCGGAAGCTAAGCAACCAAATAAG

-237 -223 3

-176 -157 4

-88 -69 4

-65 -46 4

>Hsp70_D_erecta_F

TTAGAGGTATACCACTTCAAAATCCGATTAAGATAAGTTGAGTTTTACGTGGGCGGGGAGAAAGGGTTATTGTTATTCTAATGTCTTTTCTGGAGCTCTGTATGTGGGAGTTTGAGTTCAGCAATGCGTTTTATTGAAATTTAAAACTTAATTTAATTTATTTATTTATTTATAAATTTTGTCAGAGCTCACTGGTGGGCATCATCCGCATAACAACGAGCAGCGAATATTATTAAAATTAGTTTTTCATGCTGCCGTTATTATACAGAAATTTCCAAAATAGTGCGAATATTCCAGAATCCGAAAGAGAATCTAGTTGTTGTGGTAGGTCATAAGGTAGGAAATTTCTCCTCGTTATTCGCTCTTAAATTTGCGCCTCTCTCTCCCATACACTATTGCTATTTCACTCTGGCACATACTAAACAGCATACTGTTCTCGTTGCTTCGAGAGCACGCCTCCCCTCCCAGACACTATTGCTATTTCATTCTGGCACATACTAAACAGCATACTGTTCTCGTTGCTTCGAGAGAGTACGATTCGAATGTTCTCGAAAGGAGCGCCGGGTGTATAAATACCGCTGCTTGTTTCTTTTAGCCTCA

CTTCAATTCAAAGAAGCAAAGTGAACACATCGCTGAGCGAACGCTAAGCAACCAAATAAG

-315 -301 3

-168 -149 4

-89 -65 5

-61 -47 3

>Hsp70_D_ananassae_A

TTGGCCGATTTCCATCCGATTCTGGAGCGGAATACCTTTAACGATTTGTAGATAGATTCTCCATCATTCTGCATTAAAATCTTCGAACAAAATATTTTTCAGATTTTTTCTCAAATTTCCTTGGGGTCCCCTTTAAAATTGACGATGAAAGCAGTGAGGGGCTATTGTGGAGGCTATATCTTCGCCAATAATCATCAGATCTTAAACTATTCTTTTTAATTCGCATTAAAATGTGAAAAATGTGTTGTAAATTGGTGGAATAAATTGTTGGTGGGTCTCCTTTTTTGGTCAAGGTAAGGTTTTGGGGCCTGTCTGCAAAAATATACAAAATTCGATTTTCAGATTTCTAGAAATATCTCTTTTTGGTGTAGGTTTAAATGTTTGGCGGGGAAAAATCGAGAAAATTCTATAGCTGCATTGACGGGAAGCATTAACCCACCCTCTCTTAAAATACTGTTATACTGTTTGTGTGGCCATTACCACACAAACCTCTCGAAGCGCCGAGAGAGCAGTTCTCGAACCTTCGAGGTGAGAGCAAGAAAGTTCGCGAAGAGATAGCCGGGGTATAAATAGCCGGCCGTTTCTCTTCGCTTCAATTCA

ATTCAATTCAAACAAGCGAACTAAACACATCGCGAAAGCGAAGCAACCAAACCAAGCAAA

-257 -243 3

-212 -193 4

-89 -70 4

-68 -49 4

>Hsp70_D_ananassae_B

TTGGCCGATTTCCATCCGATTCTGGAGCGGAATACCTTTAACGATTTGTAGATAGATTCTCCATCATTCTGCATTAAAATCTTCGAACAAAATATTTTTCAGATTTTTTCTCAAATTTCCTTGGGGTCCCCTTTAAAATTGACGATGAAAGCAGTGAGGGGCTATTGTGGAGGCTATATCTTCGCCAATAATCATCAGATCTTAAACTATTCTTTTTAATTCGCATTAAAATGTGAAAAATGTGTTGTAAATTGGTGGAATAAATTGTTGGTGGGTCTCCTTTTTTGGTCAAGGTAAGGTTTTGGGGCCTGTCTGCAAAAATATACAAAATTCGATTTTCAGATTTCTAGAAATATCTCTTTTTGGTGTAGGTTTAAATGTTTGGCGGGGAAAAATCGAGAAAATTCTATAGCTGCATTGACGGGAAGCATTAACCCACCCTCTCTTAAAATACTGTTATACTGTTTGTGTGGCCATTACCACACAAACCTCTCGAAGCGCCGAGAGAGCAGTTCTCGAACCTTCGAGGTGAGAGCAAGAAAGTTCGCGAAGAGATAGCCGGGGTATAAATAGCCGGCCGTTTCTCTTCGCTTCAATTCA

ATTCAATTCAAACAAGCGAACTAAACACATCGCGAAAGCGAAGCAACCAAACCAAGCAAA

-257 -243 3

-212 -193 4

-89 -70 4

-68 -49 4

>Hsp70_D_ananassae_C

TTGGCCGATTTCCATCCGATTCTGGAGCGGAATACCTTTAACGATTTGTAGATAGATTCTCCATCATTCTGCATTAAAATCTTCGAACAAAATATTTTTCAGATTTTTTCTCAAATTTCCTTGGGGTCCCCTTTAAAATTGACGATGAAAGCAGTGAGGGGCTATTGTGGAGGCTATATCTTCGCCAATAATCATCAGATCTTAAACTATTCTTTTTAATTCGCATTAAAATGTGAAAAATGTGTTGTAAATTGGTGGAATAAATTGTTGGTGGGTCTCCTTTTTTGGTCAAGGTAAGGTTTTGGGGCCTGTCTGCAAAAATATACAAAATTCGATTTTCAGATTTCTAGAAATATCTCTTTTTGGTGTAGGTTTAAATGTTTGGCGGGGAAAAATCGAGAAAATTCTATAGCTGCATTGACGGGAAGCATTAACCCACCCTCTCTTAAAATACTGTTATACTGTTTGTGTGGCCATTACCACACAAACCTCTCGAAGCGCCGAGAGAGCAGTTCTCGAACCTTCGAGGTGAGAGCAAGAAAGTTCGCGAAGAGATAGCCGGGGTATAAATAGCCGGCCGTTTCTCTTCGCTTCAATTCA

ATTCAATTCAAACAAGCGAACTAAACACATCGCGAAAGCGAAGCAACCAAACCAAGCAAA

-257 -243 3

-212 -193 4

-89 -70 4

-68 -49 4

>Hsp70_D_ananassae_D

TGTTTTAAAAAAAGGGATTGGGGTTGGGGATCTAAATATTTAATATCACTATAACCCACCATCTATAACTTCCAAAAACTGACCTCAAAAAATCGTAATAAGTCTGGTTATATTTAGATTAATAGCTTCGGATGTTTATTTTATAAAAAAAACCTTGATAAACGTTATCTCGAAGATTTCCCGAAAATATTTCTCGCAGCTCGGAATATTCTATGGACAACGGCACCTTTCTGCCTTCCTGGTAGTAGTTCATGTCGCAGGTGGGTCTCCATAAGTGCTTTTTGGGGGGCCATCTTCTATGTCGTTGCTGATACGACAGCTGTGTTTATACCAGTCCGCAAAAACAAACAAAATATGATATTCAGATTTCTAGAAATATGTTTTGTTTGAATGTTTGGCGGGGAAAAATCGAGAAACTTCTATTGCTGCATTAACCCGCCCTCTCTTAAAATACTGTTATACTGTTTCTGTGGCCATTGCCACACAAACCTCTCGAAGCGCCGAGAGCGCAGTTCTCGAACCTTCGAGGTGAGAGCAAGAAAGTTCGCGAAGAGATAGCCGGGGTATAAATAGCCGGCCGTTTCTCTTCGCTTCAATTCA

ATTCAATTCAAACAAGCGAACTAAACACATCGCGAAAGCGAAGCAACCAAACCAAGCAAA

-234 -225 2

-199 -180 4

-89 -70 4

-68 -49 4

>Hsp70_D_ananassae_E

ATTACGATTTTTTGAGGTCAGTTTTTGGAAGTTATAGATGGTGGGTTATAGTGATATTAAATATTTAGATCCCCAACCCCAATCCCTTTTTTTAAAACATTTACTTTGTTTTAAATTTTAGGAACCAAACATCATTTAACTGGAATGACCCTATTTTTAATTTCATGTTACATATTTCATACATTTTTTATGAATTTGATGTCTTGCTTTAAGGCTTAATATTAAGAAGTGTCTGACCTCAGCTGGATGAATTCTTAAAGTCCTCCTCTGCTACGATAGCTCAAATAGCCAGTTGTATTTATACCTTGCTGCGAAAACTTTCAAAGCTAAATTGCTTATACTTAGGATTTCCAGAAATATCTTTTGTTGTCGTAGGTTTGAATGTTTGGCGAAGAGAAGTCGAGAAAATTCTATAGCTGCATTGACGGGAAGCATTAACCCACCCTCTCCTAAAATGCTGTTATACCTCATGGTTATAGCCATACGGGCCTCTCGAAGCGCCGAGAGCGCAGTTCTCGAACCTTCGAGGTGAGAGCAAGAAAGTTCGCGAAGAGATAGCCGGGGTATAAATAGCCGGCCGTTTCTCTTCGCTTCAATTCA

ATTCAATTCAAACAAGCGAACTAAACACATCGCGAAAGCGAAGCAACCAAACCAAGCAAA

-253 -239 3

-208 -189 4

-89 -70 4

-68 -49 4

>Hsp70_D_pseudoobscura_A

AATGGGAATTTTAGATTTTTCATATTAAGGCAAAGGCTATGTACCTTATATTTATGATAACTTTAATTCCTTTATTTATTAATTCCCTCAGAAATAGAATCGTAACGGGACCAGATTCATCCATTTCACTATCACTCGAGATGAGGATTTTCCTGTTGAAAAAAAAAACCATTTGACTTAGATCACAAATTTTTTATTTTTATACTAAAGAATATAATTAAGATTTGGGTATCTCGCGTTAATTGCTCATCAAACATTAAGGTAAAAGTATGCATTGTATGTATTTGGATTGACATGCTCCAGAAATCAACCGAATTTTCTAGATTCCCAAAAACAAGCCGCTTGTTGCGGTAGGTCAGATACTCGAACCTCGCAGATACATTGGCCACAGGAAAAGTCGAGAAATTTCGTCGACCCTCTCTCACGAGTACAAGCTGTTTGCGCTCTCTCTGCGGCCTCTGACAGCTGCCAAGACTCGTTGTCTCGCTCTGACGCACGCGATTCAGGGTAACATTCAAGATATTTCTAGAAGAGAGCTCTCGAAGTTTCGAACGGAGAGCGGCCAGGTATAAATACAGCCGCCAGTTTCTCTTCGCAGCA

ATTCAATTCAAACAAGCGAAGAGAACACATCTCAAAAGTGAAACAAAGCGAGCTACCAAA

-289 -275 3

-210 -191 4

-88 -69 4

-65 -51 3

>Hsp70_D_pseudoobscura_B

TCAATTTTAAAAGTGTAAAAGTGTATGTTTAAAAATTGATTCAAAAGTTTTGAGGTGAGGCACTACTATTTCCATGAACACAGCTGATGTACAGACTTTATGTTCTTGAATAGCCAGATCGTCTGTCGAAACCGGACCACTTATCACAAATGGGTCCTATTTTATAGTAGTTGTATTAACGGAATATATATATATTAACATATATACATACATACATATCTACCAATACCTTGCGCGTATTGCTCGTCTAACATCAAGATAAATGTACATATGTATGTTTTATATGGACATAAAAACTTCCGGAAATCATCCGAATTTTCTAGATTCCCAAAAACAAGCCGCTTGTTGCGGTAGGTCAGATACTCGAACATCGCAGATACATTGGCCACAGGAAAAGTCGAGAAATTTCGTCGACCCTCTCTCACGAGTACAAGCTGTTTGCGCTCTCTCTGCGGCCTCTGACAGCTGCCAAGACTCGTTGTCTCGCTCTGACGCACGCGATTCAGGGTAACATTCAAGATATTTCTAGAAGAGAGCTCTCGAAGTTTCGAACGGAGAGCGGCCAGGTATAAATACAGCCGCCAGTTTCTCTTCGCAGCA

ATTCAATTCAAACAAGCGAAGAGAACACATCTCAAAAGTGAAACAAAGCGAGCTACCAAA

-289 -275 3

-210 -191 4

-88 -69 4

-65 -51 3

>Hsp70_D_persimilis_A

AATTTTAAAAGTGTAAAAGTGTATGCTTCAAAATTGATTCAAAAGTTTTGAGGTGAGGCACTACTATTTCCATGAACACAGCTGATGTACAGACTTTATGTTCTTGAATAGCCAGATCGTTTGTCGAAACCGGACCACTTATCACAAATGGATCCTTATATATAGTAGTTGTATTAACGGAATACTTATATACATACATACATATATATCTATCAATACCTTGCGCGTATTGCTCGTCTAACATCAAGATAAATGTACATACATATGTACATATGTTTGTACATATGGACATAAAAACTTCCGGAAATCATCCGAATTTTCTAGATTCCCAAAACAAGCCGCTTGTTGCGGTAGGTCAGATACTCGAACCTCACAGATACTTTGGCCAGAGGAAAAGTCGAGAAATTTCGTCGACCCTCTCTCACGAGTACAAGCTGTTTGCGCTCTCTCTGCGGCCTCTGACAGCTGCCAAGACTCGTCGTCTCGCTCTGACGCACGCGATTCAGGCTAACATTCAAGATATTTCTAGAAGAGAGCTCTCGAAGTTTCGAACGGAGAGCGGCCAGGTATAAATACAGCCGCCAGTTTCTCTTCGCAGCA

ATTCAATTCAAACAAGCGAAGAGAACACATCTCAAAAGTGAAACAAAGCGAGCTACCAGA

-289 -275 3

-210 -191 4

-88 -69 4

-65 -51 3

>Hsp70_D_persimilis_B

AAAGGAATAATATATTATTGAGAGACGAAACAAAAATAAATCTTTTTAATAATGACTCCAGAAGATGTATACAACGACCCAAAGGCAAAGCATTCGACTTCCGGTACAACGGGTCCAGATTCATCCATTTTACTATCACTCGAGATGGGGATTTTCCTGTTGAAAAAAAAACCTTTTGACCTAGATCACAAATTTTTTTTTTATACTAAAGAATATAATCCAGATTTGGGTATCTCGCGTTAATTGCTCATCAAACATTGAGCTAAAAGTATGTATTGTATGTATTTGTATTGATATGCTCCAGAAATCAATCGAATTTTCTAGATTCCCAAAACAAGTCGCTTGTTGCGGTAGGTCAGATACTCGAACCTCGCAGATACTTTGGCCACAGGAAAAGTCGAGAAATTTCGTCGACCCTTTTTCACGAGTACAAGCTGTTTGCGCTCTCTCTGCGGCCTCTGACAGCTGCCAAGACTCGTTGTCTCGCTCTGACGCACGCGATTCAGGGTAACATTCAAGATATTTCTAGAAGAGAGCTCTCGAAGTTTCGAACGGAGAGCGGCCAGGTATAAATACAGCCGCCAGTTTCTCTTCGCAGCA

ATTCAATTCAAACAAGCGAAGAGAACACATCTCAAAAGTGAAACAAAGCGAGCTACCAGA

-289 -275 3

-210 -191 4

-88 -69 4

-65 -51 3

>Hsp70_D_willistoni_A

AACAATGATTTTTCGTTTAAGCTGAAAAAGAAAATTAGTTATTTTTAATTTAAGTTTGATAATATTGCAACCTATGTAAACATACTCAAATATTCGCCTCAACAAAAGTCTAGACTTGAACTGTCTGGCGCCAGCGTTCTGTATGTATGTACTTATGTATGTTTGTATTGCAACTCATTCATCAAAATATCAGATCCCACTATCCACAGTCGGTCAACGACTCAAAGCTTGTTAACGCTATCTACAGGTTTAATTCACGTGAGTTCAGATTAGCAGAAAAGATTGTTTAGTTTAAAGCCACTAAAGACCAATAATAGTCTTTATTAAAAAAAAAAAAAAAAAACCAAGGCACTGAATCTTCTAGAGCCGAGTTCTAATGTTACCTTGCAATGATTGTTGAGGTAGGTAGCGAAAAAAAGAGAAAACGAGAACTTAACGAAATTTCCACCCTCTGCAGTATTGTGCGCCAAATATGTCTTCTCCCGCTTACACTCACAGCTATCTGTCGATTGTTCGAGAGAGTTCGCGAAGAGAACGTCTCGAAGTTTCCGAAAGAGAGCGGCCAGGTATAAATAGGGCCGGCAATTTCTCTTCGCAGCA

ATTCAATTCAAGCAAGCGAAGTGATAACATCACAGAAACAAGCAAAAGCAAAACAAGCGA

-248 -234 3

-164 -155 2

-94 -70 5

-65 -46 4

>Hsp70_D_willistoni_B

CATTTTTGTTCTCTTTTTTGCTCTTCCAAATCAGAGAGACTGTCAGTAAGGCAAGAGAAAAGAAACTTCAAACCAAAATTCTTTTAGTCAAGTTTTACTGTAAAATAAAGTGAGCTGTTTGTTTTGGTGCTATAATTTTCACCGGCACTGTACGTACATATGCACAAATTTAGTTTGGACATCTTCGATCTAAAACTCCATTATTTAAGGATTACTCCTGTGGGAGCTTTTTATATAGTGGTCGATCCAGACTGACTGATACATCGATAGTGACAGACCAAAAATACATAAATAAATAAATGTATGTATAAAGTAATAATAAAGAAATAATTTGTTATTCGTTTCACACTTCACAATGAATATACTAGTTCGGTAAGGGTATACAAAAGAGAACAGATACTTCAAGAAAATACCGTTCCTAACAGAGGCTATCGCACCGCAGGGGCAGATATACATACATATGTGAATATGCATATTCTCTCCCGCTCACACTCACAGCTATCTGTCGATTGTTCGAGAGAGTTCGCGAAGAGAACGTCTCGAAGTTTCCGAAAGAGAGCGGCCAGGTATAAATAGGGCCGGCAATTTCTCTTCGCAGCA

ATTCAATTCAAGCAAGCGAAGTGATAACATCACAGAAACAAGCAAAAGCAAAACAAAGCG

-244 -230 3

-206 -187 4

-94 -70 5

-65 -46 4

>Hsp70_D_willistoni_C

AAAAAACAAAGACAACTTTAAACACTAACGGTGGATATGCAAAAACAAGATTGTTTAAGTACGGTTACTAATAATTCCGGTTTCTGATCGTAGCCCACTATTCATCAAAACATGAGATCCCACAGATCCATGGTCAATAAAAGATCGTTGACAGGCCTCCACAGCAGCAGCTTTGATACACGTGAGCTGAGATCGAGAGATTAAAACGTCACGACTACAGGCATTCGAGTCAAATAATATTCTTATTTTAATATCTACATATGTATGTACATATATTTTTGTACATACATACATATGTCCAATTCATATAAAAATAGGTAAAATGAGCAATCCAGTATTGAGTTTTCCAAAATAAACAACATGCATATCTAATTTTAAATTACAAACATTCTAAGACCTACCTCAAAAAACAAAACAAAAAGAGAGAGCCACAGATACTTCGAGAAAATTCTGCTCTTAGCGCAGGGGTAAACGTATGCTATCTCGCTTACACTCATTGAGTACTGCCGATTGTTCGAGAGAGTGCGAGAAGAGAGCGTCTCGAAATTTCGAAAAGAGAGCGTCCGGGTATAAATAGGGCCGACAATTTCTCTTCCAGCA

ATTCAATTCAAACAAGCGAAGTGATAACATCACAAAAACAAACAAAACATAAGAAAAGCG

-262 -248 3

-168 -149 4

-93 -69 5

-64 -45 4

>Hsp70_D_mojavensis_A

GTATGGAGATCCCTGTATGTAAGTACATATTACATTTCTATTATCAACAACACATTGATAGGATGCACACAAAAAAGTTTTAATACTTTTGGCTAGAAAATATATGTATGTACCTATGTACATGCTTAAATACATATGTATGTATATTGGTAAAAGAAAATTCAACGATCATAAGGAAAATCGGTTATTGTACACAGACCTTGACTGTGCATATGATATATGTATGTAAGTCTAACAACTAGTAATAGAACCAGAATATAAATAACTAGCATGTTTATAAATATCAAATTGTTAAAAATAATCAATTTATTTATATCTTGTTCTCCACTGCCGAGGAATATGTATGTATGTATGTTAATTATACGGTGAAACTATACACAAAATATTTTCACTCAGTCCGTCTCTTGCAGCATATCAATATGAGAGAATATTCGAGAATATTCCAACCACTCCCACAGTAAACAACAGCCGCTCTCACTCACTCACACTCGCAAAAACAGTCGAGTCGATTCAGTCGAGTCGATTCGAGAGAGCGCGTCGAGAAATTTCGAACAGAGAGCGCCAGAGTATAAATACGGCAGAAGTTCGCTCTTTGGCTCA

ATTCAATTCAAGCAAGTGAACTGAAAACGTCGAAAGACAAAAGCGGAATACAAACAAGCG

-266 -252 3

-176 -157 4

-88 -69 4

-65 -51 3

>Hsp70_D_mojavensis_B

TCCGCCAATAAAAAACTTCAAATATTAATATTAAAGCATATTTCATGTATACATACATACATATGTATGTATACATACATACATATGTATGTATATCCATACATACATATATATGTGTGCACATACATATGTATGTACATATGTATGTATGTAAGAGTATATGTACATACATATATACACATGAACATACATACAAACATACATATGTCTGTGTTTGACTGCCAAACACGAGTTCCACTGTATATGCTTCGATTTTGAAACGTGATGTCGAAAACAAGTCTGTTAAGTCAGCATTCGCTTATTCAAGTATGAGAATACTTAGAAGCACCAATGAATTTTCTAGAAATTGAAATGATAGCCAATTGTTGGGGTAGGTCAAGCTATACACAAACAGCCGTTACTCTCTGCCCCTAAGGCCTTCGAGAAAATTCCAACCACCCCTCTCTGTTAATGCGTTAGCGCTATCGCGCTCGCATTCGCACTCACTCGCAAACACAACGAGGGGAAATGACATTCGAGTCGATTCGAGAGAGCGCGTCGAGAAATTTCGAACAGAGAGCGCCAGAGTATAAATACGGCAGAAGTTCGCTCTTTGGCTCA

ATTCAATTCAAGCAAGTGAACTGAAAACGTCGAAAGACAAAAGCGAAATACAAACAAGCG

-269 -255 3

-183 -169 3

-88 -69 4

-65 -51 3

>Hsp70_D_mojavensis_C

GTTGTGTTCAGATGGCCATTCTGTATGACATGCCCAAGGTACATATAAAAAGAGTACTGTAATAGCTCTATCATTTCAATCTAAGTGCATGGTGCACAACTTCACAAATGCATGTATTTGTACATGTATGTATGTACATATGTATTTCTTTGTACATACATACATACATATTAATGTTAACAGGTATTGGTCAGTGAAACTCATCCATCATTTAGATTAGTGTGCACACGTGATTCCCCGTATATGCTTCGATTTTGAAACGTGATGTCGAAAACAAGTCTGTTAAGTCAGCATTCGCTTATTCAAGTATGAGAATACTTAGAAGCACCAATGAATTTTCTAGAAATTGAAATGATAGCCAATTGTTGGGGTAGGTCAAGCTATACACAAACAGCCGTTACTCTCTGCCCCTAAGGCCTTCGAGAAAATTCCAACCACCCCTCTCTGTTAATGCGTTAGCGCTATCGCGCTCGCATTCGCACTCACTCGCAAACACAACGAGGGGAAATGACATTCGAGTCGATTCGAGAGAGCGCGTCGAGAAATTTCGAACAGAGAGCGCCAGAGTATAAATACGGCAGAAGTTCGCTCTTTGGCTCA

ATTCAATTCAAGCAAGTGAACTGAAAACGTCGAAAGACAAAAGCGAAATACAAACAAGCG

-269 -255 3

-183 -169 3

-88 -69 4

-65 -51 3

>Hsp70_D_mojavensis_D

TCCGCCAATAAAAAACTTCAAATATTAATATTAAAGCATATTTCATGTATACATACATACATATGTATGTATACATACATACATATGTATGTATATCCATACATACATATATATGTGTGCACATACATATGTATGTACATATGTATGTATGTAAGAGTATATGTACATACATATATACACATGAACATACATACAAACATACATATGTCTGTGTTTGACTGCCAAACACGAGTTCCACTGTATATGCTTCGATTTTGAAACGTGATGTCGAAAACAAGTCTGTTAAGTCAGCATTCGCTTATTCAAGTATGAGAATACTTAGAAGCACCAATGAATTTTCTAGAAATTGAAATGATAGCCAATTGTTGGGGTAGGTCAAGCTATACACAAACAGCCGTTACTCTCTGCCCCTAAGGCCTTCGAGAAAATTCCAACCACCCCTCTCTGTTAATGCGTTAGCGCTATCGCGCTCGCATTCGCACTCACTCGCAAACACAACGAGGGGAAATGACATTCGAGTCGATTCGAGAGAGCGCGTCGAGAAATTTCGAACAGAGAGCGCCAGAGTATAAATACGGCAGAAGTTCGCTCTTTGGCTCA

ATTCAATTCAAGCAAGTGAACTGAAAACGTCGAAAGACAGAAGCGAAATACAAACAAGCG

-269 -255 3

-183 -169 3

-88 -69 4

-65 -51 3

>Hsp70_D_mojavensis_E

AGTCAGAGTACGGTGACTATTGTAAACTTACAGACTTATGTGCAAATTTTTCGTTTTTTAGTGGAAATGAAAAAAATAATACATACATATATAATATTCCATACACATATGTACATATGTATGTACATACAGTGACGAACGGTGATTTTCTTGCAGAGTGGGTATTTGAAACACATTATATGCGTCTATGTATATGTACATTATATATACATTCATATGTACACACTAATATATGTGTTTCCACATATACATATACATATGCGTATGTATGTGGCCGTGATATCCAAAAGCAAACTGCCAAGTCAGCATTCTCGAATTTAATAGCGCAAACGAATTTTCTAGATATTAATATCGCAGCCAACTGTTACGGTAGGTCGAGTGATACACAAACAACCGTTACTGTCTTGGAGCATACCAATATGAGGGAATATTCTAGAATATTCCAACCACTCCCACAGTAAGCAACAGCCGCTCTCACTCACTCACACTCGAAAAAACAGTCGAGTCGATTCAGTCGAGTCGATTCGAGAGAGAGCGTCGAGAAATTTCGAACAGAGAGCGCCAGAGTATAAATACGGCAGAAGTTCGCTCTTTGGCTCA

ATTCAATTCAATCAAGTGAACTGAAAACGTCGAAAGACAAAAGCGGAATACAAACAAGCG

-270 -256 3

-176 -157 4

-88 -69 4

-65 -51 3

>Hsp70_D_mojavensis_F

TCCGCCAATAAAAAACTTCAAATATTAATATTAAAGCATATTTCATGTATACATACATACATATGTATGTATACATACATACATATGTATGTATATCCATACATACATATATATGTGTGCACATACATATGTATGTACATATGTATGTATGTAAGAGTATATGTACATACATATATACACATGAACATACATACAAACATACATATGTCTGTGTTTGACTGCCAAACACGAGTTCCACTGTATATGCTTCGATTTTGAAACGTGATGTCGAAAACAAGTCTGTTAAGTCAGCATTCGCTTATTCAAGTATGAGAATACTTAGAAGCACCAATGAATTTTCTAGAAATTGAAATGATAGCCAATTGTTGGGGTAGGTCAAGCTATACACAAACAGCCGTTACTCTCTGCCCCTAAGGCCTTCGAGAAAATTCCAACCACCCCTCTCTGTTAATGCGTTAGCGCTATCGCGCTCGCATTCGCACTCACTCGCAAACACAACGAGGGGAAATGACATTCGAGTCGATTCGAGAGAGCGCGTCGAGAAATTTCGAACAGAGAGCGCCAGAGTATAAATACGGCAGAAGTTCGCTCTTTGGCTCA

ATTCAATTCAAGCAAGTGAACTGAAAACGTCGAAAGACAAAAGCGAAATACAAACAAGCG

-269 -255 3

-183 -169 3

-88 -69 4

-65 -51 3

>Hsp70_D_virilis_A

ACAAAAGTGTTGGCACAGCAAGCATATTCTACTTTGCAATCATGCATAAAAATAAATCGAGTAAAAACTAAATGGTCAGATACATATGTATGTATGTAATTATATGTATGCATATACATATGTACATGTAGTTGGGAATACAGTGACAACTTTCATGCCTATACTCTAGGAGCGTATTTACTATTAAGCGTTAGCACATTTATATACTGGCAAATAAAATGTCAAGAAATTGTTTTGACAAAGCGAAAAATTCATATTTTCCAGATTGTACATTTATTAATTGAAAATAGTTAAATATAACAAAATTATATATTTTTGCCAACAATCTTTAAGTTCGTGTGAATTTTCTAGAATGGATAAAAGGTAAATACCAATTGTTGCCGCAGGTCAAGTAAAACTCTCTCTCACTCTTCTAAACAATAACAAACCCAACCTTCTAGAAAAATCGAAACATATTCGAGCTGTTCCCTCTCCCTCTAACACATAAGCAAATACATCGAATGTACGCGTCGTTCGACGCGTTTCGAGAAGAGAGCGTCGAGAAAATTCGAAAAGAGAGCGCCGGGGTATAAATACGGCTGAAATTTTCTCTTCGCCACA

ATTGAATTCAAGCAAGCGACGTGATAACATCACAAACCAAAACAAGCGACTAGCTGAACA

-261 -247 3

-167 -153 3

-89 -70 4

-65 -46 3

>Hsp70_D_virilis_B

AGCAAAAACTAGGGAGCCCAGTTCGGTTTATTTGCTATAGTGTATATATTTGTTGATTTACACGTCGTTAAAGCAGCAACAAACTTTAAATGACAACTAGATTAGCATTCACATGCATAGAACGAGTTTTTCGATTCAAACATATTTATGGACATGTATATGTATCTAAATTTGGTTCCAAAAATAGCCAATCAAGTCAGCATTCGCATTCGAATTCGCATTCGCACATAAATAAAATTCATACATCTATCTATTATTAGCATTTTGTTAAATATAACAAAATATAACAAAACAGTTGCCAACAATCTTTAAGTTCGTGTGAATTTTCTATAATGGAAAAGGTAAATACCAATTGTTGCTGTAGGTCAAAACGCACAACCGTTACTCTCTTTCCCTCTCGCTCTATCGGCAACACAGTAACAAAAGAAGGTTGGCTCTAGAAAAATCGAAGCATATTCGAGCTGTTCCCTCTCCCTCTAACACATAAGCAAACGCATCGAATGTACGCGTCGTTCGACGCGTTTCGAGAAGAGAGCGTCGAGAAATTTCGAAAAGAGAGCGCCGGGGTATAAATACGGCTGAAATTTTCTCTTCGCCACA

ATTGAATTCAAGCAAGCGACGTGATAACATCACAAACCAAAACAAGCGACTAGCTGAACA

-281 -267 3

-167 -153 3

-89 -70 4

-65 -46 4

>Hsp70_D_virilis_C

AGGGAGCCCAGTTCGGTTTATTTGCTATAGTGTATATATTTGTTGATTTACACGTCGTTAAAGCAGCAACAAACTTTAAATGACAACTAGATTAGCATTCACGTGCATAGAACGAGTTTTTCGATTCAAACATATTTATGGACATATGTACATATATGTATGTATCTAAATTTGGTTCCAAAAATAGCCAATCAAGTCTGCATTCGCATTCGAATTCGCATTCGCACATAAATAAAATTCATACATCTATCTATTATTAGCATTTTGTTAAATATAACAAAATATAACAAAACAGTTGCCAACAATCTTTAAGTTCGTGTGAATTTTCTATAATGGAAAAGGTAAATACCAATTGTTGCTGTAGGTCAAAACGCACAACCGTTACTCTCTTTCCCTCTCGCTCTATCGGCAACACAGTAACAAAAGAAGGTTGGCTCTAGAAAAATCGAAGCATATTCGAGCTGTTCCCTCTCCCTCTAACACATAAGCAAACGCATCGAATGTACGCGTCGTTCGACGCGTTTCGAGAAGAGAGCGTCGAGAAATTTCGAAAAGAGAGCGCCGGGGTATAAATACGGCTGAAATTTTCTCTTCGCCACA

ATTGAATTCAAGCAAGCGACGTGATAACATCACAAACCAAAACAAGCGACTAGCTGAACA

-281 -267 3

-167 -153 3

-89 -70 4

-65 -46 4

>Hsp70_D_virilis_D

AGGGAGCCCAGTTCGGTTTATTTGCTATAGTGTATATATTTGTTGATTTACACGTCGTTAAAGCAGCAACAAACTTTAAATGACAACTAGATTAGCATTCACGTGCATAGAACGAGTTTTTCGATTCAAACATATTTATGGACATATGTACATATATGTATGTATCTAAATTTGGTTCCAAAAATAGCCAATCAAGTCTGCATTCGCATTCGAATTCGCATTCGCACATAAATAAAATTCATACATCTATCTATTATTAGCATTTTGTTAAATATAACAAAATATAACAAAACAGTTGCCAACAATCTTTAAGTTCGTGTGAATTTTCTATAATGGAAAAGGTAAATACCAATTGTTGCTGTAGGTCAAAACGCACAACCGTTACTCTCTTTCCCTCTCGCTCTATCGGCAACACAGTAACAAAAGAAGGTTGGCTCTAGAAAAATCGAAGCATATTCGAGCTGTTCCCTCTCCCTCTAACACATAAGCAAACGCATCGAATGTACGCGTCGTTCGACGCGTTTCGAGAAGAGAGCGTCGAGAAATTTCGAAAAGAGAGCGCCGGGGTATAAATACGGCTGAAATTTTCTCTTCGCCACA

ATTGAATTCAAGCAAGCGACGTGATAACATCACAAACCAAAACAAGCGACTAGCTGAACA

-281 -267 3

-167 -153 3

-89 -70 4

-65 -46 4

>Hsp70_D_virilis_E

CTAGGGAGCCCAGTTCGGTTTATTTGCTATAGTGTATATATTTGTTGATTTACACGTCGTTAAAGCAGCAACAAACTTTAAATGACAACTAGATTAGCATTCACATGCATAGAACGAGTTTTTCGATTCAAACATATTTATGGACATATGTACATATGTATGTATCTAAATTTGGTTCCAAAAATAGCCAATTAAGTCAACATTCGCATTCGAATTCGCATTCGCACATAAATAAAATTCATACATCTATCTATTATTAGCATTTTGTTAAATATAACAAAATATAACAAAACAGTTGCCAACAATCTTTAAGTTCGTGTGAATTTTCTATAATGGAAAAGGTAAATACCAATTGTTGCTGTAGGTCAAAACGCACAACCGTTACTCTCTTTCCCTCTCGCTCTATCGGCAAAACAGTAACAAAAGAAGGTTGGCTCTAGAAAAATCGAAGCATATTCGAGCTGTTCCCTCTTCCTCTAACACATAAGCAAATACATCGAATGTACGCGTCGTTCGACGCGTTTCGAGAAGAGAGCGTCGAGAAATTTCGAAAAGAGAGCGCCGGGGTATAAATACGGCTGAAATTTTCTCTTCGCCACA

ATTGAATTCAAGCAAGCGACGTGATAACATCACAAACCAAAACAAGCGACTAGCTGAACA

-281 -267 3

-167 -153 3

-89 -70 4

-65 -46 4

>Hsp70_D_virilis_F

CAAAAACTAGGGAGCCCAGTTCGGTTTATTTGCTATAGTGTATATATTTGTTGATTTACACGTCGTTAAAGCAGCAACAAACTTTAAATGACAACTAGATTAGCATTCACGTGCATAGAACGAGTTTTTCGATTCAAACATATTTATGGACATGTATATGTATCTAAATTTGCTTCCAAAAATAGCCAATCAAGTCAGCATTCGCATTCGAATTCGCATTCGCACATAAATAAAATTCATACATCTATCTATTATTAGCATTTTGTTAAATATAACAAAATATAACAAAACAGTTGCCAACAATCTTTAAGTTCGTGTGAATTTTCTAGAATGGATAAAAGGTAAACACCAATTGTTGCTGTAGGTCAAAACGCACAACCGTTACTCTCTTTCCCTCTCGCTCTATCGGCAACACAGTAACAAAAGAAGGTTGGCTCTAGAAAAATCGAAGCATATTCGAGCTGTTCCCTCTCCCTCTAACACATAAGCAAACGCATCGAATGTACGCGTCGTTCGACGCGTTTCGAGAAGAGAGCGTCGAGAAATTTCGAAAAGAGAGCGCCGGGGTATAAATACGGCTGAAATTTTCTCTTCGCCACA

ATTGAATTCAAGCAAGCGACGTGATAACATCACAAACCAAAACAAGCGACTAGCTGAACA

-283 -269 3

-167 -153 3

-89 -70 4

-65 -46 4

>Hsp70_D_grimshawi_A

TTGTTATGAAAATAAGGAAATATAACAGCTGCTAAACTCGAATATGATGTGTGTATTAGTTGAGATAAGTTCACAGAACTCAGTCGTGTAACAAGCACAATAATAATCTTTAGAAGGTAACATCGAGATTAGCATTCACGTGCACGCAACAAGTTAATGAATATACTCATCTAGGAATCCAAAAATAGACAACAAGTCAGCAAAAATGTTTAATAATAGTAAACGTTTAATAATAAACTACATACATATATACATACAGATGGACTTTTTCTGCAGACTTAAGGTCGTTTGATGTGAACTTTCTAGAATTGTTGGGGTGGGTCAAGCTACACAAACAACTGTTAGTCTCAGTCGCTCTCGCTCTTTACACAAAAACCAAAATAAAGCAGACCTTCGAGAAAACTCGAAGCATTTCCATTTGGTTTCTCTCTCAGTCGACAGTCGACAGTCAAACACCATCTGCCTGCTATCTCTCTCCCGCTCATTCGAAATCGGTTGGAAGGTTCGCGACGTTCGACTCGTTTCGAGAAGAGAGCATCTAGAAATTTCGGAAAGAGACCGCTCGAGTATAAATACGGCCGGCATTTTCTCTTTGGCTCA

ATTCAATTCAAGCAATCGAAGTGATAACATCACAAACAAGCAACAGCCAAAAAAGCAAAA

-306 -292 3

-209 -195 3

-89 -70 4

-65 -46 4

>Hsp70_D_grimshawi_B
TTGTTATGAAAATAAGGAAATATAACAGCTGCTAAACTCGAATATGATGTGTGTATTAGTTGAGATAAGTTCACAGAACTCAGTCGTGTAACAAGCACAATAATAATCTTTAGAAGGTAACATCGAGATTAGCATTCACGTGCACGCAACAAGTTAATGAATATACTCATCTAGGAATCCAAAAATAGACAACAAGTCAGCAAAAATGTTTAATAATAGTAAACGTTTAATAATAAACTACATACATATATACATACAGATGGACTTTTTCTGCAGACTTAAGGTCGTTTGATGTGAACTTTCTAGAATTGTTGGGGTGGGTCAAGCTACACAAACAACTGTTAGTCTCAGTCGCTCTCGCTCTTTACACAAAAACCAAAATAAAGCAGACCTTCGAGAAAACTCGAAGCATTTCCATTTGGTTTCTCTCTCAGTCGACAGTCGACAGTCAAACACCATCTGCCTGCTATCTCTCTCCCGCTCATTCGAAATCGGTTGGAAGGTTCGCGACGTTCGACTCGTTTCGAGAAGAGAGCATCTAGAAATTTCGGAAAGAGACCGCTCGAGTATAAATACGGCCGGCATTTTCTCTTTGGCTCA

ATTCAATTCAAGCAATCGAAGTGATAACATCACAAACAAGCAACAGCCAAAAAAGCAAAA

-306 -292 3

-209 -195 3

-89 -70 4

-65 -46 4

>Hsp70_D_grimshawi_C

TGTATTGAAATGAGAATAATCTAAATACAAGTGTACAAACATACATATACTTTTATTGAAGTACATAAGTTCACAGAACTCAGTCGTGTAACAAGCACAATAATAATCTTTAGAAGGTAACATCGAGATTAGCATTCACGTGCACGCAACAAGTTAATGAATATACTCATCTAGGAATCCAAAAATAGACAACAAGTCAGCAAAAATGTTTAATAATAGTAAACGTTTAATAATAAACTACATACATATATACATACAGATGGACTTTTTCTGCAGACTTAAGGTCGTTTGATGTGAACTTTCTAGAATTGTTGGGGTGGGTCAAGCTACACAAACAACTGTTAGTCTCAGTCGCTCTCGCTCTTTACACAAAAACCAAAATAAAGCAGACCTTCGAGAAAACTCGAAGCATTTCCATTTGGTTTCTCTCTCAGTCGACAGTCGACAGTCAAACACCATCTGCCTGCTATCTCTCTCCCGCTCATTCGAAATCGGTTGGAAGGTTCGCGACGTTCGACTCGTTTCGAGAAGAGAGCATCTAGAAATTTCGGAAAGAGACCGCTCGAGTATAAATACGGCCGGCATTTTCTCTTTGGCTCA

ATTCAATTCAAGCAATCGAAGTGATAACATCACAAACAAGCAACAGCCAAAAAAGCAAAA

-306 -292 3

-209 -195 3

-89 -70 4

-65 -46 4

>hsp83_D_melanogaster

TTTTCCGGAATGTGAAATGTCTGCTTTTTAGCTAATTACAACAAAAACTTTCCAATTTTTGTTCCCCAAACCCACTCAAGTGATTTCAAATTTTACCGTCCGCTTAAAATGGAACTAGTTTATTCCAGAGGAACCAGCTTGCACCACCAAGTCTCTGAAACTCTGGAAATATCGATAGTCTGGTGGAGAAAAGTATTCATAAATATAAATAAAAATTAACAGGTCATAAGCTGATTTGTTTATTATTTACTGTTAAAACAAGTAAAATAATATTGGGAACAATTAAATTTTCCATTTTCCTAATTACAGTATAAGCCTAGTGGGCGTTTTGATATCCAATTGTAATGTTTTAAGCAATCCCAGTGGGCTTTGCTCAATCGTTCGGACCACTTAGACGAATTTCCACCAAACTTAGTTCAGTATAATTTTTGAATTCGCCCGCACAGGTTGCGCACTTTTCGACCGTATCACAACACTGATCTACCCTAGTATTCACAGGAAGTTGCATCCCTGGCATCCAGAAGCCTCTAGAAGTTTCTAGAGACTTCCAGTTCGGGTCGGGTTTTTCTATAAAAGCAGACGCGCGGCGTTTGCCGGTTC

GAGTCTTGAAAAAAATTTCGTACGGTGTGCGTCGTAACAACAAGCAGCGTCTGAAAAGTT

-86 -47 8

>hsp83_D_simulans

AATGTGATAGGTCTGCTTTTAAGCTAATTTACCAACAAAAACTTTCCAATTTGTTTCTTCCTAAACCCACTAAAGTGATTTCAAATTTTACCGTCCCCTTAAAATGGAACTAGTTTATTGCAGAGGAACCAGCACCGCCAAGTAAACGAGACTCTGGAAGTATCGATTGTCCGGTGGAGAAAAGTATTTATAAATATAAATAAAAAATTAACAGATCATAAATTAACAGATAAGATGTTTATTATTTACTTTTTAAAACCAGAAATATAATGGGAAAAATTTAATTTTCCATTTACCCAATTATAGTTTAAGCCTTAGTTAGGGCCTTTTGATAGCCAATTGTAATGTGTTAAGAAATCCCAGTGGGCTTTGCTCAATCGTTCGGACCACTTAGACGAATTTCCACCCAACTTAGTTCAGTACAATTTTTGAACTCGCCCGCACAGGTTGCGCACTTTTCGAACGTATCACAACACTGATCTACCCCAGTATTCACAGGAAGTTGCATCCCTGGCAACCAGAAGCCTCTAGAAGTTTCTAGAGTCTTCGAGTTCGGGTCGGTTTTTTCTATAAAAGCGGACGCGCGGCGTTTGCCGGTTT

GAGTCTTGAAAAAAATTTCGTGCGGTGTGCGTCGTTACAGCAAACAGCGTCTGAAAAGTT

-86 -47 8

>hsp83_D_sechellia

AATGTGATAGGTCTGCTTTTAAGCTAATTTACCAACAAAAACTTTCCAATTTGTTTCTTCCTAAACCCACTCAAGTGATTTCAAATTTTACCGTCCCCTTAAAATGGAACTAGTTTATTCCAGAGGAACCAGCACCGCCAAGTCTCTGATACTCTGGAAGTATCGATTGTCCGGTGGAGAAAAGTATTTATAAATATAAATAAAAAATTAACAGATCATAAATTAACAGATAAGATGTTTATTATTTACTTTTTAAAACCAGAAATATAATGGGAAAAATTTAATTTTCCATTTACCCAATTTCAGTTTAAGTCTTAGTTAGGGCCTTTTGATATCCAATTGTAATGTGTTAAGAAATCCCAGTGGGCTTTGCTCAATCGTTCGGACCACTTAGACGAATTTCCACCCAACTTAGTTCAGTACAATTTTTGAATTCGCCCGCACAGGTTGCGCACTTTTCTAACGTATCACAACACTGATCTACCCTAGTATTCACAGGAAGTTGCATCCCTGGCAACCAGAAGCCTCTAGAAGTTTCTAGAGTCTTCGAGTTCGGGTCGGTTTTTTCTATAAAAGCAGACGCGCGGCGTTTGCCGGTTT

GAGTCTTGAAAAAAATTTCGTGCGGTGTGCGTCGTAACAGCAAACAGCGTCTGAAAAGTT

-86 -47 8

>hsp83_D_yakuba

TTTACCAAAACGAAATGATCAACGATTTGATACCTCTTAATTCGGGTAAAAAACCGTTGACAATGGGTTATGGGTTTTGATTTATTTATTCAATAATGGAATAAAAAGATTTTACCAAATAAGGATTGACCCAGATAAACACTGAAAACTCCGTCGATATTTGATAGTCCGGTGGACAAAAATAAACATAAACATTGTACAATAAAATAAATCAGATCATAAGCTAGACATTTTTAAATTAATTTGTTTTTAAAACAAAACAAATAAGTTAAGTTGAAAAAATTAAATTGTCGCTCTATTTGAATACAATACAAACCTAGTGGCCGTTTTTGTAACCAATGGTGTTATTTTAAGCAGTCCAAGTGGGCCGTGGTCAATCGTTCCGACCATTTAGACGAGTTTCCATCAAACATAGTTCAGTACAATTTTTGAATTCGCCCGCACAGGTTGCGCTCTTTTCGACCGTATTACAACACTGATCTCCCTGAGTACGCACAGGAAATTGCATCCCTGGCATCTGGAAGCCTCTAGAAGTTTCTAGACTCTTCGAGTTCGGGTCGGATTTTTCTATAAAAGCCGACGCGCGTCGTTTGCCGGTTC

GAGTCTTAAAAAAATTTCGTGAGGTGTGCGTCGTTACAGCAAGCAGCGTCTGAAGTTTTG

-86 -47 8

>hsp83_D_santomea

ATATGTGAATTTACCAAAACGAAATGATCAATGATTTGATACTTCTTAATTCGGGTAAAAAACCGTTGACAATGGGTTATGGGTTTTGATTTATTTATTCAATAATGGAATAAAAAGGTTTTATAAAATAAGGATTGACCCAGATAAGCACTAAAATACTCTGAAACTCTGTCGATATTTGACAGTCCGGTGGACAAAAATTAACATAAACATTGTACAATAAAATAAATCAGATTGTAAGCTAGACTTTTTTAAATTAATTTGTTTTCAAAACAAAACAAATAAGTTAAGTTGGAAAAATTAAATTATTTGAATACAATTGAAACCTAGTGGTGGTTTTTTTAATATTTTCAACAGTCCCAGTGGGCCGTGCTCAACCGTTCCGACCATTTAGACGAGTTTCCATTAAACATAGTTCATTACAATTTTTGAATTCGCCCGCACAGGTTGCGCTCTTTTTGACCGTATTACAACACTGATCTCCCTTAGTACGTACAGGAAATTGCATCCCTGGCATCTGGAAGCCTCTAGAAGTTTCTAGACTCTTCGAGTTCGGGTCGGATTTTTCTATAAAAGCCGACGCGCGTCGTTTGCCGGTTC

-86 -47 8

>hsp83_D_erecta

CTGTTTTTAAACAACTTAACCAGGAAATCCTTCTAATATTTTTTCTTCCAAAAACCACTCAAGCCATTTCAAATTTTACTTTCAGCTTAAAATTGATCTAGTTTATTCCACAGGAACCAACTTGCTCCACCGAACTGCTCCACCGAAACTCTGGAAATATCGATAGTCTGGTGGGAAAAAGTAAACAAGCTTGAAACGAAGCAAACAAATTTCAAATCATATGCTGGGCATATTTACATTATTTTGTTTTCAGAACAAAACAAATAAATTAGGTTGGGTGAAAATAAAATTTTCTTTATTTAATCACAATGTAAACCTAGTGGGCCTTTTTATTACCAATGGGAGTATTTTAAGTAATCCCAGTGGGCTGTGCTCAATCGTTCAGACCATTTAGACGAATTTCCACCAAACATAGTTCAGTACAATTTTTGAATTCGCCCGCACAGGTTGCGCACTTTTCGACCGTATTACAACACTGATCTACGTTAATATTCACAGGAAATTGCATCCCTGGCATCTGGAAGACTCTAGAAGTTTCTGGACGCTTCGATTTCGGGTCGGATTTTTCTATAAAAGCAGACTCGCGGCGTTCGCCGGTTC

GAGTCTTGAAAAAATTTCGTGCAGTGCGCGTCGTAGCAGCAAATAGCGTCTGAAGTTTTG

-86 -52 7

>hsp83_D_ananassae

NNNNNNNNNNNNNNNNNNNNNNNNNNNNNNNNNNNNNNNNNNNNNNNNNNNNNNNNNNNNNNNNNNNNNNNNNNNNNNNNNNNNNNNNNNNNNNNNNNNNNNNNNNNNNNNNNNNNNNNNNNNNNNNNNNNNNNNNNNNNNNNNNNNNNNNNNNNNNNNNNNNNNNNNNNNNNNNNNNNNNNNNNNNNNNNNNNNNNNNNNNNNNNNNNNNNNNNNNNNNNNNGTCGCCCTCATAGGATTCGTACTTTTCGAATGATTTCAACGTTTATATAAAAAAAGTCGTATATAAATTTTAAGAAAACATTTTCATCAACAAATAAGTGTATATAAAAAAAAAAACAAACTGGCAAGTGACATTTTTTCTAAATTATTTCTTCTAAATTTAAATATTAACGAAAATTTATTGCAGCCCTTGGAGTCGGCAATTTTTGAATTTTCAATTGTGCCCGCTCACTTTTCGCGGTGACCAACACTGATTTGCCCGGAAGTGTGGTCCCTGGCGCGTAGAAAGATCTGGAATCGTCTAGAAGCTTCGGTGTATGTATGTGTGTGGCCTGTACGCTGCTGTATAAAACCAGGGGTTGTGCCGCCGACGGTGTT

GAGTCTTGAAAAAATTTTCGTTGAATCCGCCACTCTTGCGGCTGAAGTTGTGCAGAAGCA

-95 -66 6

>hsp83_D_pseudoobscura

CAATACCCAAGTATTTGAATTTTCCATCTCTCATCGGGGGTAATTCATGAACCGGTTCCAGCCGAAAAATGAACGAAATTCATGAGAGATTATTTTTTCGGGATTGCTTGCCATATAAAATACATTTCGGAAAAACAAAATGTACTACATTTTTGTCATCTCAAGGTGCTCCAATTAATTATGAATGCTACGAAACTACAAAGCAGCTTGGAAATCCGAATTTTAACAATAATTAAAGGAAATAGGGTATAGCGTATATAGGGTATCATAGCTGAAACGGGTATACCAACAATAATGACGCAGCACTTACGTTTCACTCCGTACTCACTTACGATTTATGCTTATAATTTTTGTTCACCTTTTTTACTTAAACCTCACTTTAAAAACAATCAAATAAATGGGAGTATTTATGTATATTTCTAAGATTACGGCGGTATTGTTCTGCGGTATGCGGTCACACTGGTTTACAGCCTCGGTGCAACTCTGTGCCAGTACCGGAAATAGCAGCCCTGGATTCTCGTAGCCTCTAGAAACGTCTAGAAAATTCTACGCTTGGGGTTGGTTTGCTATAAAAGCAGGCGGGCCGACTGTTGCCGGCTC

GAGTCTTGAAAAATTTTTGTCCAGTGAAGGTGCGTTTGCTTAGAGCGCAGTGCAACAAAG

-87 -53 7

>hsp83_D_persimilis

AATACCCAAGTATTTGAATTTTCCATCTCTCATCGGGGGTAATTCATGAACCGGTTCCAGCCGAAAAATGAATGAAATTCATGAGAGATTATTTTTTCGGGATTGCTTGCCATATAAAAATACATTTCGGAAAAACAAAATGTACTACATTTTTGTCATCTCAGGGTGCTCCAATTAATTATGAATGCTACGAAACTACAAAGCAGCTTGGAAATCCGAATTTTAACAATAATTAAAGGAAATAGGGTATAGCGTATATAGGGTATCATAGCTGAAACGGGTATACCAACAATAATGACGCAGCACTTACGTTTCACTCCGTACTCACTTACGATTTATGCTTATAATTTTTGTTCACCTCTTTTACTTAAACCTCACTTTAAACACAATCAAATAAATGGGAGTATTTATGTATATTTCTAAGATTACGGCGGTATTGTTCTGCGGTCTGCGGTCACACTGGTTTTCAGCCTCGGTGCAACTCTGTGCCAGTACCGGAAATAGCAGCCCTGGATTCTCGTAGCCTCTAGAAACGTCTAGAAAATTCTACGCTTGGGGTTGGTTTGCTATAAAAGCAGGCGGGCCGACTGTTGCCGGCTC

GAGTCTTGAAAAATTTTTGTCCAGTGAAGGTGCGTTTGCTTAGAGCGCAGTGCAACAAAG

-87 -53 7

>hsp83_D_willistoni

TGTATTATTAGTGGGCTATCTGTTGATTCCATTTGAACTTTTAACTCTATTTTCGGCTGTTGATCCTGATCAAGAATATATATACCTTAAGGGGTCGGAGAAGCTTTCTTCTGCCTGTTATACACAGGCTTGAGACCTTTGTCGAACTCCAACCAGTGCTAAGGTCTCAGTAAAAGTCCTAAGGGTCCGAGTAAAAGTCCTAAGGGTCCGAGTAAAAAGTCCTAACGGTTAACAGTCCAGCTTATGTTAACTTTTAACATTAACATGGGTAAATGTCAAATTTAACAGTCACCATTTCCCATTATTGCAAATTTGGCGCTAAATATCTATTACATAAATATTGTTATTAAAGGTAAATAATTTATTAACTCATTTGTTGTTCATTTTGTTAAACAAATCAACATTTTAGGCGCAATAATTTTTGCAAATATATAAAAATGTCTGTTAATTGTTAACATAAGCCCTGACAAGTTGGCAATTTACCAACACTAACAGGAATTGACTGCAGCCCTCAATTCTCGAACAGTCTAGAACATTCTTGAAATTTCTACGCTTGGGCCGATTTTTCTATAAAAGCAGGCCGTCCGACATATGCCGGTC

GAGTCTTTAAACAAATTTCGTACGTTGAACATCGTTGAGCACTTCGCTCTTGTTTTGTGT

-86 -52 7

>hsp83_D_mojavensis

TGCATTTTGGGATTGCGTTGTGTTGAGACTTGGCAGTCACGAAATACGCATCGCGTGCCACCCATTTTATCATAAGATTCTCTAATTTGCTCTTATTATTTTAAACACAACATAAACAAAATGCTATTAAGTTTGGTACTAAATTATAGGCTAACGCAATCTGGGCACACTTTTTATTTTTACCAAGTTTGGTCACCCAAAATTACGGCAGGTCAAAAAATATACTGAAAAGTTCAAATTTGATTAAAATTATGACATTATGAAAATACTGAAGAAAAATTAAAGGTTGTTTATTTGTTTATACTATCCAAATTATTATTATTGAACTAAATACTCAAGAGTGATGTCAAAATAAACAATAAAAGTATTTAATAATAACATTAACATGGTTCAATATTAGTGGAATATAAATTTATTTGAACATAGTTTCTAGAACATTACAGAAACATCGACTCACACTAATGTCAGTTGGCTCAATGCAACACTGCGTTGAAAACCGGAAGAGGTAGCCCTGACGACTAGATGTTTCTCGAAAAATCTAGAAGCTTCGTAGAATTTTGTTCAACCCCTATAAAAGCAGTAGCAAGCAGACGCCGGCTT

GTCAGTATTGAAACAAAATTCGTGCGGTGAGCATAAAAAGAGTCTGCCCTGAGGCAAAGC

-174 -150 5

-85 -51 7

>hsp83_D_virilis

AAAATGCATCTTCGGATTACGCTGTGTTGAGACTTGACAGTCGCGAAATACGCAGCGAGTGCCACCCATGTTAATATATAATCAGTCCACTTATTCCTGAATTAACAACGGCGTAAACAAAAAAAACGCTTTACTAAATTAGTTCCAAAATACACATAAATAAAAGGTCGGGCACACTTTATGTTCATCACAAGTTTGGCACTAAAATACCAAAGAAAAACGAAAAATATACCAAAAGAACGCATAGACTAGGAATACAAATAATTTGAAATATTTATTAAATTATACTTTTCTTATGTATTGCAGATTATACAATGTTTGTGCTAAAGGTTATTAATATGCCAATAGTATTTATGGGCTTATTAAATGACCTCCGATTTTTTTTATATAATACTGAAACCAACAGATCCCAAACATTAAACATATTTTCTAGAACTTTACAAAACCATCGACCTACAACACTGTCAGTTGTCTCCATGCAACAATGCGTTGATAACCGGAAGAGGTAGCCCTGGCGACGAGAAGTCTCTAGAAGTGTCTAGAAGTTTCGAGGCATTTTGTGCACCCTCTATAAAAGCAGCAGTAAGCAGATGCCGGCTT

GTCAGTATTGAAACAAAATTCGTGCGGTGAGCATTGTGAAGCGTCTGCTTGAAAGCAAAG

-174 -150 5

-85 -46 8

>hsp83_D_grimshawi

CAGCAGAACTGGATCACGAACTGGTAGTTTGAAAAAGTGCATTTTCGGATTGCGCTGTGATGAGACATGACAATCCCGGAAAATGCATCGAGTACCGCCCATTTTCTCATATAATTCTCTTCTTTGTAATTCAAAAACACAAAACAAGCTAAATGCATCCGCGACGTCAGCTATAGCGCTAAAATACACAAATAAACAAAGTCGAGCGCAATTGTGCGCAGTTTGGTCACTCTAAATCCCAAAAAAAAAATACTAAAACCACTTACAGTAATTTTTTAATCTGACTTATTATTAAAAAATAAATGTTTTAAACTATGTATTTTAAAATACATAAATTACAATCTTCAAACCACCCCCAAAATAATTATAGATATAAATAAATGTTGATGTAAATATATTTGTAAATTATTAATGGTACCTTATAGCTTCTAGAACATTACAAGTTCAACCATTTACAGCACTGTCAGTGGAAACGGTGCAACACTGAGTTCACAACCGGAAGAGGTAGCCCTGGGGACTAGAAGGCTCTAGAAGCGTCTAGAAGTTTCATAGCATTTGTGTGCACCCTCTATAAAAGCAGCAGCAAGCCATTGCCGGCTT

GTCAGTATAGAAGAAAATTTCGTGCGGTGAGCGTCTGTTTAAGGACAAAAAACAAAGCGA

-175 -151 5

-86 -52 7
